# Supplementary material for: The evolution of built-up areas in Ghana since 1975
Source: PLoS One. 2021 May 21;16(5):e0250847. doi: 10.1371/journal.pone.0250847 (PMC8139467; doi:10.1371/journal.pone.0250847)
Supplement: S1 Appendix — (DOCX) [file pone.0250847.s001.docx]

**Appendix A. Figures and Table accompanying the Conceptual Section**

The left panel of Figure A1 simulates the random distribution of rural human settlements in a low population density setting. It was produced by randomly placing densely built small villages and their lightly built-up hinterland over a large area, sparsely populated area. Each dot represents a building or built-up area. The longitude and latitude axes represent arbitrary units used in the simulation. The right panel of Figure A1 presents the spatial correlogram of all the built-up areas in the left panel.

The left panel of Figure A2 simulates the random distribution of rural human settlements in a high population density setting. It was produced by placing densely built small villages and their lightly built-up hinterland in a honeycomb pattern where the limits of their hinterland slightly overlap. Each dot represents a building or built-up area. The longitude and latitude axes represent arbitrary units used in the simulation. The right panel of Figure A2 presents the spatial correlogram of all the built-up areas in the left panel.

Figure A3 illustrates a simulation in which a town grows by crowding its hinterland. The diameter of the low-density hinterland is kept constant while the diameter of the densely built town grows over time. The longitude and latitude axes represent arbitrary units.

Figure A4 is obtained by dividing each of the four sub-graphs (i.e., small town, medium town, large town, and city) in Figure A3 into small squares or cells, and computing the percentage buildup in each cell. Figure A4 presents the distribution this buildup percentage across the four sub-graphs. As the town grows, the proportion of unbuilt and lightly built cells falls, the proportion of densely built cells increases, and the proportion of partially built-up cells remains stable.

**Appendix B. Checking whether Zipf Law applies to partial buildup**

Notes: Figure A1 shows a non-parametric regression line of the rank of a cell in terms of buildup on its buildup in level. Figure A2 shows a non-parametric regression line of the log of the rank of a cell in terms of buildup on the log of its buildup. All cells with zero buildup (the majority) are omitted from these regressions. Each non-parametric regression is estimated using a fractional polynomial

**Appendix C: Ghana**

Ghana is a West African country located on the [Gulf of Guinea](https://en.wikipedia.org/wiki/Gulf_of_Guinea), only a few degrees north of the [Equato](https://en.wikipedia.org/wiki/Equator)r. Historically, the country was one of the prosperous countries in Africa because of its rich natural resources particularly gold. The abundance of gold attracted Portuguese, Dutch, English, and French traders to the country during the 15^th^ century. Beginning in the 17th century — in addition to the gold trade —European traders also participated in the [Atlantic slave trade](https://en.wikipedia.org/wiki/Atlantic_slave_trade) in this area. After prolonged wars between British colonists and Ghana’s Ashanti kingdom, much of the territory around Ghana was brought under British colonial rules during early 1900s. In 1957, four British colonies ([Gold Coast](https://en.wikipedia.org/wiki/Gold_Coast_(region)), [Ashanti](https://en.wikipedia.org/wiki/Ashanti_(Crown_Colony)), the [Northern Territories](https://en.wikipedia.org/wiki/Northern_Territories_of_the_Gold_Coast_(British_protectorate)) and [British Togoland](https://en.wikipedia.org/wiki/British_Togoland) ) were unified to form the independent country of Ghana.

The landscape of Ghana is characterized by low physical relief with its highest elevation about 880m above sea level. The country has five distinct agro-ecological zones (Figure C1). The coastal savannah ranges around its 537 km of coastline. A [tropical rain forest](https://en.wikipedia.org/wiki/Tropical_rain_forest) belt, broken by heavily forested hills and many streams and rivers, extends northward from the shore, near the [Ivory Coast](https://en.wikipedia.org/wiki/Ivory_Coast) frontier. The general terrain in the northern and northwestern part of Ghana outside the Volta Basin consists of a dissected plateau, which averages between 150 and 300 meters in elevation and, in some places, is even higher. This region is home to a vast upland savannah. The region surrounding the largest man-made lake Volta accounts for about 45 percent of Ghana’s land area. This region extending from deciduous forest in the south to savannah in the north remains poor in terms of road infrastructure. The diversity of agro-ecological zones and terrain resulted in differences in agricultural practices and crops as well as human settlements.

**Figure C1: Main Agroecological zones of Ghana**


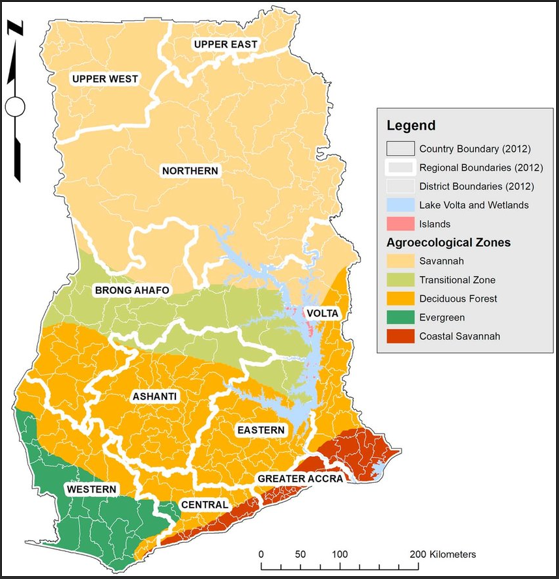


Ghana is currently classified as a lower middle-income country with a GDP per capita of $5637 at purchasing power parity in 2019 (World Bank). With a land area of 238,534 square km, Ghana is comparable to US state of Oregon in terms of land size. Ghana’s population increased from 6.6 million in 1960 to 30 million in 2019. As a result of rapid population increase, population density increased from 28/sqkm in 1960 to 130/sqkm in 2019. Figure C3 maps the regional differences in population density in Ghana. Population density is highest in Greater Accra region, followed by central, Ashanti, and Volta regions.

Source: WDI

Source: WDI

**Figure C3: Population density of Ghana by regions**


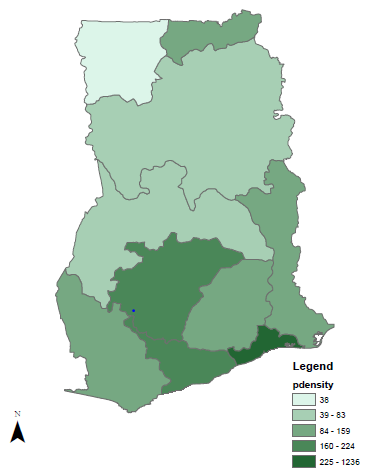


Source: Census 2010

From mid 1980s, Ghana experienced rapid increase in its urban population. In 1975, about 30% of Ghana’s population was urbanized which increased to 32% in 1984. By 2019, urban share of total population increased to 57 percent, making it as one of the most urbanized African countries. All regions of the country have experienced steady urbanization. In 2000, Ghana was a country of a few limited metropolitan areas and many small towns. Since then, all city types have dramatically increased in number, and Ghana has experienced faster urban population growth in its smaller cities than its larger ones. The number of medium (20,000–50,000 people) and large medium (50,000–100,000) sized towns has quadrupled and tripled, respectively. In 2000, there were only nine towns with population between 50,000 and 100,000; by 2010 the number had quadruped to 36. Ghana has two cities with more than a million of population: Accra and Kumasi. While Accra has grown considerably, its urban primacy has diminished: its 24.4 percent share of the total urban population in 1984 declined to 16.6 percent by 2010, representing more balanced urban growth that has moved from Accra alone to Accra plus Kumasi, port cities, and smaller cities (World Bank, 2015).


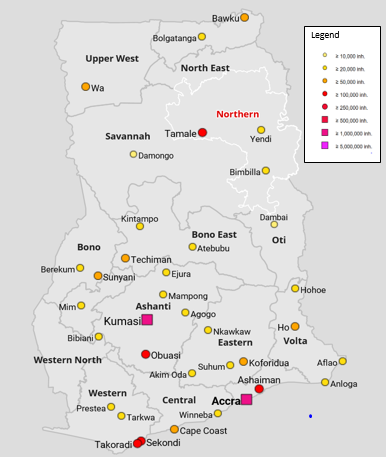


**Figure C4: Main cities and their population**

Source: <https://www.citypopulation.de/en/ghana/cities/>

The rapid urbanization in Ghana is also associated with structural transformation, economic growth and poverty reduction. According to 1970 census, more than three-quarters of its population was engaged in agriculture, animal husbandry and fishing which declined to 50% in 2010. By 2019, half of Ghana’s workers are employed in services and another 20% in industry (Figure C5). Three main exports from Ghana are gold (7^th^ largest producer), cocoa (second largest) and timber. Only recently, employment in industry has picked up as Ghana moved into more modern manufacturing. During this period of rapid urbanization, Ghana also experienced robust growth of GDP in recent years. From 1961 to 1983, GDP growth averaged 0.9 percent annually, from 1984 to 2013 it averaged 5.7 percent annually, and from 2005 to 2013 GDP growth averaged 7.8 percent (World Bank, 2015). Rapid economic growth has resulted in a reduction in poverty in both rural and urban areas, with the total poverty incidence dropping below 25 percent in 2013 from 56.5% in 1992. Rural poverty incidence still is much higher (37.9%) than in urban areas (10.6%).

Source: WDI

**References**

World Bank Group. 2015. Rising through Cities in Ghana: Ghana Urbanization Review Overview Report. World Bank, Washington, DC. <https://openknowledge.worldbank.org/handle/10986/22020>

**Appendix D: Data Appendix**

**Global Human Settlement Layer (GHSL)**: These datasets that have been put together by the Joint Research Centre of European Commission and is our main data source. The GHS-BUILT data, a part of the GHSL datasets, contain a multitemporal information layer on built-up presence as derived from Landsat image collections (GLS1975, GLS1990, GLS2000, and ad-hoc Landsat 8 collection 2013/2014). The methodology for construction of these datasets is described in Pesaresi et al (2016)*.* The dataset provides built-up information at three resolutions: 38m, 250m and 1km. At 38m resolution, the dataset identifies presence or absence of built-up in a 38X38 m^2^ which is then aggregated at 250km and 1km resolutions. The details of this dataset can be found in [https://ghsl.jrc.ec.europa.eu/index.php. In Figure D1](https://ghsl.jrc.ec.europa.eu/index.php.%20In%20Figure%20D1) below, we plotted the extracted data for Ghana and for the city of Kumasi.

Figure D1: Built-up Density in Ghana (left) and city of Kumasi (right) between 1975-2014


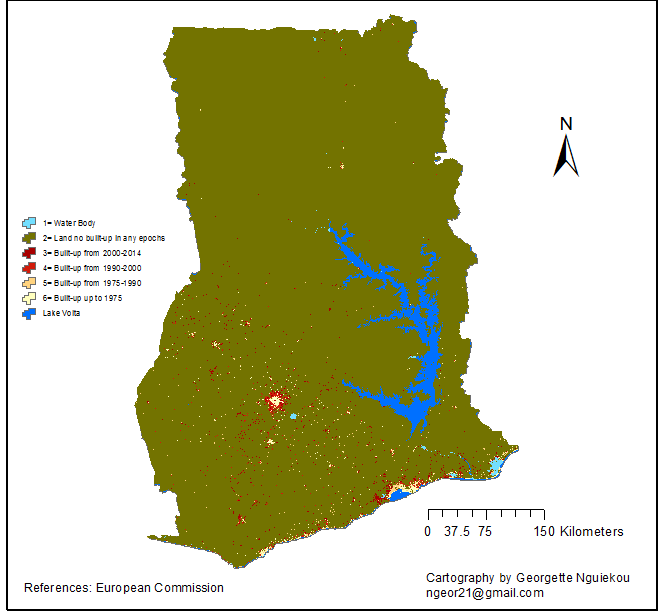

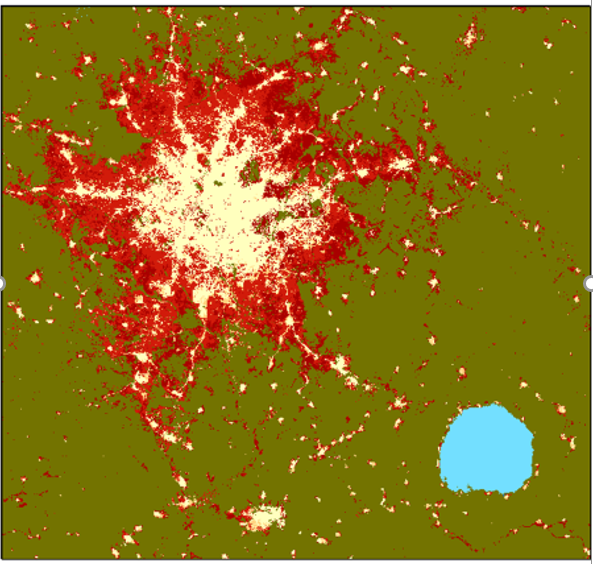


To extract built-up information for Ghana, we overlay Ghana shapefile over GHSL dataset. For each 250X250m cell, we extract the proportion built in 1975, 1990, 2000 and 2014 to form a panel dataset at cell level. These cell coordinates are then fixed to extract and merge in other data using spatial join function. The GHSL dataset is also used to find whether a cell belongs to water body (e.g. lake Volta). The data extraction is done by a program written in R.

**Ghana Shapefiles**: These files are obtained from the Ghana Statistical Services. The shapefiles are used to determine international it borders. We also extract information on whether a GHSL cell falls on international border or sea border from the shapefiles.

**Global Agro-Ecological Zones**: The data for agro-ecological zones are downloaded from the GAEZ-Global Agro-Ecological Zones data portal which was developed by the Land and Water Division of the Natural Resources Management and Environment Department of FAO. The resolution for GAEZ dataset is 10 km. We overlay 250 by 250 meters grids of GHSL dataset over GAEZ dataset to extract dominant soil types for our panel of cells. The dataset can be downloaded from <https://climate-adapt.eea.europa.eu/metadata/portals/gaez-global-agro-ecological-zones>. The main soil categories are steep terrain, arctic/cold, desert/arid, irrigated soils, hydromorphic soils, dry (good, moderate, poor), moist (good, moderate, poor), semi-humid (good, moderate, poor), humid (good, moderate, poor) and water. For each cell, we define a dummy variable to indicate the dominant soil type. Figure D2 below shows the map of soil types in Ghana.

**Figure D2**

**Ghana: Soil types at pixel level**


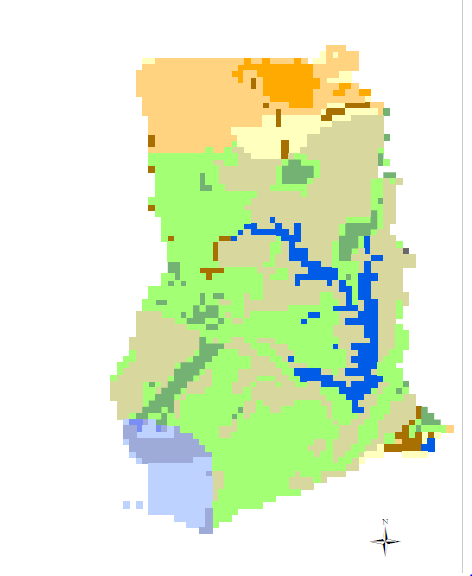


Legend


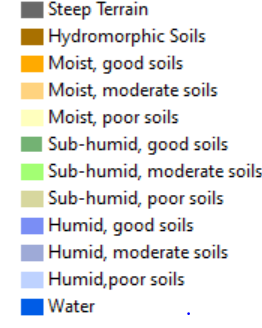


**Ghana Road networks**: The road network data comes from two sources. Road network for 2008 is derived from the data produced by the Africa Infrastructure Country Diagnostic (AICD) (World Bank, 2008) and our own digitization effort. For 1976 and 1986, we digitized high resolution road maps from censuses. Below are maps of road networks in 1976 and 1986. We overlay road maps on GHSL data to determine if a cell had road in it either in 1976 or 1986 or both.

Figure D3: Ghana Road network over time


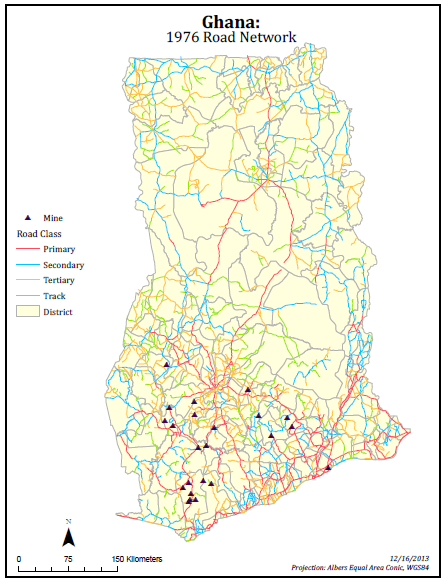

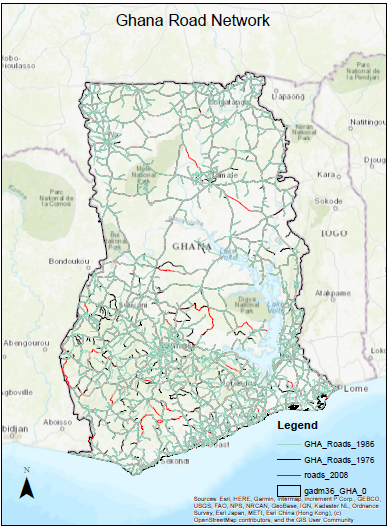


**References**

Gwilliam, Ken, Vivien Foster, Rodrigo Archondo-Callao, Cecilia Briceño-Garmendia, Alberto Nogales, and Kavita Sethi, *Africa Infrastructure Country Diagnostic: Roads in Sub-Saharan Africa,* World Bank, 2008.

Pesaresi M., Ehrlich D., Ferri S., Florczyk A.J., Freire S., Halkia S., Julea A.M., Kemper T., Soille P. and V. Syrris, Operating procedure for the production of the Global Human Settlement Layer from Landsat data of the epochs 1975, 1990, 2000, and 2014. Publications Office of the European Union, EUR 27741 EN, 2016. doi: 10.2788/253582.
