# Supplementary material for: The evolution of built-up areas in Ghana since 1975
Source: PLoS One. 2021 May 21;16(5):e0250847. doi: 10.1371/journal.pone.0250847 (PMC8139467; doi:10.1371/journal.pone.0250847)
Supplement: Attachment — Submitted filename: SubmissionPLOS_ONE2_withresponses..pdf [file pone.0250847.s002.pdf]

Stanford, September 30, 2020

Dear Sir/Madam,

In response to an invitation to submit from Diego Rybski, I am submitting my paper with Forhad Shilpi entitled “The Evolution of Built-up Areas in Ghana since 1975” for possible publication in Cities as Complex Systems PLOS ONE Collection. The paper is not currently under consideration by any other journal.

An earlier version of the paper was submitted in June of this year and was assigned the number PONE-D-20-17761. It received a ‘reject-and-resubmit’ verdict in July 2020. This resubmission is accompanied by a detailed response to the comments received from the original editor and referees. Thanks for your consideration.

Sincerely,

Marcel Fafchamps

Senior Fellow

Center for Democracy, Development, and the Rule of Law

Response to the Editor
[original text in roman font, response in italics]

PONE-D-20-17761
The Evolution of Built-up Areas in Ghana since 1975
PLOS ONE

Thank you for submitting your manuscript to PLOS ONE. After careful consideration, we have decided that your manuscript does not meet our criteria for publication and must therefore be rejected.

If however, you are able to address the reviewers' concerns through revisions to your manuscript, we may be willing to consider a resubmission of your work. The revised manuscript should be submitted as a new submission to PLOS ONE and be accompanied by a cover letter that refers to the original submission number and details how the revisions have been completed. I am sorry that we cannot be more positive on this occasion, but hope that you understand the reasons for this decision.

Thank you for allowing us to revise and resubmit our paper as a new submission. We attach below a detailed response to the changes we have brought to the paper in response to the referees' comments. We believe we have adequately addressed them all, to the extent allowed by the data. Thanks for these comments. We feel that the paper is much improved as a result.

Response to Reviewer 1

The paper is purely a description of so-called the evolution of built-up areas in Ghana. I cannot see any theoretical/conceptual/methodological/practical contribution from this paper.

Thanks for pointing this out. We have written a completely new conceptual framework section, for which we have created several new Figures that illustrate how our analysis relates to the framework. These Figures also clarify the methodological contribution made by the paper. In addition, we have moved the conceptual framework to Section 2, brought it up directly into the introduction, and linked our findings to it in the conclusion. We believe these important changes make the contribution of the paper much clearer. Thanks for nudging us in that direction.

The introduction does not tell us why we have to understand this research.

We have taken this point to heart and entirely rewritten the introduction to better explain to the reader the motivation behind our project and the reasons for the choices we made.

There is no critical review of the existing literature. Readers are left with no idea about the knowledge gap into which this paper is intended to fill. There exists no dialogue between this paper and the existing studies.

Good point, related to the discussion above. Partly this is due to the fact that much of the current literature focuses on big cities, while we focus on all human settlements, including small rural ones. But it is fair to say that we could have done a better job of linking our work to the rest of the literature. This has now been corrected. We have also added quite a few references to the current literature, helping the reader better realize how our paper fits in the present literature.

The conclusion of the paper reads like the outcome of a consultation report.

Ouch! We did not want to oversell our results. But the referee is right to point out that we can do better. The conclusion has been entirely rewritten to read more like an academic paper. For the record, this paper was never part of a consulting project. It was a research project from the start.

Response to Reviewer 2

The authors used high resolution satellite data to study evolution of human settlement in Ghana over a 40 years period. The authors have done a lot of work to analyze the expansion of human settlement in Ghana from many aspects, and have obtained several valuable and meaningful findings. However, the quality of this manuscript is not enough for publication, there are still some areas that need to be supplemented and improved. Therefore, I recommend a major revision for this manuscript. Please see suggestions below which are not mandatory.

1. I suggest that the authors re-write the abstract because it has a high repetition rate with the conclusion. Add 1-2 sentences to clarify the importance and significance of this study and let readers know why you did this research. In addition, it is necessary to briefly introduce the method of this study.

The abstract has been corrected to eliminate duplication with the conclusion, better motivate the study, and introduce the methodology. The conclusion itself has been entirely rewritten to better explain how our findings can be interpreted in the light of the newly rewritten conceptual section, which now plays more prominently in the introduction and conclusion.

2. The authors point out that the purpose of this paper is to document how the spatial distribution of buildings has evolved. The authors need to re-explain the main purpose and significance of this study.

This comment complements those of the first referee, and confirms that we did not do a good enough job motivating the study, and explaining its purpose and significance. The introduction has been fully rewritten to address these shortcomings, and to build a better link between the paper and the existing literature.

3. The major scientific contribution of the work is not clear. Although the authors point out that this paper contributes to the literature on urbanization and regional development, I think the contribution and innovation of the paper are not accurate and specific.

This weakness too is related to the comments from Referee #1. We needed to clarify from the start what our conceptual framework was -- it was discussed in section 3 but it was not brought enough to the fore in the rest of the paper. This has now been corrected. We have also moved the conceptual section to the second position, before introducing the data, and the section itself has been completely rewritten. Several Figures have been added that clarify the methodological contribution made by the paper. In addition, we have introduced the conceptual framework directly into the introduction, and linked our findings to it in the conclusion. We now do a much better job a clarifying what the anticipated and actual contributions of the paper are, and how they relate to the existing literature.

4. In the section of Introduction, the authors have shown too much research process, results or conclusions, I think it is not necessary, or the authors can make a brief overview. In my opinion,

the authors should pay more attention to introducing research background, literature review and scientific contribution in this section. Let readers know the purpose and significance of this study.

These are good suggestions. We have rewritten the introduction taking your recommendations into consideration. Thanks for the guidance.

5. In the section of Data, I think the authors should detail the sources and processing of the data in this study. It is necessary to add a section describing the methods. More details about the study area are also necessary, adding figures to show the data and study area.

Good point. We have expanded the main text to include more information about the way the human settlement data was generated, drawing from published data documentation. In addition to notes at the bottom of the Tables and Figures, an online appendix has been included that details the way the data were manipulated to generate the various graphs and tables in the paper. We also provide a bit more contextual information about Ghana in the paper, and include a short online Appendix for the reader unfamiliar with the country.

6. In the section of Empirical results, the authors both showed the results and discussed them. Therefore, I think the title of this section should be changed to "Empirical results and discussion".

Done.

7. In this current version of the manuscript, I did not see the Appendix Figures A1 and A2.

The two omitted figures have been added. Sorry about that.

8. In the maps at the end of the paper, the maps 1e, 1d, 2e, and 2f are not marked with the order and name.

Sorry about that. This omission has been corrected.
