# Supplementary material for: The evolution of built-up areas in Ghana since 1975
Source: PLoS One. 2021 May 21;16(5):e0250847. doi: 10.1371/journal.pone.0250847 (PMC8139467; doi:10.1371/journal.pone.0250847)
Supplement: Attachment — Submitted filename: PONE-D-20-30587_reviewer.pdf [file pone.0250847.s003.pdf]

PLOS ONE

The Evolution of Built-up Areas in Ghana since 1975

--Manuscript Draft--

**Manuscript Number:**	PONE-D-20-30587
**Article Type:**	Research Article
**Full Title:**	The Evolution of Built-up Areas in Ghana since 1975
**Short Title:**	Built-up areas in Ghana
**Corresponding Author:**	Marcel Fafchamps<br>Stanford University<br>Stanford, UNITED STATES
**Keywords:**	Human settlement pattern; transition matrix; roads; agglomeration; urbanization forecasts
**Abstract:**	We use high resolution satellite data on the proportion of buildings in a 250x250 meter cell to study the evolution of human settlement in Ghana over a 40 year period. We find a strong increase in built-up area over time, mostly concentrated in the vicinity of roads, and also directly on the coast. We find strong evidence of agglomeration effects both in the static sense – buildup in one cell predicts buildup in a nearby cell – and in a dynamic sense – buildup in a cell predicts buildup in that cell later on and an increase in buildup in nearby cells. These effects are strongest over a 3 to 15 Km radius, which corresponds to a natural hinterland for a population without mechanized transportation. We find no evidence that human settlements are spaced more or less equally either over the landscape or along roads. This suggests that arable land is not yet fully utilized, allowing rural settlements to be separated by areas of unfarmed land. By fitting a transition matrix to the data, we predict a sharp increase in the proportion of the country that is densely built-up by the middle and the end of the century, but no increase in the proportion of partially built-up locations. This means that, based on current trends, we do not expect urban growth in Ghana to take the form of urban sprawl.
**Order of Authors:**	Marcel Fafchamps
	Forhad Shilpi
**Additional Information:**	
**Question**	**Response**
**Financial Disclosure**<br><br>Enter a financial disclosure statement that describes the sources of funding for the work included in this submission. Review the submission guidelines for detailed requirements. View published research articles from *PLOS ONE* for specific examples.<br><br>This statement is required for submission and **will appear in the published article** if the submission is accepted. Please make sure it is accurate.	The research was funded by the World Bank, of which one of the authors, Forhad Shilpi, is an employee. All views expressed in the paper are those of authors and should not be attributed to the World Bank or its affiliates. The World Bank played an important role in helping us put the data together from various public sources. The World Bank had no role in the analysis, decision to publish, or preparation of the manuscript -- other than the direct involvement of one of its employees, Forhad Shilpi, in the writing of the manuscript.

Powered by Editorial Manager® and ProduXion Manager® from Aries Systems Corporation

**Unfunded studies**<br>Enter: *The author(s) received no specific funding for this work.*<br><br>**Funded studies**<br>Enter a statement with the following details:<br>• Initials of the authors who received each award<br>• Grant numbers awarded to each author<br>• The full name of each funder<br>• URL of each funder website<br>• Did the sponsors or funders play any role in the study design, data collection and analysis, decision to publish, or preparation of the manuscript?<br>• **NO** - Include this sentence at the end of your statement: *The funders had no role in study design, data collection and analysis, decision to publish, or preparation of the manuscript.*<br>• **YES** - Specify the role(s) played.<br><br>* typeset	
**Competing Interests**<br><br>Use the instructions below to enter a competing interest statement for this submission. On behalf of all authors, disclose any competing interests that could be perceived to bias this work—acknowledging all financial support and any other relevant financial or non-financial competing interests.<br><br>This statement **will appear in the published article** if the submission is accepted. Please make sure it is accurate. View published research articles from *PLOS ONE* for specific examples.	No author has any competing interest.

Powered by Editorial Manager® and ProduXion Manager® from Aries Systems Corporation

**NO authors have competing interests**<br><br>Enter: *The authors have declared that no competing interests exist.*<br><br>**Authors with competing interests**<br><br>Enter competing interest details beginning with this statement:<br><br>*I have read the journal's policy and the authors of this manuscript have the following competing interests: [insert competing interests here]*<br><br>* typeset	
**Ethics Statement**<br><br>Enter an ethics statement for this submission. This statement is required if the study involved:<br><br>• Human participants<br>• Human specimens or tissue<br>• Vertebrate animals or cephalopods<br>• Vertebrate embryos or tissues<br>• Field research<br><br>Write "N/A" if the submission does not require an ethics statement.<br><br>General guidance is provided below. Consult the submission guidelines for detailed instructions. **Make sure that all information entered here is included in the Methods section of the manuscript.**	n.a.

**Format for specific study types**<br><br>**Human Subject Research (involving human participants and/or tissue)**<br>• Give the name of the institutional review board or ethics committee that approved the study<br>• Include the approval number and/or a statement indicating approval of this research<br>• Indicate the form of consent obtained (written/oral) or the reason that consent was not obtained (e.g. the data were analyzed anonymously)<br><br>**Animal Research (involving vertebrate animals, embryos or tissues)**<br>• Provide the name of the Institutional Animal Care and Use Committee (IACUC) or other relevant ethics board that reviewed the study protocol, and indicate whether they approved this research or granted a formal waiver of ethical approval<br>• Include an approval number if one was obtained<br>• If the study involved *non-human primates*, add *additional details* about animal welfare and steps taken to ameliorate suffering<br>• If anesthesia, euthanasia, or any kind of animal sacrifice is part of the study, include briefly which substances and/or methods were applied<br><br>**Field Research**<br><br>Include the following details if this study involves the collection of plant, animal, or other materials from a natural setting:<br>• Field permit number<br>• Name of the institution or relevant body that granted permission	
**Data Availability**<br><br>Authors are required to make all data underlying the findings described fully available, without restriction, and from the time of publication. PLOS allows rare exceptions to address legal and ethical concerns. See the PLOS Data Policy and FAQ for detailed information.	Yes - all data are fully available without restriction

A Data Availability Statement describing where the data can be found is required at submission. Your answers to this question constitute the Data Availability Statement and **will be published in the article**, if accepted.<br><br>**Important:** Stating 'data available on request from the author' is not sufficient. If your data are only available upon request, select 'No' for the first question and explain your exceptional situation in the text box.<br><br>Do the authors confirm that all data underlying the findings described in their manuscript are fully available without restriction?	
**Describe where the data may be found in full sentences. If you are copying our sample text, replace any instances of XXX with the appropriate details.**<br><br>• If the data are **held or will be held in a public repository**, include URLs, accession numbers or DOIs. If this information will only be available after acceptance, indicate this by ticking the box below. For example: *All XXX files are available from the XXX database (accession number(s) XXX, XXX.).*<br>• If the data are all contained **within the manuscript and/or Supporting Information files**, enter the following: *All relevant data are within the manuscript and its Supporting Information files.*<br>• If neither of these applies but you are able to provide **details of access elsewhere**, with or without limitations, please do so. For example:<br><br>*Data cannot be shared publicly because of [XXX]. Data are available from the XXX Institutional Data Access / Ethics Committee (contact via XXX) for researchers who meet the criteria for access to confidential data.*<br><br>*The data underlying the results presented in the study are available from (include the name of the third party*	The core data used in the paper is publicly available and comes directly from the Global Human Settlement Layer (GHSL) data that have been put together by the European Space agency. We combine these data with information on agro-ecological zones and soil quality from the Global Agro-Ecological Zones (GAEZ) 2012 data of the FAO, and with road information from GIS maps of Ghana put together by the World Bank. We will make our combined dataset publicly available at the time of publication.

and contact information or URL).<br>• This text is appropriate if the data are owned by a third party and authors do not have permission to share the data.<br><br>* typeset	
Additional data availability information:	Tick here if the URLs/accession numbers/DOIs will be available only after acceptance of the manuscript for publication so that we can ensure their inclusion before publication.

Click here to access/download;Manuscript;GhanaGeoV8WithAllTablesandFig

The Evolution of Built-up Areas in Ghana since 1975*

Marcel Fafchamps† Forhad Shilpi‡

September 2020

Abstract

We use high resolution satellite data on the proportion of buildings in a 250x250 meter cell to study the evolution of human settlement in Ghana over a 40 year period. We find a strong increase in built-up area over time, mostly concentrated in the vicinity of roads, and also directly on the coast. We find strong evidence of agglomeration effects both in the static sense – buildup in one cell predicts buildup in a nearby cell – and in a dynamic sense – buildup in a cell predicts buildup in that cell later on and an increase in buildup in nearby cells. These effects are strongest over a 3 to 15 Km radius, which corresponds to a natural hinterland for a population without mechanized transportation. We find no evidence that human settlements are spaced more or less equally either over the landscape or along roads. This suggests that arable land is not yet fully utilized, allowing rural settlements to be separated by areas of unfarmed land. By fitting a transition matrix to the data, we predict a sharp increase in the proportion of the country that is densely built-up by the middle and the end of the century, but no increase in the proportion of partially built-up locations. This means that, based on current trends, we do not expect urban growth in Ghana to take the form of urban sprawl.

Keywords: Human settlement pattern; transition matrix; roads; agglomeration; urbanization forecasts

*We thank the editor and two anonymous referees for their useful comments. We would like to acknowledge Varnitha Kurli for excellent research assistance in extracting all relevant GIS data used in the study. Our sincere thanks to Brian Blankespoor for his help with the GIS data. We are grateful to the World Bank for funding this research. All views expressed in the paper are those of authors and should not be attributed to the Bank or its affiliates..

†Stanford University, NBER, and IZA. Address: 616 Jan Stanford Way, Stanford CA 94305. Email: fafchamp@stanford.edu

‡The World Bank, 1818 H Street, Washington DC, USA.

1 Introduction

The world population has been growing rapidly. This is particularly true in Africa where the population has multiplied manyfold since the beginning of the 20th century, and is predicted to reach four billion by the end of the 20th (e.g., UN 2013).[1] Increases in the African population have spurred urbanization in general, and the emergence of mega-cities of several million inhabitants in many -- if not most -- African countries (e.g., Seto et al. 2011, Lall, Henderson and Venables 2017). How this rapid expansion of human settlements unfolds over geographical space is of paramount importance for development (e.g., Duranton 2015) and has likely deep consequences on inequality and poverty (e.g., UNDP 2017). While these structural changes create formidable challenges for policy makers (e.g., Bryan et al. 2019), relatively little is known -- beyond the above general observations -- on the process by which urbanization is unfolding in the continent.

Much attention has recently been devoted to the growth of cities in lower and middle income countries (e.g., Henderson and Turner 2020 for a review). Most of the recent work in urban economics in lower and middle income countries has focused on large cities (e.g., Bryan et al. 2019), their form (e.g., Salop 1979; Hariri 2019; Zhang et al. 2020), and the role of urban transit (Tsivanidis 2020; Balboni et al. 2020) and land tenure regulation (Gechter and Tsivanidis 2020, Collin 2020) in shaping the urban space. This paper focuses instead on the growth of low density areas, which is an equally important question for policy. Indeed, if partially built-up areas grow disproportionately over time – e.g., because of urban sprawl or agricultural intensification – this creates different needs for public infrastructure investment and market development than if population growth concentrates in a few high density areas.[2]

This paper looks into the question using detailed data on building density across space and time. We take advantage of the recently available Global Human Settlement Layer (GHSL) data from the Joint Centre of the European Commission, that uses machine learning tools to predict the presence of buildings from satellite imagery (e.g., Pesaresi et al. 2016). We focus on Ghana, a West African country that has experienced steady growth in population and GDP, without incurring any

[1] Vollset et al. (2020) make lower predictions overall, but still predict a large population increase in Africa during the first half of the century.

[2] E.g., for roads, schools, and health facilities, as well as for public utilities such as water, electricity, sewers, garbage collection, and the like. It would also dictate a different spatial and size distribution of bank branches and retail outlets.

civil conflict since independence in 1957. Appendix C provides detailed background information about Ghana, its history, and its geography. At the heart of this study is a panel dataset of 3.8 million rectangle-shaped cells covering the entire territory of Ghana and observed in 1975, 1990, 2000, and 2014. For each of these cells, the GHSL dataset has an ML prediction of the proportion of that cell's area that is covered by buildings. We regard the existence of buildings as indicating the presence of people and economic activity – although we do not have direct evidence on either. Given that we have precise geocoordinates for each cell, we can combine this large dataset with information on roads, humidity, soil types, and proximity to borders and bodies of water. Appendix D provides additional information about the data.

With these data we investigate an issue understudied in LDCs: the spatial evolution of population density, proxied by buildup. From the literature we know that partial buildup – a special feature of our data – arises mainly in two settings: at the periphery of towns and cities; and in farmed areas. The urban periphery attracts a mix of peri-urban farming and urban commuters in search of cheaper housing (e.g., Henderson 1985, Fujita et al. 1999). The result is a low density buildup in the vicinity of densely built urban centers. As towns grow, low density areas can grow around them, resulting in urban sprawl; or the city can crowd out its hinterland. Regarding farmland, we expect population growth to lead to agricultural intensification (e.g., Boserup 1965) and to the emergence of a non-farm sector and rural market towns (e.g., Jedwab and Moradi 2016, Jedwab et al. 2017). We therefore expect rural towns to have a small densely built center where non-farm production concentrates, surrounded by low density buildup made of a mix of farms and residences for non-farm producers. Given that rural towns are small, rural densification should show up as an increase in partially built-up areas. How fast low density areas grow relative to urban centers is an issue of interest to city planners.

Our second focus is the spacing of human settlements. In 1826, von Thunen proposed a theory of rural market towns serving a surrounding agricultural hinterland. This theory was refined by Christaller (1933) and Isard (1956) into a model of regularly spaced towns of varying size.[3] It assumes that rural hubs are located close to each other, otherwise the spacing regularity need not arise. We look for regularity in the spacing of human settlements, either on a plane, or along roads, at different points in time. Regular spacing of human settlements simplifies regional planning by

[3]Salop (1979) offered a similar model on a road network.

forecasting future settlement patterns and facilitating infractruture positioning.

We start by documenting the evolution of the distribution of buidup density. Over our study period, the average buildup density went from 0.4% in 1975 to 0.8% in 1990, 1.1% in 2000 and 1.8% in 2014. The proportion of cells without buildings dropped from 98.5% to 93.8% while the number of built-up cells went up. Among cells with at least one building, we note an increase from 7% to 12% in the proportion of densely (i.e., more than 90%) built-up cells. In contrast, the distribution of partially built-up cells remains constant. This suggests that urban growth in Ghana has not taken the form of urban sprawl. Rather, towns and cities tend to crowd out their hinterland, probably because of commuting constraints. We also note a large increase in small and medium urban settlements: urbanization is not just in a few large cities.

Next, we compute transition matrices and show that once a cell gets built up, it seldom reverts to an unbuilt state. If Ghana were to continue evolving as it did between 1975 and 2014, there would still be 91.9% unbuilt cells by 2055. But the share of the densely built-up areas would increase by 130% -- admittedly from a very low base. We expect much growth in secondary towns and rural market hubs. We look at differences across agro-ecological zones. We initially find more buildings in semi-humid areas and few in waterlogged or steep areas. But over the 40 years of the study period, many of the originally avoided areas experience fast buildup growth, possibly because investments such as the Volta dam and the reduction of onchocercosis made these locations more attractive for production.

New construction between 1975 and 2014 happened over a small fraction of the country's total area. Roads attract a disproportionate share of the increase in build-up: only 14.24% of cells are located at most 1.5km from a road in 1976, yet they attract approximately 62.6% of buildings. The same is true in 1990 when 66.7% of all built-up land is located within 250 meters from a road. Buildup forecasts based on fitted transition matrices confirms that proximity to roads is associated with a much larger increase in future buildup. Since new roads locate near already built-up areas, we investigate whether subsequent construction locates near newly built roads. We find that proximity to 1976 and 1986 roads predicts later buildup – but 1986 roads are a stronger predictor. We cannot tell whether construction near a new road is a net addition, or whether it displaces construction from elsewhere. More research is needed on the causal effect of roads using quasi-natural experiments such as those used by Banerjee et al. (2012) and Faber (2014).

The last part of the analysis uses spatial covariograms to measure agglomeration effects and and examine the regularity in the spacing of settlements. We find that buildup in a cell predicts more buildup in cells located up to 3 to 5 km. For cells located near roads, there is still some evidence of more buildup up to 40-50 km away. For cells not near a road, the effect fades away faster, consistent with the idea that transport costs constrain the size of the town's hinterland. Over time, however, the covariogram increases, capturing the general increase in buildup. But, over our study period, we find no evidence that densely built areas locate at regular intervals. One possible interpretation is that agricultural density is not yet dense enough for rural settlements to be crunched near each other.

This paper contributes to the literature on urbanization and regional development (e.g., Seto et al. 2011, Henderson and Turner 2020). Data on built-up area at a highly disaggregated level has recently become available (Pesaresi et al. 2016). This has encouraged a newly emerged literature that seeks to make use of this information to explore important economic realities. Bellefon et al. (2018), for instance, use buildup proportion as a way of identifying the edges of cities, arguing that partial buildup identifies sub-urban areas. We adopt a similar philosophy in the sense that we regard building concentration as an indication of a dense human settlement in an urban setting.

Recent literature has shown a substantial impact of transport infrastructure and technology on the geographical concentration of economic activities (Thompson, 2000; Michaels, 2008; Banerjee et al., 2012; Donaldson, 2014; Faber, 2014; Bird and Straub, 2014). Much of this evidence is at a fairly aggregate level, e.g., a city or town. We show that a similar pattern exists for smaller settlements of a radius of a few hundred meters. Henderson et al. (2018) show that, in countries that developed early, cities located in agricultural regions because of high transport costs, a pattern of agglomeration that survived after transport costs fell. In a similar vein, Jedwab (2014) documents how, in Ghana and Côte d'Ivoire, cocoa production spawned many small towns and how these towns survived the gradual westward shift of cocoa plantations that took place over the last century. Our results confirm that buildup is highly persistent: once a building appears in a cell, it is very rare for this cell not to also have a building in subsequent years, even though we cannot say if it is the same building. Moreover, places that are already settled tend to attract additional buildings, meaning that they become a focal point for population growth. The same can be said of roads, in the sense that proximity to a road in 1976 or 1986 is a strong predictor of human settlement in 2014, even

when that road has since disappeared.

An intellectually elegant literature, spearheaded by von Thunen in 1826 (1966), argues that human economic activity self-organizes over space into equally spaced settlements. Similar ideas have been applied to the distance from which workers are willing to commute, and to study the geographical coverage of shopping malls and grocery stores. Some evidence has been provided that towns and cities are more or less equally spaced from each other – e.g., Jedwab and Moradi (2016) and Jedwab et al. (2017) for African towns along railway lines; and Faber (2014) for Chinese cities along newly built freeways. In both of these cases, however, planning by a government or colonial authority seems to have been influential. We find no such evidence in Ghana over the study period, something we interpret within the theory as implying that the full utilitization of arable land has not been reached yet. A related strand of literature has tested whether the size distribution of cities follows Zipf's Law (Gabaix 1999, Arshad et al. 2018). We contribute to this literature by showing that at the very smallest level – i.e., partial buildup of a small geographical cell, Zipf's Law does not apply: the log of the buildup proportion of cells is far from forming a linear relationship with the log of the rank of that buildup proportion. We see this as extending earlier work to the lowest level of geographical disaggregation.

We start in Section 2 by introducing our conceptual framework. Section 3 explains how the data are constructed. Empirical results are presented in Section 4.

2 Conceptual framework

The literature on regional and urban economics is, in one way or another, about where people and economic activity locate and how this changes over time. In this paper we revisit this issue using a newly available, highly disaggregated dataset on buildup in a West African country. While this dataset has considerable breadth in terms of coverage and geographical detail, it also lacks many variables that have received attention in the literature – such as population, jobs, sectors of activity, production, and infrastructure. This forces us to adopt a reduced-form approach that emphasizes breadth instead of depth.

To begin, we regard the presence of buildings in a cell as indicating the presence of population and, hence, of economic activity nearby. The denser buildup is in a cell, the more people live in

that cell, deriving a livelihood from work in the vicinity. This starting point in unproblematic given the level of generality of our analysis. Our data contain lots of cells that are only partially built. Hence, in order to make full use of our data, we need to put together a conceptual framework on partially built-up cells that is inspired from the literature while remaining sufficiently sparse to match the limited depth of our data. This conceptual framework also must include all cells – not just metropolitian areas, which often are the sole focus of spatially disaggregated empirical analysis (e.g., Hornbeck and Keniston 2017; Ambrus et al. 2020; Gechter and Tsivanidis 2020; He 2020; Harari 2020; Zhang et al. 2020).

The lowest level of building density – e.g., a lone building surrounded by unbuilt cells – is probably a farm. Ghana is a large agricultural country with a relatively undifferentiated geography, but a strong rainfall gradient running from North (semi-arid) to South (humid).[4] Since humid areas can support a higher population density than dry ones, we expect a higher frequency of farms in the South. More generally, agricultural potential varies with agro-ecological features and so does agricultural population density. What is less clear is whether, in denser areas, farms cluster in villages – in which case buildup is concentrated in a few cells – or whether they disperse across the land – in which case there are many cells with a single homestead on them. Which pattern best describes Ghana's rural population is not entirely clear – some factors militate in favor of clustering (e.g., for defense purposes during the slave trade); others in favor of dispersion (e.g., long fallows). Data on partially built-up cells provides an easy way of characterizing this distribution.

Population growth has also fostered a process of agricultural expansion whereby land previously unfarmed gets newly settled. This process is common to much of sub-Saharan Africa, and was still underway during our study period. We therefore expect that, over time, new farms tspring up in previously empty cells. What is unclear is whether these new farms form new clusters, or whether they spread out more or less evenly over the landscape as time passes. The answer to this question is of obvious interest to policy markets interested in rural infrastructure and agricultural marketing: it is a lot easier to provide agricultural and public services to a population that is relatively concentrated. To cast light on this question, we examine how the distribution of partially built-up cells evolves over time: do we observe a gradual dispersion of buildings across the land, or a gradual concentration. We also investigate what is the long-term trend in terms of building (and

[4] More details are provided in Appendix C.

thus population) dispersion across the country. This is done by constructing the transition matrix of built-up areas and using it to predict buildup distributions by the middle and the end of this century. This research question is related to well-studied processes in developed countries, such as rural flight and and its relative converse, urban sprawl (e.g., Dennis et al. 2019).

A closely related issue is the location of rural market towns. As rural population increases, a demand for specialized services arises (e.g., retail shops, hair salon, bicycle repair) that favors the emergence of rural towns (Boserup 1965). Rural settlements can also be jumpstarted by investments in agricultural infrastructure and production (e.g., Dell and Olken 2020) or mining (e.g., Fafchamps et al. 2017). Sub-Saharan Africa – as in other LDCs – has experienced a rapid rise in the growth of rural non-farm enterprises, often in the form of small shops and artisans (e.g., Jedwab 2014). This process of rural small-scale urbanization – and the accompanying distribution of human settlement across space – was already the focus of von Thunen's (1826/1966) work, and has since attracted the attention of numerous others (e.g., Christaller 1933, Isard 1956, Henderson 1985, and Fujita et al. 1999). The common thread running through this literature is the concept of rural hinterland: each market town serves a surrounding area, the diameter of which depends on transportation costs. For instance, if farmers will only walk two hours to sell their crops and purchase retail goods, the diameter of the hinterland of a market town is two hours walking time. In this case, we expect the landscape to be characterized by small market towns surrounded by farms scattered over a radius of a few kilometers at most. We show in Appendix Figure A1 what this pattern of settlement may look like, together a spatial covariogram that shows, each pairwise distance between cells, the proportion of pairs made of two built-up cells. We note that, at close distances, there is an excess of built-up pairs. This captures the strength of agglomeration forces: buildings are located in and around small local hubs. At longer distances, there is no systematic pattern because settlements are located a random distances from each other.

If population density is low, rural hubs can locate at will – e.g., in locations that have good access to arable land and drinking water. When density reaches a certain level, however, the hinterland of these rural hubs start to overlap. This generate competition between them at the margin, e.g., to attract farmers for selling their crops and buying consumer items. Provided that rural consumers enjoy a diverse offer of goods and services in a single location, rural market towns – like shopping malls – cannot locate immediatedly next to each other. This results in a regular

spacing of rural towns, either on a plane (in which case hinterlands form a honeycomb shape with market towns at the center – Isard 1956) or along a road (e.g., Salop 1979; Faber 2014) or railway line (e.g., Donaldson 2014). The hinterland concept is illustrated in Appendix Figure A2 where we show, on the left, a simulated example with an equal-spacing distribution of rural hubs. There is some limited evidence that rural towns are fairly equally spaced in Africa (e.g., Jedwab and Moradi 2016) but this theory has never been tested at the level of an entire country. Regular spacing of small market towns on a plane would show up in a spatial covariogram as a spike at regular intervals, as shown in the right-hand graph of Appendix Figure A2. Regular spacing along roads would show the same signature when limiting the attention to cells along roads. We test these ideas in the empirical part of the paper. We do not expect this regular spacing to hold when rural population density is too low.

In our data, small rural market towns may occupy one or two cells at most. What of larger settlements, what distribution of buildup cells can we expect to observe? While there is a lot of literature on the growth of large cities (see Seto et al. 2011 for a review), less attention has been devoted to the growth of urban settlements of small and middle sizes, especially on the African continent. In particular, little is known regarding the expansion of partially built-up cells during rapid urbanization: do towns and cities grow as densely packed entities; or do they spawn a large urban hinterland of peru-urban agriculture (e.g., Jacobs 1969) or urban commuters (e.g., Barbour et al. 2019)? To articulate these ideas formally, we develop a toy model of cities as concentric circles: at the middle of the city, all cells are fully built according to our data definition (i.e., cells contains buildings, roads, sidewalks, parking lots, and the like). This center is then surrounded by a sequence of donuts of different size characterized by a decreasing density of buildup (see Appendix Figure A3, top left). We think of falling buildup density as resulting from a varying mix of sub-urban residences for commuters – which can be densely packed – and those of farmers who provide urban consumers with vegetables, fruits, and dairy products and need land to farm. As distance to the center grows, fewer commuters move in and more land is left for farming. This is obviously a simplication, but it is sufficient for our purpose.

How do the donuts change as the town grows? Regarding farmers, we can imagine that demand for peri-urban crops increases more or less proportionally with population and thus with the size of the town center. Similarly, we can expect the number of town workers to increase more or

less proportionally with town size – and thus the number of aspiring sub-urban commuters. This implies that if the town center – made of fully built-up cells – expands by a factor of k, so does the area covered by the partially built-up donuts that surrounds it. To see this, let C denote the fully built-up area at the town center. We have $C = \pi r^2$ where r is the radius of the center. Let R denote the outer radius of a partially built donut that surrounds the town center.[5] Imagine that the area of the center grows by a factor k. By how much must R increase for the area of the donut to also increase by k? The answer is k.[6] This means that, in this world, the proportion of partially built-up land around towns and cities increases at the same rate as the fully built-up land. This prediction is testable with the data we have. If we combine this prediction with the rise of partially built-up cells due to agriculture and small market towns that we discussed earlier, we expect the fraction of total land that is partially built-up across the country to increase at the same rate or faster than the fraction of fully built-up cells. One specific version of this prediction is Zipf Law applied to the size of human settlements. The size distribution of cities is said to follow Zipf Law if we observe a linear relationship between the log of city size and the log of the rank of a city in terms of size (e.g., Gabaix 1999). Applied to buildup in cells, Zipf Law would imply a linear relationship between the log of buildup proportion and the log of the rank of the cell in terms of buildup. If Zipf Law were to hold for buildup, it would also imply that frequency distribution of buildup across cells would remain constant as urbanization takes place – which in our toy model could only arise if city growth implies proportional growth in the size of the hinterland where partially built-up cells are located. The reverse is, however, not true: the frequency distribution of buildup could be time-invariant but not follow a power law. We test this hypothesis as well.

So far we have assumed that the area travelled by commuters does not matter. As the town grows, however, suburbs move further out, and sub-urban commuters must travel a longer distance. Presumably, as R keeps increasing, the length and duration of the commute increase in roughly the same proportion (if not more, e.g., due to increased congestion in the center). Without investment in rapid transit infrastructure for commuters, this is likely to reduce the appeal of suburban living, convincing some would-be commuters to shift to high density residential areas closer to town center.

[5] In the discussion here we imagine a single donut, but the same reasoning applies to multiple donuts of varying density.

[6] The area of the donut is $\pi R^2 - \pi r^2$. We want the ratio of the donut area to the center area to remain constant, i.e., we want $\frac{\pi R^2 - \pi r^2}{\pi r^2}$ = constant when r is set to kr. It is immediately clear that this requires that R be multiplied by the same k.

In this case, the radius of the outer donut grows less and the radius of the center grows more for a given population increase. In the extreme case where R represents an absolute limit on commutable distance, urban growth is characterized by an increase in r while R remains unchanged. The town grows up to the point at which $r = R$ and the entire area is fully built-up, at which point it stops: the city has reached the limit set by the available transport infrastructure and technology (e.g., Henderson 1985, Fujita et al. 1999, Tsivanidis 2020). A similar prediction holds even if some rapid transit infrastructure is introduced, e.g., in large cities, but it is insufficient to allow R to increase as the same speed as r: the city still densifies, even if at a slower rate. Thes cases are illustrated in Appendix Figure A3. Here the prediction is that the *ratio* of partially built-up urban cells over fully built-up cells falls as the proportion of fully built-up cells grows. This is because cities densify as they grow (i.e., the ratio of $\frac{R}{r}$ falls as r increases). This is illustrated in Appendix Figure A4, which shows that simulated frequency distribution of partially and fully built-up cells as a town grows as shown in Figure A3. Initially there is a large fraction of partially built-up cells, especially low buildup density cells. As the town grows, the proportion of these cells falls while the proportion of fully built-up cells rises. But the proportion of built-up cells of intermediate density does not change much. In contrast, if a city's hinterland expands proportionally to the city center, the frequency distribution of partially and fully built-up cells remains unchanged. This means that the growth of sparsely built-up cells would rise at par with fully built-up cells. In the paper, we look at the long-term evolution of the frequency distribution of partially and fully built-up cell to throw light on whether towns and cities crowd out their hinterland as they grow, or whether their hinterland grows with them. We nonetheless recognize that a fall in the ratio of sparsely built-up cells to fully built-up cells due to crowding-out may be partially offset by growth in the number of rural market towns. Here too, however, the same logic applies for individual towns if commuting time is a constraint: as small settlements grow, they absorb their hinterland, and the fraction of partially built-up land falls threre as well. Only the creation of a sufficiently large number of *new* low-density villages and rural market towns can compensate this evolution. Whether this took place in Ghana over our study period – and is likely to continue over the foreseable future – is thus an empirical question.

The visual representation of the concept of hinterland used so far is spherical, which essentially assumes that travel time is the same in all directions. This may be a reasonable assumption for

travel by foot or bicycle on the relatively flat rural terrain that characterizes much of Ghana. It does, however, fail to recognize the role played by roads in facilitating transport and commuting. Conceptually, roads can be thought of as reshaping space to make certain locations closer to each other (in terms of travel time or cost) than they were without it. We mention this idea earlier when discussing workers commuting from sub-urban areas: roads – and transit systems more generally – help shape cities (e.g., Tsivanidis 2020). Roads can also shape the locations of rural market towns and even farmers themselves, e.g., because they allow inter-city trade (e.g., Redding 2020). We take this into account in our empirical analysis by examining the long-term effect that past roads may have on the location of human settlements, while simultaneously examining agglomeration effects.

3 Data

Our main variable of interest y_{it} measures the proportion of a cell i in year t that is covered by buildings, as inferred from a high-definition satellite image of Ghana. This variable comes from the Global Human Settlement Layer (GHSL) data that have been put together by the Joint Centre of the European Commission. A Mollweide projection was used to divide the earth's surface into square cells of approximately 250 by 250 meters, which is rather small for an analysis of built-up area. The proportion of buildings on each cell was predicted using a machine learning algorithm trained on observations of actual buildings made on the ground. Pesaresi et al. (2016) describes the machine learning algorithm in detail. This algorithm predicts buildup in squares of 38x38 meters. These predictions are then aggregated using GIS into 250x250m cells, thereby generating an accurate prediction of the proportion of the cell that has buildings on it. We overlay the GHSL data on a GIS map of Ghana and extract the cells that lie within the country's borders.[7] A little over 3.8 million cells are needed to cover the entire area of Ghana.[8] Additional information about the data is provided in Appendix D.

The GHSL dataset provides information on builtup cells for four separate years: 1975, 1990, 2000, and 2014. Cells for each of these years have been carefully aligned using detailed GPS coordinates so as to form a panel dataset for y_{it}.[9] Variable y_{it} takes values between 0 and 1, with

[7]All data used in this paper are extracted using codes written in R.

[8]In practice, in our data each cell measures approximately 225 meters wide by 275 meters high (approximately 675 by 825 feet or 6.2 hectares) due to Ghana's location close to the equator.

[9]Aligning satellite photographs with each other and over time was achieved projecting each photograph using the

0 meaning no building detected in the cell, and 1 meaning the cell is entirely occupied by buildings – i.e., as in a city center.[10] As shown in Table 1, most cells (98.5%) in 1975 are predicted to be unbuilt, which is hardly surprising given that Ghana at the time only had a national population density of 43.9 per square km. Builtup area grows over time as population increases to 119.4 per sqkm in 2014 and Ghana becomes more urbanized (urban population share increasing from 30% in 1975 to 53% in 2014). In 2014, 93.8% of cells were unbuilt.

For each cell i in year t we construct two measures of buildup in the cells surrounding i. The first of these measures is denoted as y_{it}^{n1} and is the average built-up area in the cells immediately adjacent to i. Since the data is divided into a square grid, each cell on that grid in principle has 8 immediate neighbors, which constitute the set n_1. We thus have:

$$y_{it}^{n1} \equiv \frac{1}{n_1} \sum_{j \in n_1} y_{jt} \tag{1}$$

where by an abuse of notation n_1 represents both the set of immediate neighbors and their number. Together with y_{it}, this variable captures the proportion of built-up area in an approximate cell 675 meters wide by 825 meters high (approximately 2025 by 2475 feet).[11] We also construct a variable y_{it}^{n2} that captures the average buildup in cells immediately adjacent to y_{it}^{n1}. There are in principle 16 cells in set n_2, except in the vicinity of borders and large bodies of water where that number gets truncated. The formula for y_{it}^{n2} is that same as equation (1) but with n_1 replaced by n_2. Together, y_{it}, y_{it}^{n1} and y_{it}^{n2} cover an area 1125 meters wide and 1375 meters wide, roughly 1.55 square kilometers. These are generated by using spatial lag functions in R.

We combine data on built-up area with information on agro-ecological zones to capture the wide variety of landscapes and agricultural potential that characterize Ghana.[12] The data for agro-ecological zones are downloaded from the GAEZ-Global Agro-Ecological Zones data portal that was developed by the Land and Water Division of the Natural Resources Management and Environment Department of FAO. The resolution for GAEZ dataset is 10 km. We overlay the 250

same Mollweide projection.

[10]More precisely, $y_{it} = 1$ when all its area is covered by impervious surfaces, such as roofs, sidewalks, streets, and courtyards.

[11]Cells located on the border or along the sea or large body of water do not have a full complement of 8 adjacent cells located wholly or partly within Ghana. For these cells, the immediate neighborhood is limited to those cells included in the dataset and the average is adjusted accordingly.

[12]The agro-ecological information is missing for some of the cells located at the edges of the map.

by 250 meter grid of the GHSL dataset over the GAEZ dataset to extract soil types for eahc cell. We divide this information into two variables, which do not vary over time. The first of these variables characterizes the humidity of a particular region, i.e., whether moist/dry, semi-humid, or humid. In Ghana as in the rest of West Africa, the rains follows a monsoon pattern and rainfall generally decreases along a North-South gradient, with the North being drier and the South being more humid. There are exceptions to this general pattern, however – the immediate coastal area, for instance, tends to be drier than inland. In the data, humid soils are associated with the entire valley of the Volta river and with the forested region North of Takoradi.

The second agro-ecological variable we construct captures soil quality – going from poor to moderately good to good. There are two additional categories: hydromorphic soil, which is characterized by excess moisture which tends to suppress aerobic factors in soil-building; and soils unsuitable for buildings or agriculture, such as steep soils or soils covered by standing water at least part of the year – these soils require special investments (e.g., terassing, drainage) in order to become usable. Given that Ghana is relatively flat with no mountains and a relatively homogenous physical landscape, we abstract from altitude: whatever variation in altitude there may be, it translates into variation in rainfall and soil quality and is already captured.

To this data we add longitude and latitude information of cells from the GHSL dataset expressed in decimal degrees, which we normalize to have mean zero and unit variance. A higher value of longitude means more to the West; a higher value of latitude means more to the North. Population density in Ghana is in general higher in the humid South than in the drier North, largely due to the higher agricultural potential and better soils in the South. But there is a lot of local variation, something we aim to capture with our set of agro-ecological and geographical variables.

We also have information about the presence on a cell of a land border, sea border, or large body of water from the GIS shape files of Ghana. We drop from the y_{it} database of all the cells outside Ghana, as well as those that are entirely covered by water, since they cannot be built on. This includes lake Volta, a large artificial lake that covers 8,502 square kilometers within Ghana. Some cells are partially outside Ghana, or partially covered in water or by the sea, something we capture with dummies. We also construct a dummy equal to one if there is a large body of water on any of the eight adjacent cells n_1, and another one for the next 16 adjacent cells n_2. This is done to investigate whether proximity to a lake or lagoon attracts human settlement, e.g., if the

lake is use to fish or transport goods. The same thing is done for proximity to the sea and to a land border, for similar reasons.

The last piece of information that we integrate into our analysis is road data. For 1976 and 1986, we digitized road maps from the Ghana population censuses. These maps are then used to extract information on the presence and/or absence of roads in GHSL cells. These roads are classified into four categories, from 1 to 4.[13] Class 1 roads are the highest quality, and class 4 roads the poorest. Typically, a class 1 road is paved and hence usable all year round. In contrast, a class 4 is a dirt track and many of these roads are seasonal. Class 2 and 3 occupy an intermediate position. Road availability in 1976 is of particular interest for our purpose because these roads predate our period of analysis and thus cannot be construed as caused by buildings constructed after 1976. The same can be said of 1986 roads regarding new buildings constructed afterwards.[14] Using the digitized maps, for each cell we construct a dummy r_{kit} equal to 1 if there is a road of class k in cell i in year $t = 1976$ or $t = 1986$ and 0 otherwise. This is done by overlaying road maps on the GHSL cells defined earlier. Next we calculate the proportion of immediately adjacent cells that contain a road of each particular class, which we denote r_{kit}^{n1}. Mirroring what we did for built-up area, we also calculate the proportion of roads in the n_2 neighborhood of i, which we denote r_{kit}^{n2}.

4 Empirical results and discussion

4.1 Descriptive statistics

We start by showing in Tables 1 to 3 key descriptive statistics for our variables of interest. From Table 1 we see that proportion of empty cells ($y_{it} = 0$) fell 98.5% to 93.8% between 1975 and 2014. Averages are essentially identical for y_{it}, y_{it}^{n1} and y_{it}^{n2}, which is hardly surprising since they average essentially the same cells.[15] The proportion of fully built-up cells ($y_{it} = 1$) among built-up cells increased a lot between 1975 and 1990 – from 2% to 6.6%, but does not show any trend after that. We revisit this point more in detail below. We check whether Zipf's Law applies to built-up area

[13]There is also a class 5 which is labeled 'unknown road'. Our of an abundance of caution, we ignored these roads in our analysis.

[14]We also have data on roads in 2008, divided into primary, secondary, and tertiary roads. Unfortunately, these categories reflect administrative definitions, and they do not overlap with those used in 1976 and 1986, which focus on the physical qualities of the road. Since we are interested in predicting settlement patterns starting in 1975, this data is less relevant and is ignored in most of the analysis.

[15]A small difference arises due to the fact that y_{it}^{n1} and y_{it}^{n2} are normalized by the size of the neighborhood. This gives a bit more weight to small neighborhoods in the averages of y_{it}^{n1} and y_{it}^{n2} relative to y_{it}.

at this micro level. To do this, we sort partially built-up cells by the proportion of cell area that is built up – from largest to lowest. We then plot the log of the rank to the log of the built-up area. Here cells with a built-up proportion close to 1 represent 'cities' while those with a built-up proportion close to 0 represent the smallest possible town. Results are shown in Appendix Figures B1 (in levels) and B2 (in logs). We find absolutely no evidence of a Zipf's Law for partial buildup similar to what has been discussed for cities (e.g., Gabaix 1999, Ashrad et al. 2018).

In Maps 1a to 1d, we illustrate what the data looks like for a randomly chosen location in Ghana – the rural town of Yeji. Buildup is color coded, with white meaning no evidence of any building in the cell and black meaning high density buildup – i.e., 90% and above. Intermediate shades of blue and grey correspond to different levels of partial buildup. We see that in 1975 there is very little evidence of buildup – most of Map 1a is empty. But the small settlement of Yeji can be seen in the upper middle of the map. Over time, this initial settlement grows and expands geographically. It also spawns small satellite settlements at more or less random but fairly equally spaced intervals. New settlements also appear farther away from the initial settlement, often in the proximity of a road. But the initial settlement remains the most densely built-up area of the map throughout the 40 years period covered by the data, suggesting some form of preferential attachment/first-mover advantage. Maps 1e and 1f show the same general area as a Google Earth photograph and Google map print, respectively. It is immediately clear that the data contain a wealth of detail about buildup that is hard to see at this level of magnification, but it comes to view under magnification. The picture also reveals the presence of the Volta lake nearby. Some of the roads shown in Map1f correspond to 1975 roads. However, one road running to the west from Yeji seems no longer in use, even though its outline is still visible from the air. Similar map sequences for other parts of Ghana show a similar evolution, at least visually (Maps 2a to f show the evolution of buildup in the coastal town of Takoradi). The rest of the paper is devoted to analyzing these patterns in a formal way.

Road information is presented in Table 2. For 1976 and 1986, the road information we have captures road quality, divided into four categories, plus a small 'quality unknown' category. At that time, most of the roads included in this list are unpaved. According to these data, the proportion of cells with at least one road was 3% in 1976 and 3.4% in 1986. The 2008 data use a more stringent road definition, according to which 1.8% of cells had at least one road. The 2014 data includes two

new categories: highways, which at the time only include on short stretch of motorway/freeway between Accra and a nearby town; and local roads. The meaning of the other three categories also seems to have changed between the two years, precluding a direct comparison. In the analysis we rely heavily on the 1976 data because it captures the predetermined road infrastructure at the beginning of our study period. Averages for road availability in the 8 n_1 and 16 n_2 neighboring cells are not shown to save space but, by construction, they are identical to the cell averages reported here.

In Table 3 we summarize our main time-invariant variables. Longitude and latitude are presented in decimal degrees. They are used to measure the distance between cells in the subsequent analysis. A tiny fraction of cells are on the border with a neighboring country or on the seashore, but 2.7% are partially covered by a water body such as Lake Volta. The predominant soil quality is either poor or moderate, a situation that typifies much of Sub-Saharan Africa and reflects its tropical nature: heavy rainfall combined with high evapotranspiration means considerable leaching of nutrients from the soil. Only 7.4% of soils are categorized as good. There is a small fraction of cells with hydroponic soils, which means they are located in river estuaries or flood plains. Some of these soils can be made fertile, but often at some cost in terms of infrastructure and equipment. Some 5% of cells have land unsuited for traditional agriculture either because they are waterlogged or too steep. Cultivating these soils requires investment in drainage or terracing. In terms of humidity, most of Ghana's land is classified as sub-humid, which some areas being either drier or more humid. Rainfall typically follows a North-South gradient in western Africa, with more rains along the coast than in the North.[16] There are exceptions, however: coastal areas of Ghana tend to receive less rainfall than those located more inland. The humidity of the soil also depends on its texture. In Ghana, soils in the valley of the Volta river are classified as humid as they tend to retain moisture better.

In Table 4 we break down y_{it} by agro-ecological zone and proximity to borders and water. We find strong initial differences in buildup density across zones with, in 1975, more buildings (and hence a higher population density) in semi-humid areas and in areas with humid or hydromorphic soils. Over the study period, however, we observe dramatic changes in relative buildup densities:

[16] An ordered probit regression of the climate variable on latitude returns a strongly negative coefficient with a pseudo-$R^2 = 0.28$.

cells with initially the lowest buildup densities – i.e., areas with soils that humid, hydromorphic, or waterlogged or steep – experience the fastest growth, possibly suggesting that investments were successful in making these soils or locations more productive. Reduction in onchocercosis may have also attracted settlement in these areas.

Table 5 presents a similar breakdown of built-up areas by the presence or not of a road in the cell. Not surprisingly, we find a strong difference in buildup density between cells with and without a road. This difference falls over time however: comparing cells with and without a road in 1976, the difference falls from 8.5 more buildup in 1975 to 6.3 times in 2014. We also note that the new road definitions adopted in the 2014 data match heavily built-up areas much more closely than before, suggesting that this new road nomenclature puts more emphasis on residential streets (e.g., Gonzalez-Navarro and Quintana-Domeque (2016).) and less on dirt roads serving rural communities.

In Table 6 we show buildup proportions in 'edge cells', that is, cells that overlap with a land border or a water body. We see that the presence of a water body is in general associated with a much lower buildup density. This may be because the largest inner body of water is a dam lake and hence probably has a water level that varies over time depending on annual rainfall: people don't want to live right next to it. Land borders also have a lower density than no-edge cells, while cells near the sea have a much higher buildup density, probably reflecting the presence of towns and cities along the coast, but also the fishing communities that launch their fishing boats directly from the beach. We also observe a much faster buildup over time in cells near inner water bodies, probably reflecting the fact that Lake Volta was only completed in 1965 and it probably took time for people to relocate along its permanent shore.

4.2 Buildup frequency distribution

Next, we look in more detail at the frequency distribution of buildup for cells that are neither empty of buildings or fully occupied by them. Figure 1 shows this distribution for the four data years we have. It is immediately clear that the frequency distribution of partially built cells remains constant over time, even if the proportion of unbuilt cells falls while the proportion of fully built-up cells increases. We then break down this distribution by cells that are located near built-up areas and cells that are not, i.e, by whether $y_{it}^{n2} > 0$ or $= 0$. Unsurprisingly perhaps, we find that isolated

built-up cells only have a small fraction of built-up area: half of them are less than 4% built up, which is equivalent to having at most a $50m^2$ (550 square feet) structure on them; three quarters of them have structures covering at most $83m^2$ or 915 square feet. In contrast, half of built-up cells in the vicinity of other built-up cells have structures covering $106m^2$ or more – with a fairly even distribution of y_{it} across the entire spectrum of built-up proportion, a finding in line with the idea that these cells correspond to the edges of towns and cities. This pattern suggests that population growth at the aggregate level is not associated with a densification of all areas simultaneously. Rather, what we observe is a fall in the proportion of empty cells, an increase in the proportion of densely built cells, and a more or less constant distribution of partially built cells.

To investigate this issue further, we construct the transition matrix M of y_{it} between 1975 and 2014 and, taking the full distribution of y_{it} in 2014 as starting point, we multiply it a couple times by M to produce forecasts of the distribution of built-up area in Ghana around the years 2055 and 2095. These forecasts implicitly assume a constant rate of aggregate population growth, or more precisely the same rate of increase in aggregate buildup across the country. We also calculate the ergodic distribution corresponding to the transition matrix. This distribution is not intended to be a forecast; rather it represents the long-term distributional trend implied by M.

The forecasts are shown in Figure 2, together with the actual built-up proportions in 1975 and 2014. The histogram confirms that the evolution of built-up proportions in Ghana is characterized by a transfer from unbuilt to fully built, with partially built-up cells serving a transitory role. Over the foreseeable future, the calculations forecast 89.5% unbuilt cells circa 2050 and 85% unbuilt cells circa 2100. At the same time, the proportion of very densely built areas (90% built-up or more) will go from 0.9% in 2014 to 3% in 2053 and 6.2% in 2092. We predict no equivalent increase in partially built cells. In other words, the additional population will not spread itself evenly on the land. Rather, it will congregate in cities. Assuming that the historical rate of population growth continues, this represents more than a tripling of dense urban areas in about 40 years, and a multiplication by 7 by the end of the century. Very similar forecast are obtained if we construct the transition matrix using only 1976 and 1986, or 2000 and 2014: the matrices are very similar, and the forecasts as well. Although our forecasts apply to buildings only, they imply a dramatic change in the distribution of population across the country, with an increased concentration in towns and cities without much of a densification of rural areas. To illustrate the magnitude of

these changes, an equivalent increase in the population of Accra would imply an increase from 2.2 million in 2014 to 7.3 million people by 2053 and more than 15 million by the end of the century. These are not small changes.

4.3 Roads

In Figures 3a and 3b, we repeat the exercise separately for cells that were close to a road in 1976 and those that were not. We define being close to a road as having at least one of the five categories of roads documented in 1976 either in the cell itself, or in its neighborhood n_1 or n_2. With this definition, 14.24% of cells at most 1.5km from a road in 1976. The year 1976 is chosen because it is close to the starting point of our data. Hence the transition matrices that are constructed on that basis can be seen as predictions based on road conditions existing in 1976. Results show a stark contrast between cells near road or not. Cells near a 1976 road were already less likely to be unbuilt in 1975: 93.7% vs. 99.3% for cells not near a road. This gap increased to 81% vs. 96% in 2014 and is predicted to grow to 61% vs. 90% by the end of the century. Furthermore, the distribution of built-up areas also varies greatly depending on the presence of a road in 1976. In 1975, only 0.05% of cells not near a road had a value of y_{it} in excess of 90%, compared to 0.73% of those near a road. By 2014 the difference was 0.46% vs. 3.71%. By the end of the century, the forecasted difference is 4% vs. 19%. Put differently, by the end of the century nearly 19% of the area within 1.5km of a 1976 road will be nearly fully built up. Of course new roads will be built that can affect that, a point we revisit below. But the main message here is that the early presence of a road is predicted to have long lasting effects on urbanization patterns. Figures 3a and 3b also show that while proximity to a road affects the probability of subsequent buildup, it does not markedly change the distribution of partially built-up cells: while the levels are different between the two figures, the shape of the distribution is similar. This is true even though each transition matrix is estimated from two distinct sets of observations.

Next, we expand on the above analysis to investigate the effect of adding or removing a nearby road. We use the data from 1976 and 1986 since they are comparable. We define four categories: cells not close to a road in either 1976 or 1986; cells close to a 1976 road that no longer existed in 1986; cells close to a road that did not exist in 1976 but was present in 1986; and cells close to a road that existed both in 1976 and 1986. Abandoning a road may signal that the road was no

longer economically useful which, if true, should predict less growth and less subsequent buildup. In contrast roads that remained in existence across both years should be correlated with a pre-existing and persistent economic usefulness. Hence, we expect them to be most associated with economic activity and thus most likely to attract population and buildup. Roads that were added in 1986 should occupy an intermediate position: they were not initially deemed useful but were added later, perhaps when sufficient funds became available to add less essential roads. For the analysis, we construct four transition matrices from the 1975-2014 data on these four categories of cells. We then predict buildup in 2053 and 2092 as before. To save space, we present in Figures 4a and 4b only the results for unbuilt and fully built cells, respectively. Indeed, as noted earlier, the actual and forecasted distributions of partially build cells are fairly stable across time. We see that, as anticipated, cells near a road are predicted to receive a lot more buildup over time. The buildup forecast is particular strong for locations that had a road in 1986. Whether or not they also had a road in 1976 matters less, contrary to what we expected. Cells near an abandoned road do less well, but still better than cells with no road in either years.

4.4 Agglomeration

Having discussed roads, we now turn to agglomeration effects. We first examine the extent of correlation between built-up levels y_{it}, y_{it}^{n1} and y_{it}^{n2}. To this effect we estimate a simple OLS regression of the form:

$$y_{it} = \beta_0 + \beta_1 y_{it}^{n1} + \beta_2 y_{it}^{n2} + u_{it} \tag{2}$$

It goes without saying that the estimated coefficients should not be given a causal interpretation; they simply document the spatial correlation patterns in buildup. The results, shown in Table 7, are very stable across data years: we note a strong contemporaneous correlation between y_{it} and y_{it}^{n1} but, conditional on y_{it}^{n1}, the correlation with y_{it}^{n2} is negative. Put differently, if we observe high buildup in n_1 and n_2, we would predict a lower buildup y_{it} than if we only observed high buildup in n_1 alone. This potentially suggest that buildups on either side of n_1 are substitutes: if happens in n_2, it is less likely to occur in cell i.

Next, we investigate whether cells in a built-up neighborhood subsequently experience more

buildup. We start by estimating a simple OLS regression of the form:

$$y_{it} = \beta_0 + \beta_1 y_{it-s} + \beta_2 y^{n1}_{it-s} + \beta_3 y^{n2}_{it-s} + u_{it} \tag{3}$$

where s is a time lag. Results are presented in Table 8 for the six separate regressions. Unsurprisingly, they show a lot of persistence in buildup: the estimated coefficient β_1 is large in all regressions, indicating that past buildup is a strong predictor of future buildup. This predictive power, however, falls with the time gap s: the more distant in the past is y_{it-s}, the smaller the value of β_1, and the smaller is the precision of the prediction, i.e., the R^2. We also notice that, when the time interval s is short, the additional predictive power of y^{n1}_{it} and y^{n2}_{it} is relatively small. As the time interval increases, the coefficients – and thus the predictive power – of y^{n1}_{it} and y^{n2}_{it} increases and, unlike in Table 7, it is positive for both. This is consistent with a process by which much of the diffusion of buildup to neighboring cells occurs over a relatively short time interval of less than 10 to 15 years. Once this diffusion has begun, own past buildup takes over the predictive power of buildup in neighboring cells. This finding is suggestive of some form of hysteresis: once a cell has started to build up, it tends to remain that way, irrespective of neighboring effects.

We confirm this interpretation by estimating a non-parametric version of regression (3) in which the three regressors are replaced by dummies for each of the deciles of the relevant regressors. We also include a vector X_i of time-invariant cell characteristics as controls. These include soil quality and soil humidity categories; latitude, longitude, their cross-product; and dummies for the presence of a body of water, a land border, or a sea border. The estimated regression has the form:

$$y_{it} = \beta_0 + \sum_{k=1}^{10} \beta_1^k y^k_{it-s} + \sum_{k=1}^{10} \beta_2^k y^{kn1}_{it-s} + \sum_{k=1}^{10} \beta_3^k y^{kn2}_{it-s} + \theta X_i + u_{it} \tag{4}$$

where y^k_{it-1} is a dummy equal to 1 for the k'th decile of y_{it-s}, and y^{kn1}_{it-s} and y^{kn2}_{it-s} are the same set decile dummies for y^{n1}_{it} and y^{n2}_{it}. We only estimate regression (4) for the shortest lag s, i.e., using 1975 for 1990, 1990 for 2000, and 2000 for 2014. Coefficient estimates for the buildup variables of interest are presented in graphical form in Figure 5. Results by and large confirm earlier findings from Table 8. They also provide some evidence of a stronger lag effect for higher deciles of y_{it-s}, suggesting more persistence for high buildup. Interestingly, we see that the lagged effect of buildup

in neighboring cells tends to taper off at high buildup values, suggesting some form of substitution effect perhaps.

We next expand the model to include dummies for different road categories. The estimated regression has the form:

$$y_{it} = \beta_0 + \sum_{k=1}^{10} \beta_1^k y_{it-s}^k + \sum_{k=1}^{10} \beta_2^k y_{it-s}^{kn1} + \sum_{k=1}^{10} \beta_3^k y_{it-s}^{kn2} + \gamma_1 r_{it-s} + \gamma_2 r_{it-s}^{n_1} + \gamma_3 r_{it-s}^{n_2} + \theta X_i + u_{it} \quad (5)$$

where: r_{it-s} is a vector of road availability dummies, one for each road type, at time $t-s$ in cell i; $r_{it-s}^{n_1}$ is a vector of average availability of different types of roads in neighborhood n_1, and $r_{it-s}^{n_2}$ is the same for neighborhood n_2. We focus our attention on outcome years 1990 and 2000 because for each of them we have comparable road data approximately 14 years earlier, i.e., in 1976 and 1986. Road data for 2008 and 2014 are not comparable to either of these or to each other. Coefficient estimates for γ_1, γ_2 and γ_3 are presented in Figure 6, for each of the four road categories and each of the two outcome years, 1990 and 2000. Coefficient γ_1 gives the predicted % point increase in buildup if there is a road in the cell 14 years before. Coefficients γ_2 and γ_3 give the predicted % point increase in buildup if neighborhoods N1 and N2, respectively, go from no cell with a road to all cells with a road. We note a lot of similarity between the two regressions in terms of relative magnitude of the coefficients: the presence of a class 1 road in the cell or nearby predicts more buildup 14 years later. For the other three categories, the presence of a road in the cell predicts slightly higher buildup later on, but the presence of a road in the more distant neighborhood N2 has a consistently negative effect on subsequent buildup predictions. A similar negative coefficient is observed in some regressions for neighborhood N1 as well. These findings are again consistent with the existence of a substitution effect in buildup: if the road is in a neighboring cell but not in my cell, my cell is likely to be built-up a decade and a half later – an outcome we would expect if local inhabitants prefer to locate immediately near these smaller roads. When we estimate regression (5) with 2014 buildup y_{it} and the 2008 road data, we find that all the road coefficients but one are positive. The only exception is for the presence of a primary road in the cell itself, which by 2014 may have too much traffic to be an attractive location. The 2008 road data is completely different from the earlier data, however, with very little correlation between the two, thereby making comparisons problematic. The 2014 road data is strongly correlated with buildup

in that year, but since it is contemporary to our last data year, it cannot be regarded as a useful predictor.

We also estimate a different version of regression (5) setting $t-s=1975$ for lagged buildup and $t-s=1976$ for the road data. By regressing each of the subsequent data years 1990, 2000 and 2014 on these initial buildup and road conditions, we examine the delayed predictive effect of buildup and roads over time. Figure 7 summarizes the buildup coefficients in a way similar to Figure 4, the main difference being that now all regressions use the same 1975 buildup data as regressors. The Figure shows clearly that, over time, the predictive effect of past buildup changes. For buildup in neighborhoods N1 and N2, we see a systematic increase in buildup predictions as time passes. This is consistent with some form or preferential attachment – the presence of buildings in the past attracts new buildings later – possibly due to agglomeration externalities. Past buildup in the cell itself varies depending on the original buildup intensity: for heavily built-up cells in 1975, the own-cell predictive effect falls over time. In contrast, cells with less buildup in 1975 see their buildup predictions increase over time. The fall in predictions for heavily built-up cells may suggest some form of congestion, unless it is compensated by buildup in neighboring cells, which makes it harder to move to a nearby cell.

Figure 8 does the same thing for coefficient estimates for γ_1, γ_2 and γ_3. The interpretation of the coefficients is similar to that in Figure 6, except that we are now comparing the evolution of buildup predictions over time, using roads in 1976 as predictor. We immediately see that the predicted effect of class 1 roads increases massively over time both for the presence of a road in the cell, as well as for road density in neighboring cells. The other three road categories produced similar results: we observe an increasing positive effect of roads in the cell and in the immediate neighborhood N1 on buildup predictions, while road presence in neighborhood N2 has a negative effect that is initially stronger and falls later on. This is consistent with an initial substitution effect that starts to reverse itself as population density increases and moving to a neighboring cell to get near the road becomes harder.

4.5 Spatial covariogram

So far we have been looking at agglomeration effects at fairly close range – i.e., up to 1.5km at most. We found mostly evidence of agglomeration effects, except occasionally when we uncovered patterns

suggestive of spatial substitution effects. We now explore this possibility further by broadening the range of distances we consider so as to produce a spatial covariogram of cell buildup. To recall, the spatial covariogram of a spatial variable z plots the average correlation between observations of z for various distances between them. This is in principle achieved by calculating the distance between all possible pairs of observations and then by computing, for each relevant distance interval, the sample correlations between y pairs located within that distance interval from each other. The concept is similar to autocovariogram in time series analysis, except that it is calculated over distances which, in data like ours, are not limited to integer values.

To do this effectively, we have to overcome the challenge of dimensionality. With nearly 4 million cells, calculating all pairwise distances would generate around 16 trillion observations – a number that far exceeds the computing capacity at our disposal. To implement our plan, we therefore need to resort to a few tricks. The first trick is to focus on the spatial covariogram and simplify the calculation of covariance by focusing on a dichotomous variable $z_{it} \equiv Pr(y_{it} > 0)$. This means that the sample equivalent of the spatial covariance $E[z_{it}z_{jt}]$ at distance d is simply the proportion of cell pairs ij at distance d that are both 1. Our objective is thus to plot a sample estimate of $E[z_{it}z_{jt}]$ at various distances d for Ghana. To this effect, we need to construct a representative sample of cell pairs and calculate the proportion of pairs that are both built-up among all sampled pairs.

To achieve this, we divide Ghana into a number of tiles small enough that we are able to construct all the cell pairs in that tile. This is done by creating a grid of 856 equal size tiles that covers all of Ghana.[17] This implies that we are not using all the data at our disposal: pairs of observations that belong to different tiles are not used to construct the covariogram. Since tile boundaries are chosen at regular intervals based on latitude and longitude, they are orthogonal to the phenomenon we study. Hence the resulting sample of cell pairs should be representative. Each of these tiles is an 18×18 km square (approximately 26 km in diagonal). It comprises around 5,200 cells and approximately 13.5 million unique cell pairs. We then divide cell pairs into approximately 260 distance bins of 100 meters each. The number of cell pairs in each bin varies with the shape

[17] In practice, this is achieved as follows. We first identify the extrema of latitude and longitude in Ghana. We then divide the North-South difference into 33 equal intervals. We do the same for the East-West difference. This creates 1,089 blocks, 856 of which cover at least part of Ghana; the rest fall in neighboring countries and are ignored here. We also drop a handful of tiles with a number of cells too small to compute a covariogram.

of the tile and the presence of borders and water bodies. We then count, for each distance bin b, the total number of pairs $N_b = \sum_{ij \in b} C_{ijt}$ in that bin – where $C_{ijt} = 1$ for cell pair ij and the summation is taken over all the cell pairs in bin b. We also count, for each year t and each bin b in each tile m the number of pairs with two built-up cells, that is, two cells i and j in b for which $z_{it} = 1$ and $z_{jt} = 1$. This enables us to calculate the share S_{btm} of cell pairs in tile m and in distance bin b that are both built up in year t as:

$$S_{btm} = \frac{\sum_{ij \in b,m} z_{jt} z_{jt}}{N_{bm}}$$

Since this is done separately for each tile m, it means that the values of S_{btm} can be computed separately tile by tile before assembling them together to cover all of Ghana.

Because country borders and bodies of water are irregular,[18] the size and shape of each tile varies somewhat and so does the number of cell pairs in each bin of each tile. This means that to reconstruct the country-level covariogram, we need to weight each S_{btm} by the number of cell pairs that it represents. To achieve this, we simply regress S_{btm} for a given year on distance bin dummies:

$$S_{btm} = \sum_b \alpha_b D_{bm} + u_{btm}$$

and weight each observation S_{btm} by the number of observations N_{bm} in bin b of tile m. We then plot, for each year t the predicted values $\hat{S}_b$ (they only vary by distance bin b) and the prediction standard errors obtained from the above regression.[19] This process yields a spatial covariogram averaged across all tiles and weighted by the number of cell pairs in each bin of that tile.

The results are shown in Figure 9 for all four years combined. We see that, since the proportion of built-up cells increases over time, the proportion of built-up cell pairs not surprisingly increases as well. Otherwise the spatial pattern is similar across all years: cells located close to a built-up cell are more likely to be built up as well: $E[z_{it} z_{jt}]$ falls with distance. The correlation is particularly strong at short distances of up to 3 km, but the pattern is present across the entire distance distribution and, given how tight the confidence intervals are, differences in spatial correlation by distance are significant up to distances of at least 20km. Beyond 20 km, the number of observations in

[18]Plus a small variation in the length of one degree of longitude from North to South Ghana.

[19]We only show confidence intervals for 1975 and 2014, to keep the graph uncluttered. As is immediately apparent from the graph, confidence intervals are extremely tight.

distance bins N_{bm} drops rapidly, confidence intervals increase in size, and the covariogram becomes unreliable, a point we revisit below.

We repeat the exercise separately for cell pairs both located near a road in 1976, and other cell pairs. Proximity to a road is defined as having a road in the cell itself or in neighborhoods n_1 or n_2 – i.e., as having r_{it_0}, $r_{it_0}^{n_1}$, or $r_{it_0}^{n_2} > 0$ for $t_0 = 1976$. The resulting spatial covariogram is shown in Figure 10 for all four years of data we have. The general pattern is similar to that observed for all cell pairs, except that the proportion of built-up cells is higher near roads, and thus there are more built-up cell *pairs* near roads. We also observe the sharply higher spatial correlation at close distances of around 2-3 km. But the spatial correlation in buildup falls less fast near roads than away from roads. This is consistent with our earlier finding that proximity to a road predicts higher buildup independently from proximity to a pre-existing agglomeration, and this effect is distinct from the fact that settlements often are near roads to start with. The spatial covariogram for cell pairs that are not close to roads is very similar to that reported in Figure 9 and is not shown here to save space.

Contrary to what is predicted by the von Thunen (1966) model subsequently expanded by Chistaller (1933) and Isard (1956), we find no strong evidence of a 'bump' in the covariogram at any specific distance. If farm buildings tended to be spaced equally so as to locate in the middle of their fields, we would expect a spike in the covariogram at the relevant distance. For instance, if each farming households occupies, say, 20 Ha of cultivated land, fallows and pastures, we would expect farms to be, on average, 500 meters from each other.[20] This would more or less correspond to one farm per cell, and thus a flat spatial covariogram between farm buildings. We also would not expect to find a buildup concentration along roads – which is not what we find. In contrast, if farmers live in villages of, say, 100 households needing each 20 Ha of combined cultivated land, fallows and pastures, each village would require an area of 2,000 Ha, which would imply a spacing of villages each 5 km. If this were the case, we would expect a spike in the spatial covariogram at 5km: the presence of village buildings at distance 0 would predict village buildings 5 km away. We do not find evidence consistent with this conjecture – or any equal spacing of villages up to 20 km. We do however find evidence of agglomerations covering an area of up to 3 km in radius

[20] 20 Ha is 200,000 m^2, an area that is contained in a circle with a 250 meter radius. Two such circles would be, on average, roughly 500 meters from each other.

– 6 km or 4 miles in diameter. These localities may be larger than simple villages, and they may be located further apart than 20 km. Could there be, therefore, spikes in the spatial covariogram at distances above 20km – something that is possibly suggested by the appearance of a spike at distances slightly above 20km in Figure 10?

To investigate this possibility, we recalculate the spacial covariogram for distances up to 75 km. To do this, we divided Ghana into 86 tiles of roughly 60 × 60 km, with a diagonal distance of over 76km. Because the proportion of built-up cells is low, we are still able to form all built-up cell pairs and to compute $\sum_{ij \in b,m} z_{jt} z_{jt}$ for all but the 3 most densely built-up tiles.[21] We cannot compute N_{bm} directly, however, due to computer power requirements. We therefore must proceed differently. The total number T_m of unique cell pairs in each tile is immediately obtainable: it is $T_m = N_m \times (N_m - 1)/2$, where N_m is the number of cells in tile m. What we need is an estimate of how these $N_m \times (N_m - 1)/2$ are divided across distance tiles. Indeed, because tiles vary in shape, the distribution of pairs across distance bins varies across tiles. To construct an estimate of the frequency distribution of cell pairs into distance bins for each tile, we select in each tile a large but manageable random sample of cells. We form all cell pairs among them and calculate the number of pairs in each distance bin R_{bm}. Let $R_m = \sum_b R_{bm}$ be the total number of cell pairs in the random sample for tile m. Since we did not use all cells in the tile, $R_m < T_m$. The frequency distribution of distance bins in each tile can nonetheless be approximated as:

$$N_{bm} \simeq R_{bm} \times \frac{T_m}{R_m}$$

where T_m/R_m is used to rescale the distribution of weights to match the full size of the tile.

Results comparable to Figures 9 and 10 are shown in Figures 11 and 12, respectively. We see that the reported patterns are very similar to those reported earlier. The fall in buildup correlation that was observed at short distances in Figure 9 is confirmed in Figure 11, and it is seem to continue then flatten our around about 50 km of distance. A similar pattern is shown in Figure 12 for cell pairs located near a road, except that the fall is steeper at short distances and the slope is steeper for longer. The curve also flattens our at around 50 km distance. We also observe that the bump

[21]The three omitted tiles each contain a large city and thus a large proportion of built-up cells. This means that the number of built-up cell pairs is too large for us to compute on our computer. These three cities, however, contain little usable information to test the idea that small towns are more or less equally spaced. If anything, including them would probably reduce the likelihood of finding a spike.

at distances above 20 km in Figure 10 is not a robust result and is probably an artifact of the small number of tiles and cell pairs with observations at those distances. Using larger tiles solves that problem. These results confirm our earlier findings, namely that, across Ghana, human settlements (as proxied by buildings) are not evenly spaced, as theories inspired by von Thunen would predict. This is true across cells in general, but also along roads. We do, however, find strong evidence of agglomeration at short distances, as well as evidence of weak agglomeration forces operating up 50 km away – in the sense that being less than 50 km from a built-up location predict a slightly higher probability of buildup. The figures also confirm that buildings tend to concentrate along roads and near each other.

In the last figure, we perform a similar exercise using small tiles as unit of observation. Since nearly all these tiles include at least one building, we define buildup as being in the top decile in term of buildup, averaged over the whole tile. We then form all tile pairs and conduct the same analysis as in Figures 9 and 11. Distance between tiles is calculated from the tile's centroid.[22] As we have much fewer observations, we need not put tile pairs into distance bins: we can work directly on the full sample of pairs. Given that nearly all tiles contain at least one road, we ignore that dimension here. Figure 13 shows the result, with distance on the horizontal axis and, on the vertical axis, the share of tile pairs that are both in the top buildup decile. By construction, the horizontal axis is truncated at distances less than 18 km, which is the size of a tile itself. The four curves show, for each of the data year, the predictions from a fractional polynomial regression of the share of top decile pairs on distance. If buildup were distributed randomly across space, we should observe a flat curve. This is not what we see: as in Figures 9 to 12 we find evidence of agglomeration, which is strongest at distances up to around 60 km, which is the range of distances covered in Figures 11 and 12. Correlation in high buildup falls after that, but only gradually – suggesting that agglomeration continues to have an effect on buildup even at long distances.

5 Conclusions

Developing countries across the world experienced strong population growth during the last four decades. With population growth, urbanization also picked up pace with half of the developing

[22]For our purpose, the centroid of a tile is defined as the point with the average longitude and the average latitude of the tile.

world's population now living in urban areas. The accelerated urbanization has received considerable attention in the Africa region where urbanization has not been associated as strongly with economic growth as observed historically in developed countries. Less is however known about how human settlements expanded geographically along with population growth and increased urbanization. In this paper, we make use of high resolution satellite data on built-up areas within micro-pixels to study the evolution of human settlements in Ghana.

Our analysis is based on a simple conceptual framework organized around the von Thunen idea of hinterland, which is the basin of economic attraction of a town or city. Given the existence of agglomeration effects in non-farm services, this idea predicts rural market towns or settlements surrounded by farms scattered within an area that can be reached from the hub. Since, over our period of study, most rural dwellers would simply walk or cycle to the nearest market, the radius of this area would not exceed 5 to 15 Km. This is pretty much what we find in the data. If all arable land is used, the hinterland of these rural hubs abut each other, which results in a regular distribution of rural hubs. We find no evidence of this in the data. This is consistent with the observation that, in Ghana over the study period, not all arable land was cultivated, thereby allowing for unfarmed areas of varying size to separate rural settlements.

In larger towns and cities, the existence of public transportation allows for a larger hinterland in which peri-urban farmers and sub-urban commuters coexist. As towns centers grow, demand increases for peri-urban products and for sub-urban lifestyle (and lower rents). If unconstrained, this process would predict a proportional expansion of their hinterland. If this is true, we would expect the cell distribution of buildup density to remain constant over time, leading to a noticeable urban sprawl. The size of this low density 'crown' around towns and cities is nonetheless likely to be constrained by commuting time. If so, we expect town expansion to partially or fully crowd out its hinterland. The data strongly rejects the first prediction: as the national proportion of fully built-up cells increase, the proportion of partially built-up cells remains essentially unchanged. The idea that partial buildup follows something similar to a power law (or Zipf Law) is also strongly rejected. These findings suggest that, over the study period, cities grew at least partially at the expense of their hinterland.

We also document the role played by roads and past settlement in attracting new settlement. We find strong evidence that agglomeration forces are at work, both within periods and across time.

Roads also prove to be a strong magnet for new settlements, with strong and lasting predictive effects associated with roads present at the beginning of our study period. We calibrate transition matrices to predict the distribution of build density at the middle and end of this century. Predictions do not vary much depending on which data years we use to calibrate the matrix. We find that, if current trends continue, Ghana will experience considerable urban growth, and that this growth will take place mostly in concentrated fashion, not as urban sprawl. We also expect a continuation of the current trend towards the rise of small and middle-size towns.

These findings have important implications for targeting provision of services in the coming years. The strong inertia in human settlement means that expansion in the provision of services can focus on the existing concentration of population, taking into account the fact that towns of all sizes, not just the large cities of Accra and Kumasi, will experience growth. Outside of towns and cities, the relatively low density of cultivation means that rural settlements are not regularly spaced, making it more difficult to plan the provision of rural amenities. But the fact that rural settlements increases fastest along roads means that expansion of provision will, at least initially, focus on roads. Since this is likely to further reinforce the tendency for people to locate near roads, opening new roads to currently unserved rural communities will be necessary to prevent a lopsided rural development pattern.

References

Ambrus, Attila, Erica Field, and Robert Gonzalez (2020). “Loss in the Time of Cholera: Long-run Impact of a Disease Epidemic on the Urban Landscape”, *American Economic Review*, 110(2): 1-52.

Arshad, Sidra, Shougeng Hu, and Badar Nadeem Ashraf (2018). “Zipf’s law and city size distribution: A survey of the literature and future research agenda Author links open overlay panel”, *Physica A: Statistical Mechanics and its Applications*, 492: 75-92

Balboni, C., Bryan, G., Morten, M., & Siddiqi, B. (2020). “Transportation, Gentrification, and Urban Mobility: The Inequality Effects of Place-Based Policies”, Stanford University (mimeo)

Banerjee, Abhijit , Esther Duflo and Nancy Qian (2020). "On the Road: Access to Transportation Infrastructure and Economic Growth in China", *Journal of Development Economics*, 145: 1-36

Barbour, E, CC Davila, S Gupta, C Reinhart, J Kaur, and MC González (2019). “Planning for sustainable cities by estimating building occupancy with mobile phones”. *Nature Communications*, 10(1): 1-10

Baum-Snow, Nathaniel, Loren Brandt, J. Vernon Henderson, Matthew A. Turner and Qinghua Zhang (2017). "Roads, Railroads and Decentralization of Chinese Cities", *Review of Economics and Statistics,* 99(3): 435-48.

Bellefon, Marie-Pierre de, Pierre-Philippe Combes, Gilles Duranton, and Laurent Gobillon (2019). “Delineating urban areas using building density”, *Journal of Urban Economics* (in press)

Bird, Julia and Stéphane Straub (2020). "The Brasília Experiment: Road Access and the Spatial Pattern of Long-term Local Development in Brazil", *World Development*, 127: (in press)

Bleakley, Hoyt and Jeffrey Lin (2012). "Portage and Path Dependence", *Quarterly Journal of Economics*, 127(2):587-644.

Boserup, Esther (1965). *The Conditions of Agricultural Growth: The Economics of Agrarian Change under Population Pressure*, George Allen and Unwin Ltd, London

Bryan, Gharad, Edward Glaeser, and Nick Tsivanidis (2019). “Cities in the Developing World”, NBER Working Paper 26390: http://www.nber.org/papers/w26390

Chandra, Amitabh and Eric Thompson (2000). "Does public infrastructure affect economic activity? Evidence from the rural interstate highway system", *Regional Science and Urban Economics*, 30(4): 457-490.

Christaller, Walter (1933). *Die zentralen Orte in Süddeutschland (The Central Places in Southern Germany)*, Gustav Fischer, Jena.

Collin, Matt (2020). “Tribe or title? Ethnic enclaves and the demand for formal land tenure in a Tanzanian slum”, *Economic Development and Cultural Change* (forthcoming)

Dell, Melissa, and Benjamin Olken (2020). “The Development Effects of the Extractive Colonial Economy: The Dutch Cultivation System in Java.” *Review of Economic Studies,* 87(1): 164-203.

Dennis M, Scaletta KL, James P (2019). “Evaluating urban environmental and ecological landscape characteristics as a function of land-sharing-sparing, urbanity and scale”. *PLoS ONE*, 14(7): e0215796. https://doi.org/10.1371/journal.pone.0215796

Desmet, Klaus and Marcel Fafchamps (2006). "Employment Concentration across US Counties", *Regional Science and Urban Economics*, 36(4): 482-509.

Desmet, Klaus, and J. Vernon Henderson (2015). “The Geography of Development within Countries.” In *Handbook of Regional and Urban Economics, vol. 5*, edited by Duranton, Henderson, and Strange, pp. 1457–1517. Amsterdam: Elsevier.

Donaldson, David (2018). "Railroads of the Raj: Estimating the Impact of Transportation Infrastructure", *American Economic Review*, 108(4-5): 899-34. April

Duranton, G (2015), “Growing through Cities in Developing Countries”, *World Bank Research Observer*, 30(1): 39-73. doi:doi:10.1093/wbro/lku006

European Commission- Joint Research Centre, “Global Human Settlement Layer,” https://ghsl.jrc.ec.europa.eu

Faber, Benjamin (2014). "Trade Integration, Market Size, and Industrialization: Evidence from China’s National Trunk Highway System", *Review of Economic Studies*, 81(3):1046-1070.

Faber, Benjamin and Cecile Gaubert (2019). “Tourism and Economic Development: Evidence from Mexico’s Coastline”, *American Economic Review*, 109(6),: 2245-93

Fafchamps, Marcel and Forhad Shilpi (2003). "The Spatial Division of Labor in Nepal", *Journal of Development Studies*, 39(6): 23-66.

Fafchamps, Marcel and Forhad Shilpi (2005). "Cities and Specialization: Evidence from South Asia", *Economic Journal*, 115(503): 477-504.

Fafchamps, Marcel, Michael Koelle and Forhad Shilpi (2017). "Gold Mining and Proto-Urbanization: Recent Evidence from Ghana", *Journal of Economic Geography*, 17(5): 975-1008, September.

Fujita, Masahisa, Paul Krugman, and Anthony J. Venables (1999). *The Spatial Economy: Cities, Regions and International Trade*, MIT Press, Cambridge Massachusetts.

Gabaix, Xavier (1999). “Zipf’s Law for Cities: An Explanation”, *Quarterly Journal of Economics*, 114(3): 739–67, August. https://doi.org/10.1162/003355399556133

Gechter, Michael and Nick Tsivanidis (2020). “Spatial Spillovers from Urban Renewal: Evidence from the Mumbai Mills Redevelopment”, Penn State University (mimeo)

Glaeser, E.L. and J.D. Gottlieb (2009), “The Wealth of Cities: Agglomeration Economies and Spatial Equilibrium in the United States”, *Journal of Economic Literature*, 47(4), 983-1028.

Glaeser, Edward L., Sari Pekkala Kerr, and William R. Kerr (2015). "Entrepreneurship and Urban Growth: An Empirical Assessment with Historical Mines", *Review of Economics and Statistics*, 97(2): 498-520.

Gollin, Douglas, Rémi Jedwab, and Dietz Vollrath (2016). "Urbanization with and without

Industrialization", *Journal of Economic Growth*, 21(1):35-70

Goltz, Jan von der, and Prabhat Barnwal (2019). "Mines: The Local Welfare Effects of Mineral Mining in Developing Countries", Journal of Development Economics, 139: 1-16.

Gonzalez-Navarro, Marco, and Matthew Turner (2018). "Subways and Urban Growth: Evidence from Earth", *Journal of Urban Economics*, 108: 85-106.

Gonzalez-Navarro, Marco and Climent Quintana-Domeque (2016). “Paving Streets for the Poor: Experimental Analysis of Infrastructure Effects” (2016), *Review of Economics and Statistics*, May, 98(2); 254-67.

Gonzalez, MC, CA Hidalgo, and AL Barabasi (2008). “Understanding individual human mobility patterns”. *Nature*, 453(7196): 779-82

Harari, Mariaflavia (2019). “Cities in Bad Shape: Urban Geometry in India”, *American Economic Review* (forthcoming)

He, Yiming (2020). “The Long-term Impact of Slum Demolition on its Residents: Evidence from Victorian London”, Stanford University (mimeo)

Henderson, J. Vernon (1985). *Economic Theory and the Cities*, 2nd edition, Academic Press.

Henderson, J. Vernon, Adam Storeygard, and David Weil (2012). "Measuring Economic Growth from Outer Space", *American Economic Review*, 102(2): 994-1028.

Henderson, Vernon, Tim Squires, Adam Storeygard and David N. Weil (2018). “The Global Spatial Distribution of Economic Activity: Nature, History, and the Role of Trade”, *Quarterly Journal of Economics,* 133(1): 357-406.

Henderson, J. Vernon and Matthew A. Turner (2020). “Urbanization in the Developing World: Too Early or Too Slow?”, *Journal of Economic Perspectives*, 34(3): 150–73

Hornbeck, Richard, and Daniel Keniston. 2017. "Creative Destruction: Barriers to Urban Growth and the Great Boston Fire of 1872." *American Economic Review*, 107 (6): 1365-98.

Isard, Walter (1956). *Location and Space-Economy: A General Theory Relating to Industrial Location, Market Areas, Land Use, and Urban Structure*, John Wiley and Sons, New York.

Jacobs, Jane (1969). *The Economy of Cities*, Random House, New York.

Jedwab, Rémi (2014). "Urbanization without Structural Transformation: Evidence from Consumption Cities in Africa", George Washington University, mimeograph.

Jedwab, R, E Kerby, and A Moradi (2017), “History, Path Dependence and Development:

Evidence from Colonial Railroads, Settlers and Cities in Kenya", *Economic Journal*, 127(603): 1467-94, August

Jedwab, R., and A. Moradi (2016), "The Permanent Effects of Transportation Revolutions in Poor Countries: Evidence from Africa", *Review of Economics and Statistics*, 98(2), 268-284.

Lall, Somik, Vernon Henderson and Anthony Venables. (2017). *Africa's Cities: Opening Doors to the World*. Washington, DC: World Bank.

Michaels, Guy (2008). "The Effect of Trade on the Demand for Skill: Evidence from the Interstate Highway System", The *Review of Economics and Statistics*, 90(4): 683-701

Michalopoulus, Stelios and Elias Papaioannou (2013). "Pre-Colonial Ethnic Institutions and Contemporary African Development", *Econometrica*, 81(1): 113-152.

Pesaresi, Martino, Daniele Ehrlich, Stefano Ferri, Aneta J. Florczyk, Sergio Freire, Matina Halkia, Andreea Julea, Thomas Kemper, Pierre Soille, and Vasileios Syrris (2016). *Operating procedure for the production of the Global Human Settlement Layer from Landsat data of the epochs 1975, 1990, 2000, and 2014*, JRC Technical Report EUR 27741 EN; doi:10.2788/253582 (online)

Redding, Stephen J. (2020). "Trade and Geography", NBER Working Paper No. 27821, September 2020

Salop, Steven C. (1979). "Monopolistic Competition with Outside Goods", *Bell Journal of Economics*, RAND Corporation, 10(1): 141-56, Spring.

Seto KC, Fragkias M, Güneralp B, Reilly MK (2011). "A Meta-Analysis of Global Urban Land Expansion". *PLoS ONE*, 6(8): e23777.

Tsivanidis, Nick (2020). "Evaluating the Impact of Urban Transit Infrastructure: Evidence from Bogotá's TransMilenio", UC Berkeley (mimeo)

UN Press Release (2013), "World population projected to reach 9.6 billion by 2050 with most growth in developing regions, especially Africa says UN", 13 June 2013

UNDP (2017). "Income Inequality Trends in Sub-Saharan Africa: Divergence, Determinants, and Consequences", United Nations Development Program, Regional Bureau for Africa, New York.

Vollset, Stein Emil, Emily Goren, Chun-Wei Yuan, Jackie Cao, Amanda E Smith, Thomas Hsiao, et al. (2020). "Fertility, mortality, migration, and population scenarios for 195 countries and territories from 2017 to 2100: a forecasting analysis for the Global Burden of Disease Study". *The Lancet*, DOI:https://doi.org/10.1016/S0140-6736(20)30677-2

von Thunen, Johann Heinrich (1966), *Isolated State: an English Edition of Der Isolierte Staat*, Oxford: Pergamon Press [original edition 1826]

Zhang J, He X, Yuan X-D (2020). "Research on the relationship between Urban economic development level and urban spatial structure—A case study of two Chinese cities". *PLoS ONE*, 15(7): e0235858. https://doi.org/10.1371/journal.pone.0235858

Table 1. Built-up area

Year	Proportion of built-up area	Proportion of built-up area in N1 cells	Proportion of built-up area in N2 cells	Share of cells that are empty	Share of non-empty cells that are fully built-up
1975	0.43%	0.43%	0.42%	98.50%	1.96%
1990	0.80%	0.80%	0.80%	97.48%	6.61%
2000	1.19%	1.19%	1.19%	96.52%	8.41%
2014	1.85%	1.85%	1.84%	93.78%	6.70%

Notes: Total number of observations is 3,853,273. N1 cells are the 8 rectangles surrounding the cell. N2 cells are the 16 rectangles surrounding N1 cells. The number of N1 cells is 8 for all but 27,241 cells. The number of N2 cells is 16 for all but 54,324 cells.

Table 2. Presence of roads in a cell

Road quality	1976	1986	2008	2014
Class 1 road	0.61%	0.67%		
Class 2 road	0.83%	0.98%		
Class 3 road	1.03%	1.15%		
Track	0.55%	0.60%		
Unknown	0.01%	0.00%		
Any road	2.99%	3.35%		
Intersection	0.03%	0.04%		
Administrative road definition				
Highways			n.a.	0.00%
Primary			0.63%	0.25%
Secondary			0.36%	1.05%
Tertiary			0.82%	1.66%
Local road			n.a.	0.28%
Any road			1.80%	3.12%
Intersection			n.a.	0.23%

Note: Number of observations: 3,880,380. Unknown roads in 1976 and 1986 denote a very small proportion of roads of unknown quality. Road quality in 1976 and 1986 does not align with administrative road definitions in 2008 and 2014, which themselves differ as well in spite of the partial similarity in names. Highways in 2014 include a single motorway/freeway from Accra to the nearby city of Tema and is present in 113 cells only. Averages for road availability in the 8 (N1) and 16 (N2) neighboring cells are not shown here to save space. They are essentially identical to the averages reported here since they are averages over essentially the same cells (except for small differences at the edges). Intersections cover all classes of road available in the data.

Table 3. Time-invariant features of cells

	Average	Min	Max
Latitude	7.96	4.74	11.17
Longitude	-1.21	-3.26	1.20
Presence of water body	2.7%		
Presence of land border	0.1%		
Presence of sea	0.1%		
Number of cells	3,853,273		
Soil quality			
Poor	38.1%		
Moderate	47.8%		
Good	7.4%		
Hydromorphic	1.7%		
Waterlogged/Steep terrain	5.0%		
Climate			
Moist or dry	17.1%		
Sub-humid	70.2%		
Humid	12.8%		
Number of cells with soil and climat	3,811,014		

Notes: Latitude and longitude reported in decimal degrees. Water bodies include rivers, lakes and lagoons. Hydromorphic soils are those affected by water (e.g., river banks, tidal estuaries). Waterlogged soils and steep terrain are those considered unsuitable for agriculture or house construction. Of all the cells covering the area of Ghana, 27,107 are excluded because they are fully covered by water; most of this is Lake Volta.

Table 4. Breakdown of built-up proportion by agro-ecological zones

Year	Soil quality					Soil humidity			No info
	Poor	Moderate	Good	.	ep	Moist/dry	humid	Humid	
1975	0.55%	0.30%	0.21%	0.41%	0.09%	0.21%	0.47%	0.15%	3.56%
1990	0.89%	0.70%	0.43%	0.81%	0.23%	0.37%	0.88%	0.42%	5.88%
2000	1.31%	1.08%	0.66%	1.07%	0.29%	0.50%	1.32%	0.66%	8.09%
2014	2.03%	1.69%	1.02%	2.71%	0.75%	0.69%	2.07%	1.34%	9.32%
N. obs.	1,447,084	1,816,564	282,295	64,568	189,485	648,635	2,666,937	484,424	53,277

Table 5. Breakdown of built-up proportion by presence of a road

Year	Road in 1976		Road in 1986		Road in 2008		Road in 2014	
	No	Yes	No	Yes	No	Yes	No	Yes
1975	0.35%	2.94%	0.34%	3.01%	0.37%	3.53%	0.22%	6.84%
1990	0.67%	5.21%	0.64%	5.39%	0.70%	6.23%	0.46%	11.44%
2000	1.01%	7.15%	0.97%	7.44%	1.05%	8.61%	0.74%	14.96%
2014	1.59%	10.00%	1.55%	10.35%	1.66%	12.21%	1.29%	19.17%
N. obs.	3,737,881	115,392	3,723,869	129,404	3,783,821	69,452	3,732,420	120,853

Table 6. Breakdown of built-up proportion by boundary status

	Presence on the cell of:			
Year	Water body	Land border	Sea	None
1975	0.01%	0.27%	30.75%	0.42%
1990	0.03%	0.52%	49.73%	0.80%
2000	0.06%	0.70%	60.40%	1.19%
2014	0.20%	1.11%	61.87%	1.86%
N. obs.	105,035	4,041	1,978	3,742,297

Table 7. Correlation in buildup between neighboring cells

	1975		1990		2000		2014	
	Coef.	Std.err.	Coef.	Std.err.	Coef.	Std.err.	Coef.	Std.err.
y(n1)	1.3102	0.0008	1.3761	0.0007	1.3953	0.0007	1.3708	0.0007
y(n2)	-0.3237	0.0008	-0.3888	0.0008	-0.4060	0.0007	-0.3807	0.0008
Intercept	0.0000	0.0000	0.0001	0.0000	0.0001	0.0000	0.0002	0.0000
R-square	0.7952		0.8402		0.8666		0.8507	
N. obs.	3,853,228		3,853,228		3,853,228		3,853,228	

Note: the dependent variable is y. There are no controls included.

Table 8. Correlation in buildup between neighboring cells over time

	1990		2000		2014	
Regressors in 1975						
y	0.8691	0.0008	0.7273	0.0013	0.6123	0.0018
y(n1)	0.3420	0.0016	0.4390	0.0025	0.5221	0.0036
y(n2)	0.1605	0.0013	0.4304	0.0021	0.5568	0.0030
intercept	0.0022	0.0000	0.0051	0.0000	0.0113	0.0000
	0.7453		0.5945		0.4352	
			2000		2014	
Regressors in 1990						
y			0.8961	0.0007	0.7575	0.0012
y(n1)			0.1839	0.0013	0.3688	0.0024
y(n2)			0.1025	0.0011	0.1477	0.0019
intercept			0.0024	0.0000	0.0082	0.0000
			0.812		0.6029	
					2014	
Regressors in 2000						
y					0.8395	0.0008
y(n1)					0.2505	0.0016
y(n2)					0.0252	0.0012
intercept					0.0052	0.0000
					0.7758	

Notes: each panel is a separate regression. The total number of observations is 3,853,228 in each regression.

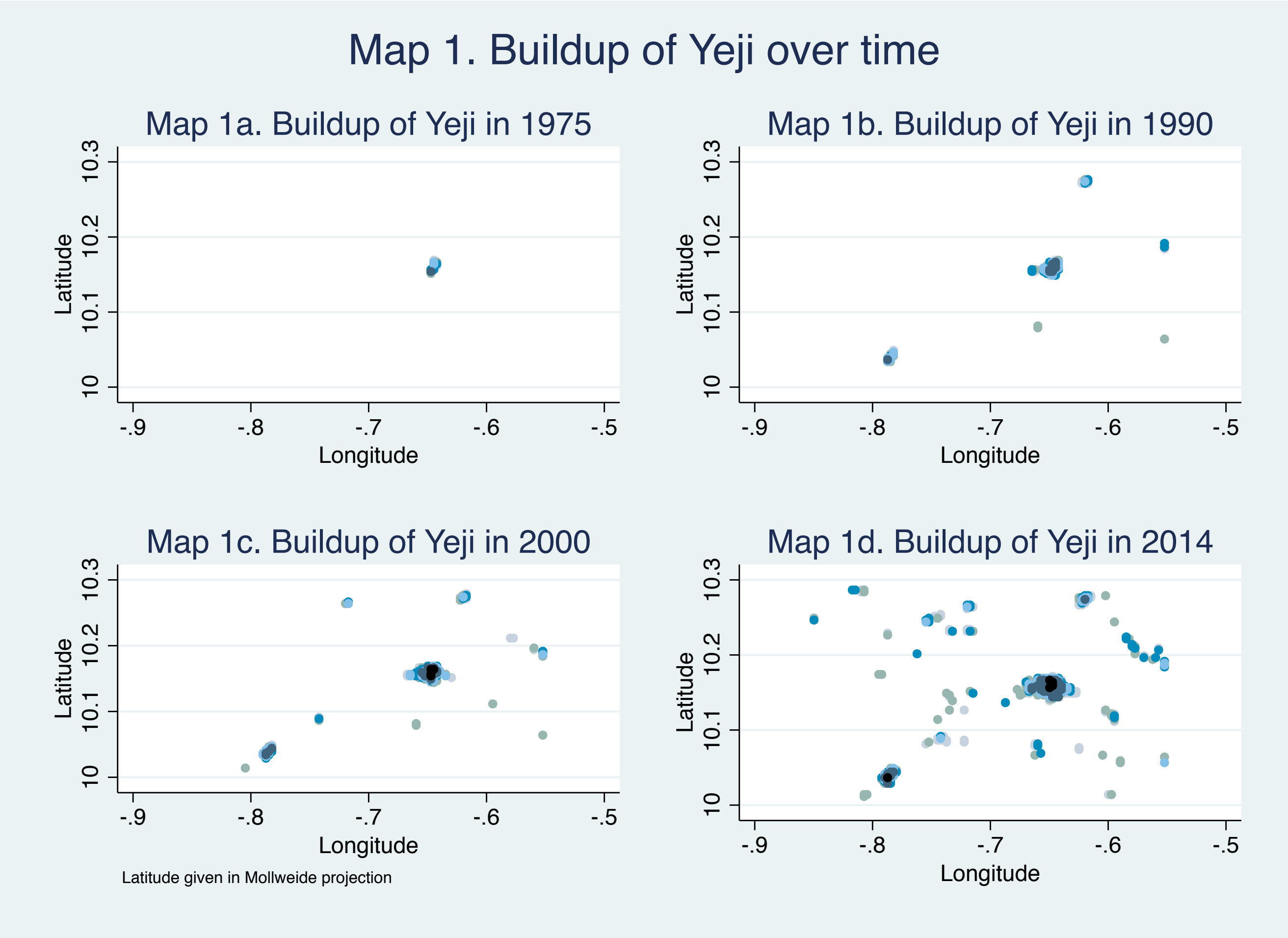
Map 1. Buildup of Yeji over time
Map 1a. Buildup of Yeji in 1975
Map 1b. Buildup of Yeji in 1990
Map 1c. Buildup of Yeji in 2000
Map 1d. Buildup of Yeji in 2014
Latitude
10
10.1
10.2
10.3
Longitude
-.9
-.8
-.7
-.6
-.5
Latitude given in Mollweide projection

Map 1e: Satellite view of the Yeji area in 2020

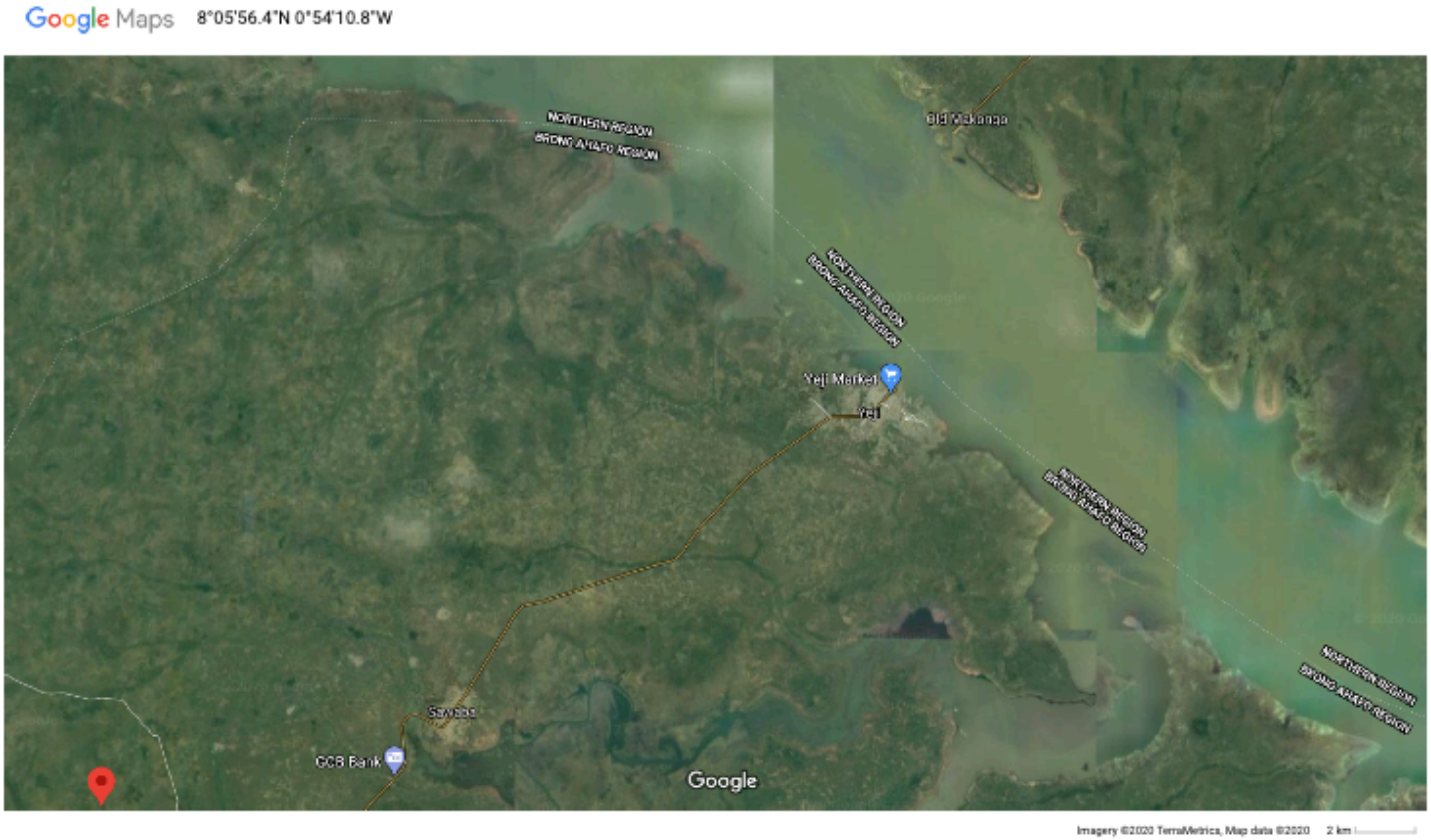

Map 1f: Road map of the Yeji area on 2020

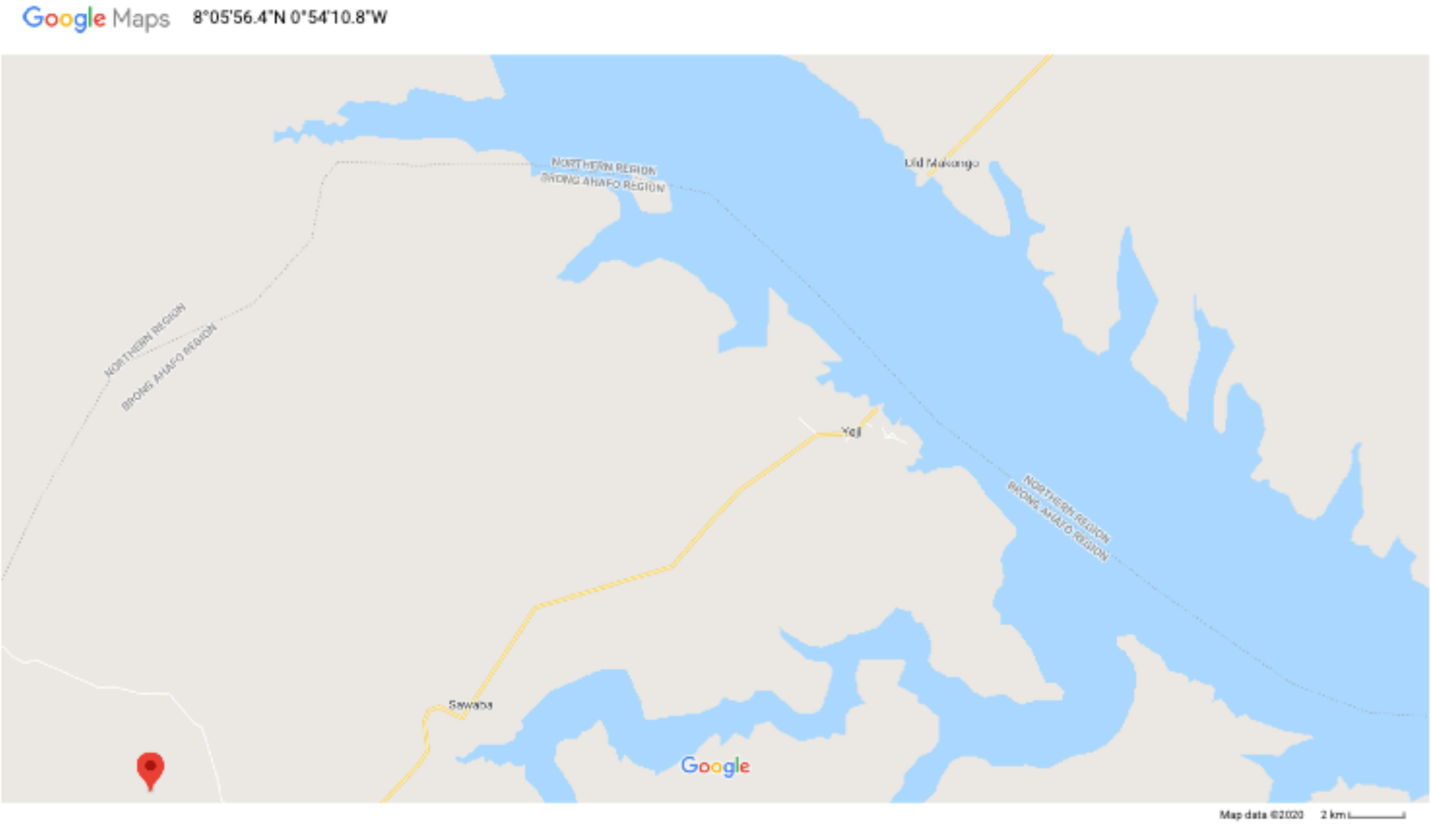

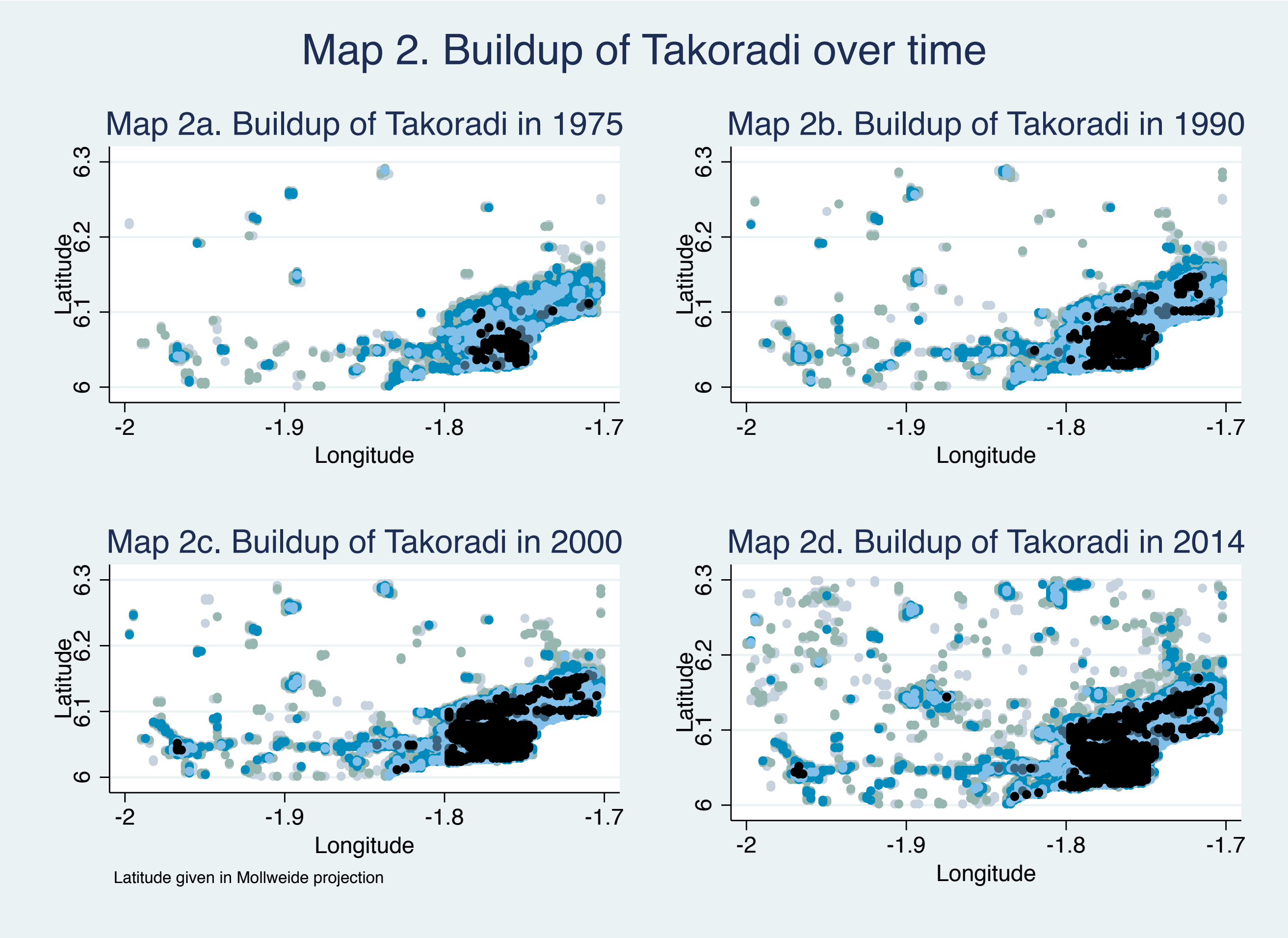

Map 2. Buildup of Takoradi over time
Map 2a. Buildup of Takoradi in 1975
Map 2b. Buildup of Takoradi in 1990
Map 2c. Buildup of Takoradi in 2000
Map 2d. Buildup of Takoradi in 2014
Latitude
6
6.1
6.2
6.3
Longitude
-2
-1.9
-1.8
-1.7
Latitude given in Mollweide projection

Map 2e: Satellite view of the Takoradi area in 2020

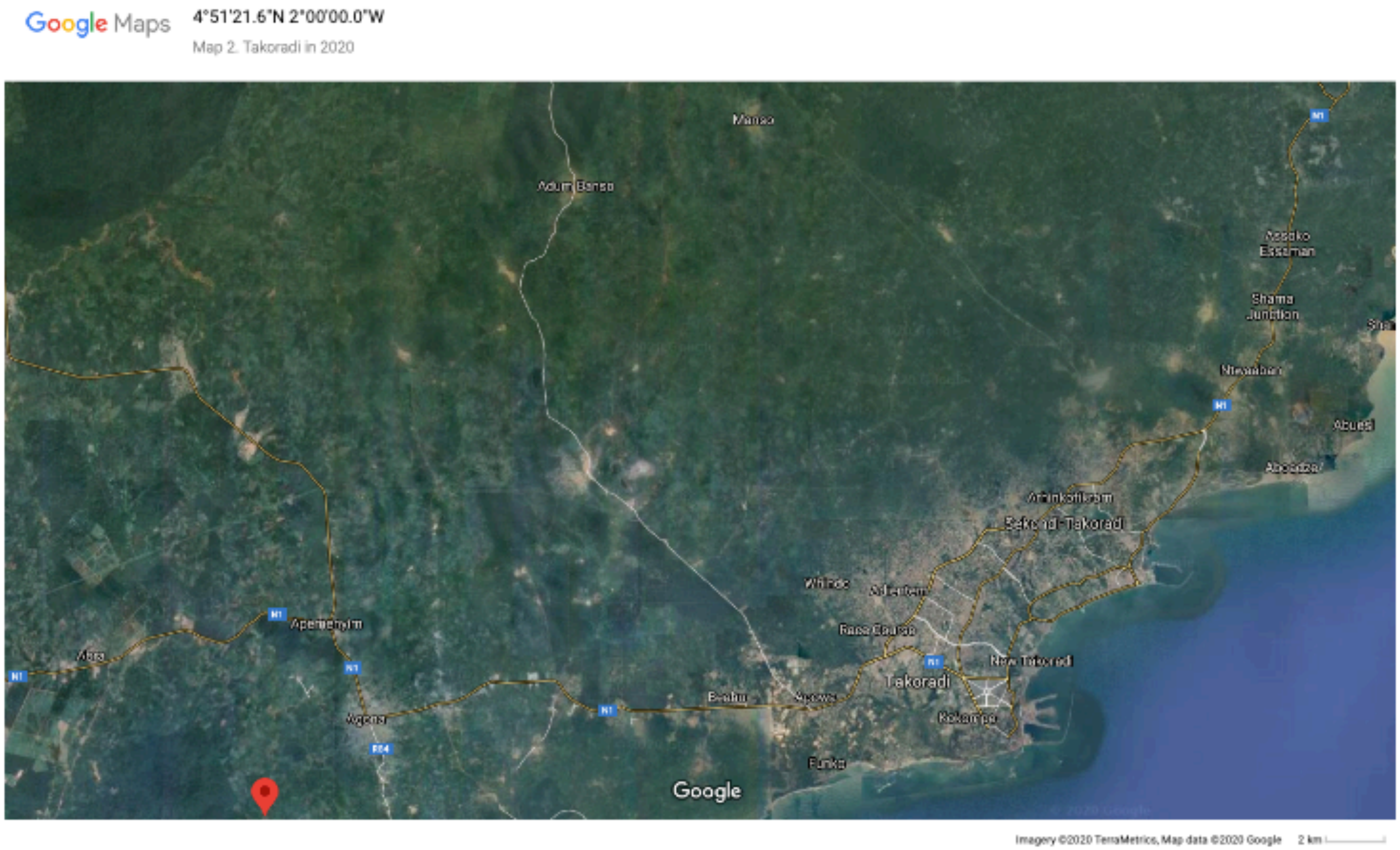

Map 2f: Road map of the Takoradi area in 2020

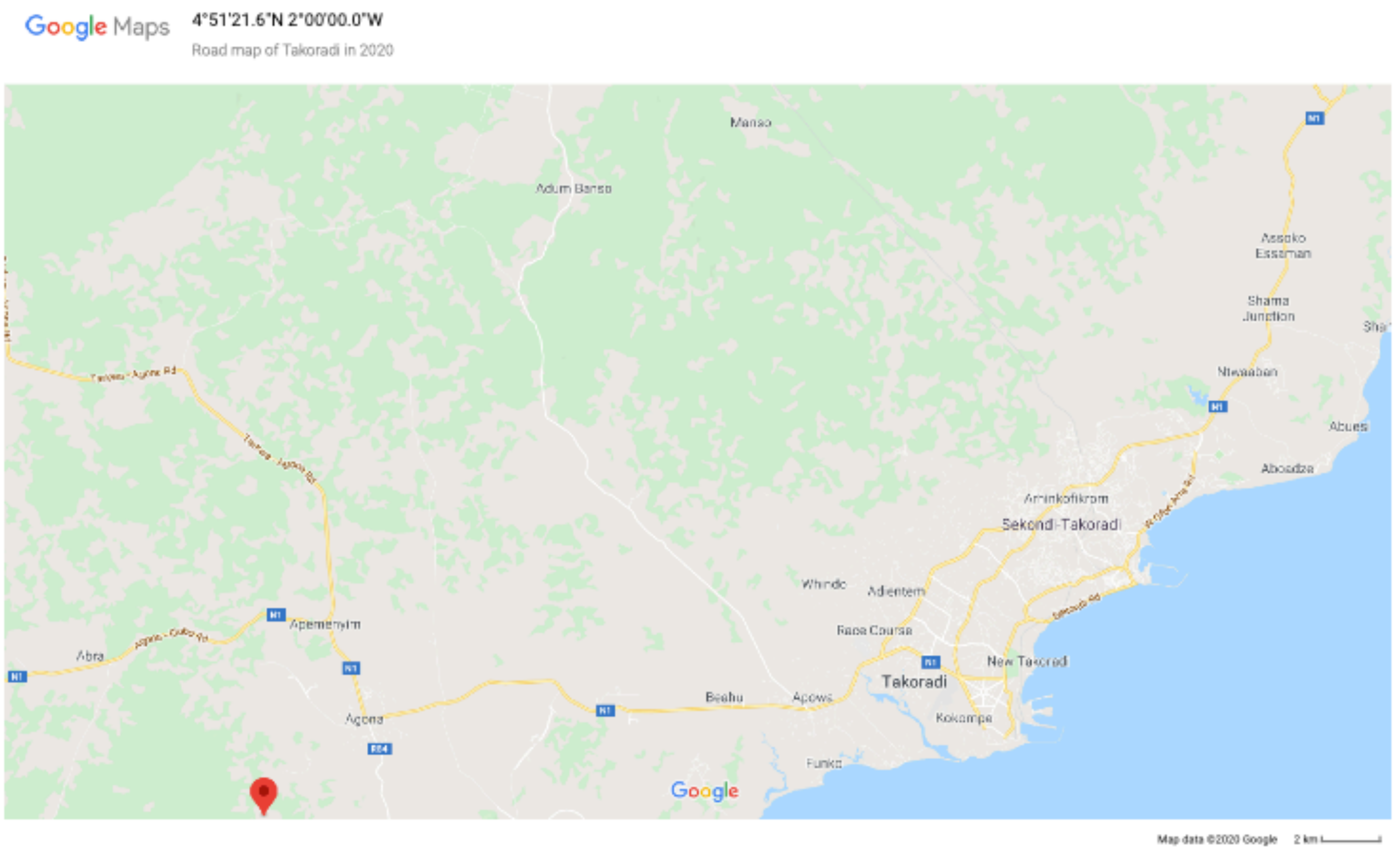

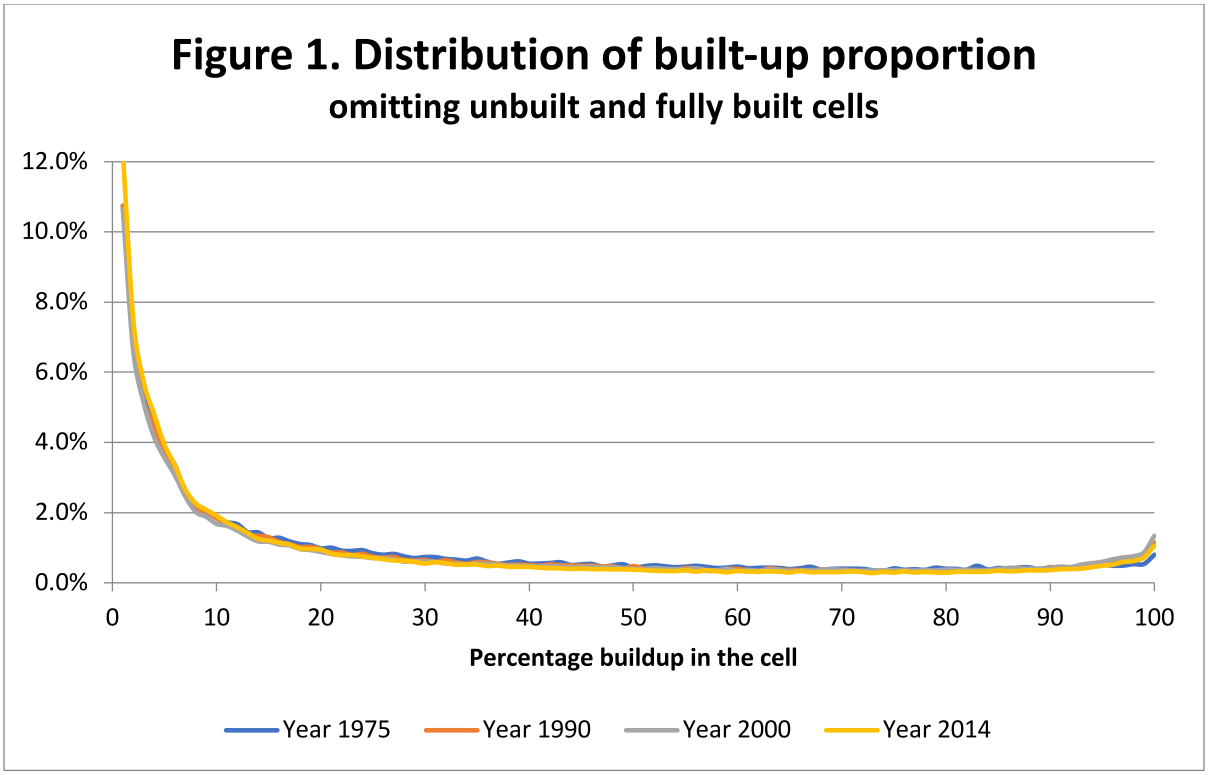

Notes: After dropping unbuilt cells (which constitute more than 90% of cells in all years) and the fully built-up cells (which are discussed separately in the text), Figure 1 plots four histograms of the frequency distribution of partially built-up cells by their built-up proportion. The horizontal axis shows the built-up proportion of the cell. The vertical axis shows the proportion of cells in each buildup percentage interval. There is one histogram for each year. To facilitate viewing, each of the four histograms is represented as a colored line and the four histograms are overlaid on top of each other. This is done to facilitate a visual comparison of the frequency distribution across years.

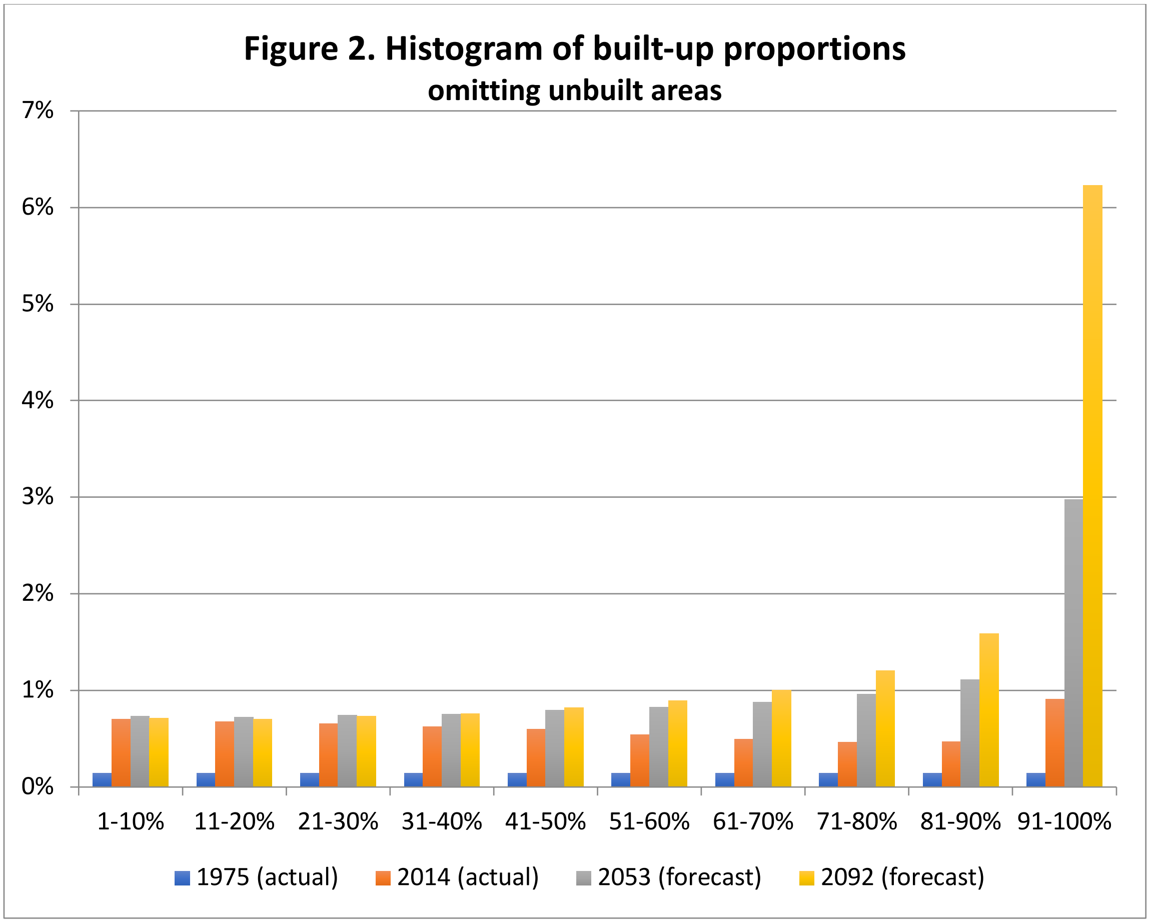

Notes: Figure 2 shows a stacked histogram of the frequency distribution of built-up proportion divided into deciles of buildup proportion in 1975. Fully built cells are included in the top decile. Since an overwhelming proportion of cells are unbuilt in all years, they are omitted from the Figure for the sake of clarity. The 1975 and 2014 values are taken directly from the data. The 2053 and 2092 forecasts are obtained as follows. We first form the decile transition matrix using data from 1975 and 2014 (a 39 year interval). We then project the built-up frequency distribution from 2014 to 2053 and 2092 as two iterations of the transition matrix. The resulting forecasts should be understood as predictions of what the distribution of buildup will look like if the transition matrix remains unchanged. Similar forecasts are obtained if we use transition matrices constructed using different data years. The forecast do not specify which cells will be built.

Figure 3a. Histogram of built-up: cells with roads in 1976
omitting unbuilt areas

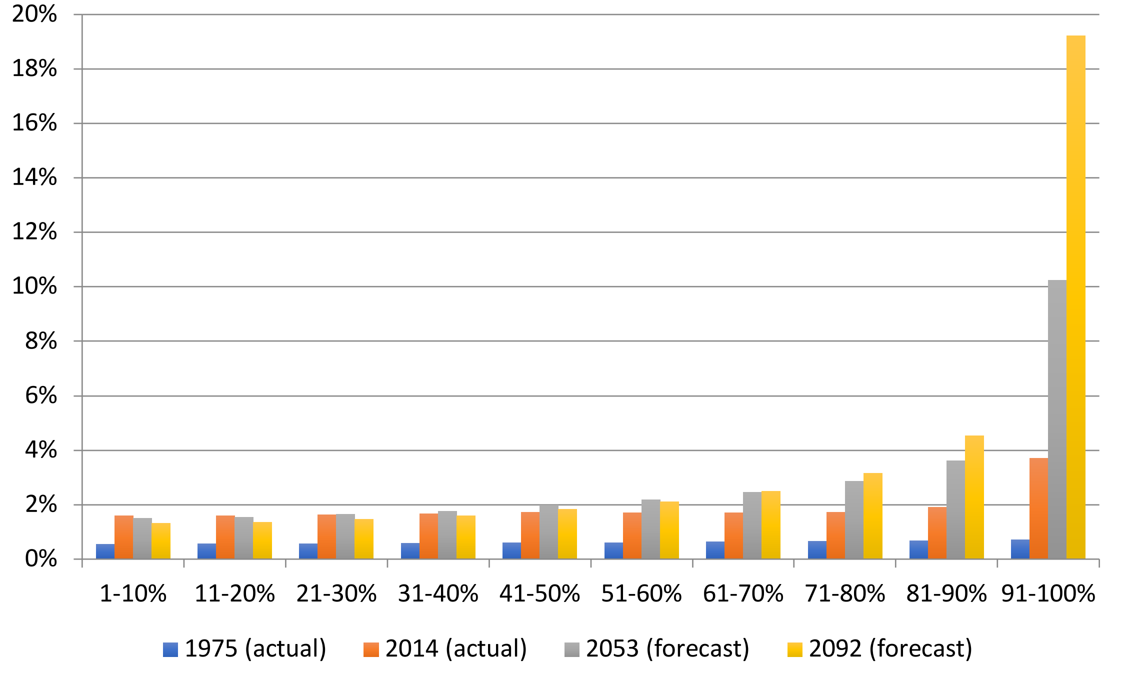

Figure 3b. Histogram of built-up: cells w/out road in 1976
omitting unbuilt areas

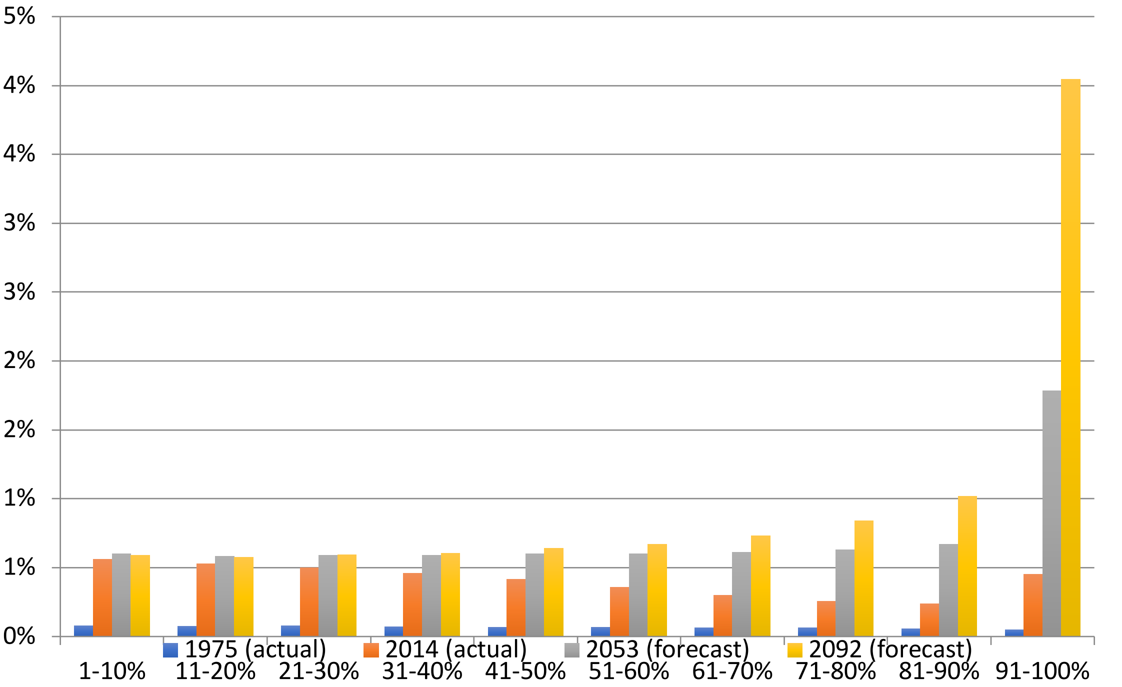

Notes for Figures 3a and 3b: The Figures show a stacked histogram of the frequency distribution of built-up proportions divided into 1975 deciles. In Figure 3a only those cells with a road in 1976 are used in the histograms. Figure 3b does the same thing for cells without a road in 1976. In both Figures, fully built cells are included in the top decile. Since an overwhelming proportion of cells are unbuilt in all years, they are omitted from the Figures for the sake of clarity. The 1975 and 2014 values are taken directly from the data. The 2053 and 2092 forecasts are obtained as follows. We first form the decile transition matrix using data from 1975 and 2014, only using cells with a road in 1976 (for Figure 3a) or those without a road in 1976 (for Figure 3b). We then separately project the built-up frequency distribution from 2014 to 2053 and 2092 as two iterations of the transition matrix. The resulting forecasts should be understood as predictions of what the distribution of buildup will look like in 1976 if the transition matrices remain unchanged. Similar forecasts are obtained if we use transition matrices constructed using different data years. The forecast do not specify which cells will be built.

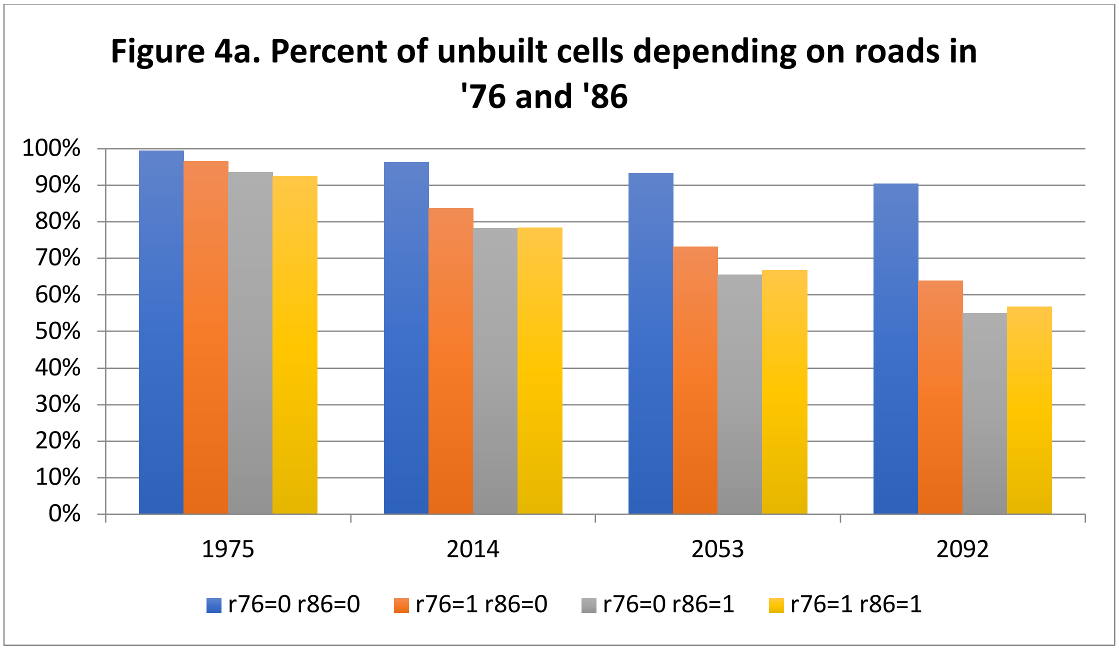

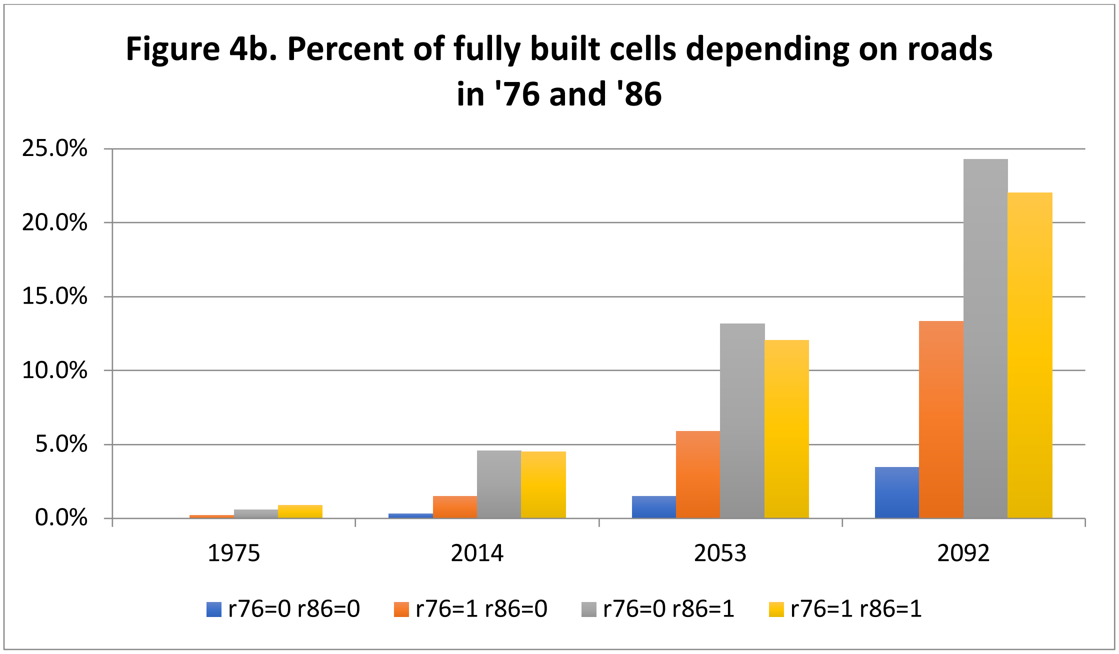

Notes: Figure 4a shows the percent of unbuilt cells in various years, conditional on whether the cell had a road in 1976 (r76=1) and a road in 1986 (r86=1). Some cells with a road in 1976 no longer have one in 1986 (e.g., because the road was not maintained or was moved). Figure 4b shows the percent of fully built cells in various years, also depending on whether the cell had a road in 1976 (r76=1) and a road in 1986 (r86=1). Values for 1975 and 2014 are take from the data. Values for 2053 and 2092 are forecasts obtained in the same manner as for Figures 3a and 3b, except that we now use four different transition matrices and four 2014 buildup vectors from which to iterate.

Figure 5. Plot of lagged buildup coefficients

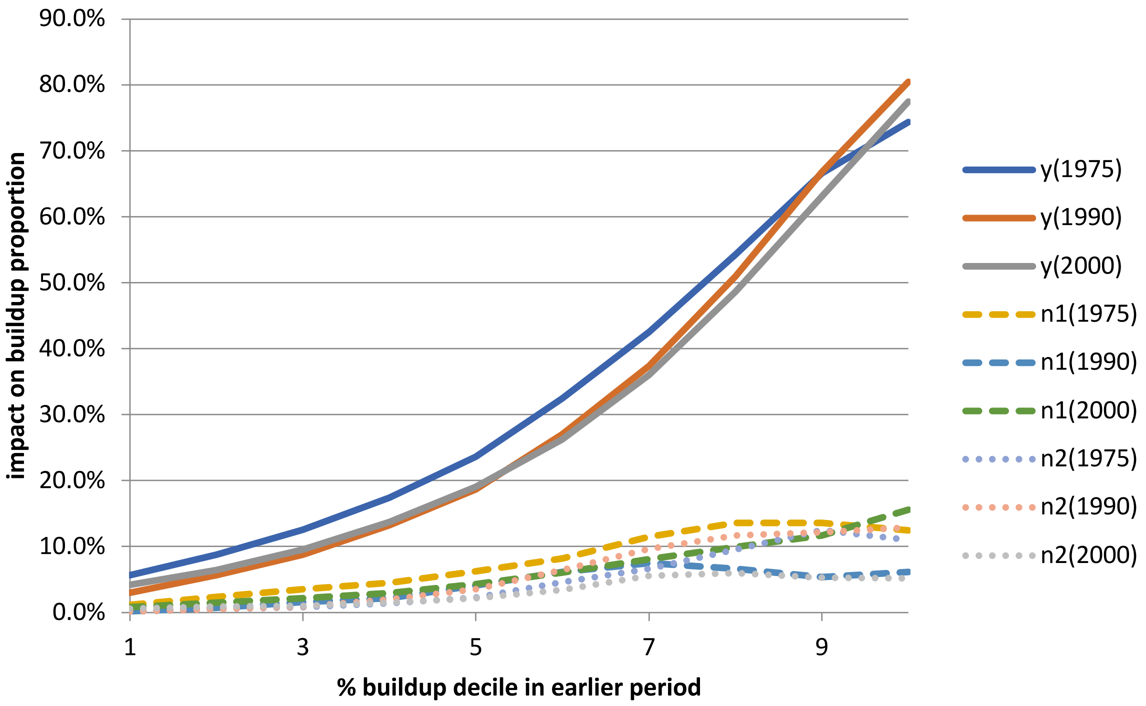

Notes: Figure 5 summarizes the coefficients from three separate regressions. The dependent variable in the three regressions is the proportion of a cell that is built up in 1990, 2000, and 2014 respectively. Each regression includes lagged values of buildup in the cell and in the neighboring cells n1 and n2. The lagged values are for 1975, 1990, and 2000 respectively. Each of the three lagged buildup variables appears in the regression as a series of 10 dummies, one per buildup decile. The Figure shows the estimated decile coefficients in each of the three regressions for (1) buildup in the cell itself [lines y(1975), y(1990) and y(2000)]; buildup in neighboring cells n1 [lines n1(1975), n1(1990), and n1(2000)]; and buildup in neighboring cells n2 [lines n2(1975), n2(1990), n2(2000)]. Each regression also includes the following time invariant regressors: longitude, latitude, and their product [three variables]; soil humidity and soil quality dummies [six variables]; and dummies for the presence of a water body, a land border, or the sea in the cell, the neighboring cells n1 and the neighboring cells n2, respectively [9 variables]. The number of observations in each regression is just shy of 3.8 million. R-squares are 0.799, 0.845 and 0.796, respectively. All coefficients shown here are significant at the 1% or better.

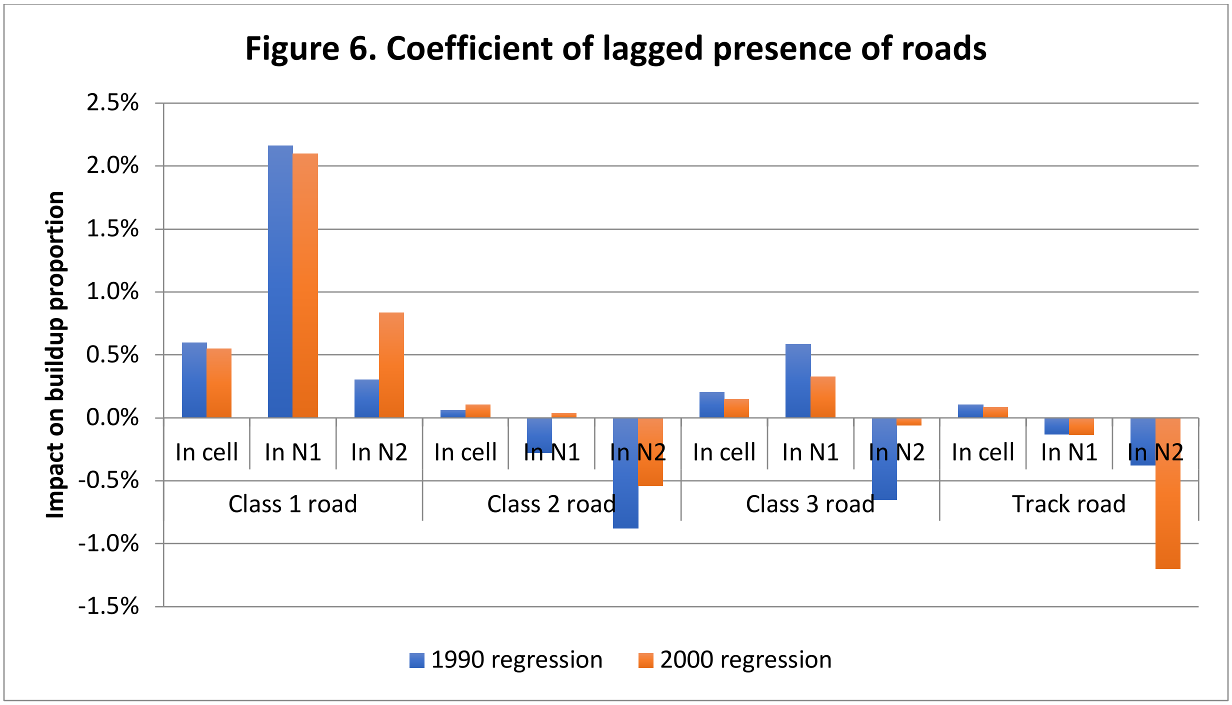

Notes: Figure 6 summarizes the coefficients from two separate regressions. The dependent variable in the two regressions is the proportion of a cell that is built up in 1990 and 2000, respectively. The Figure shows the coefficients of the presence of a road of a certain class (i.e., category) in the cell, the neighborhood N1 and the neighborhood N2. The road quality ordering is: class 1 (top category), class 2, class 3, and track (bottom category). In the 1990 regression, the road data refers to 1976; in the 2000 regression, the road data refers to 1986. Each of the regressions also includes all the regressors that were present in those for Figure 5. More precisely, each regression includes lagged values of buildup in the cell and in the neighboring cells n1 and n2. The lagged values are for 1975 and 1990, respectively. Each of the three lagged buildup variables appears in the regression as a series of 10 dummies, one per buildup decile. Each regression also includes the following time invariant regressors: longitude, latitude, and their product [three variables]; soil humidity and soil quality dummies [six variables]; and dummies for the presence of a water body, a land border, or the sea in the cell, the neighboring cells n1 and the neighboring cells n2, respectively [9 variables]. The number of observations in each regression is just shy of 3.8 million. R-squares are 0.796 and 0.796, respectively. All coefficients shown here are significant at the 1% or better.

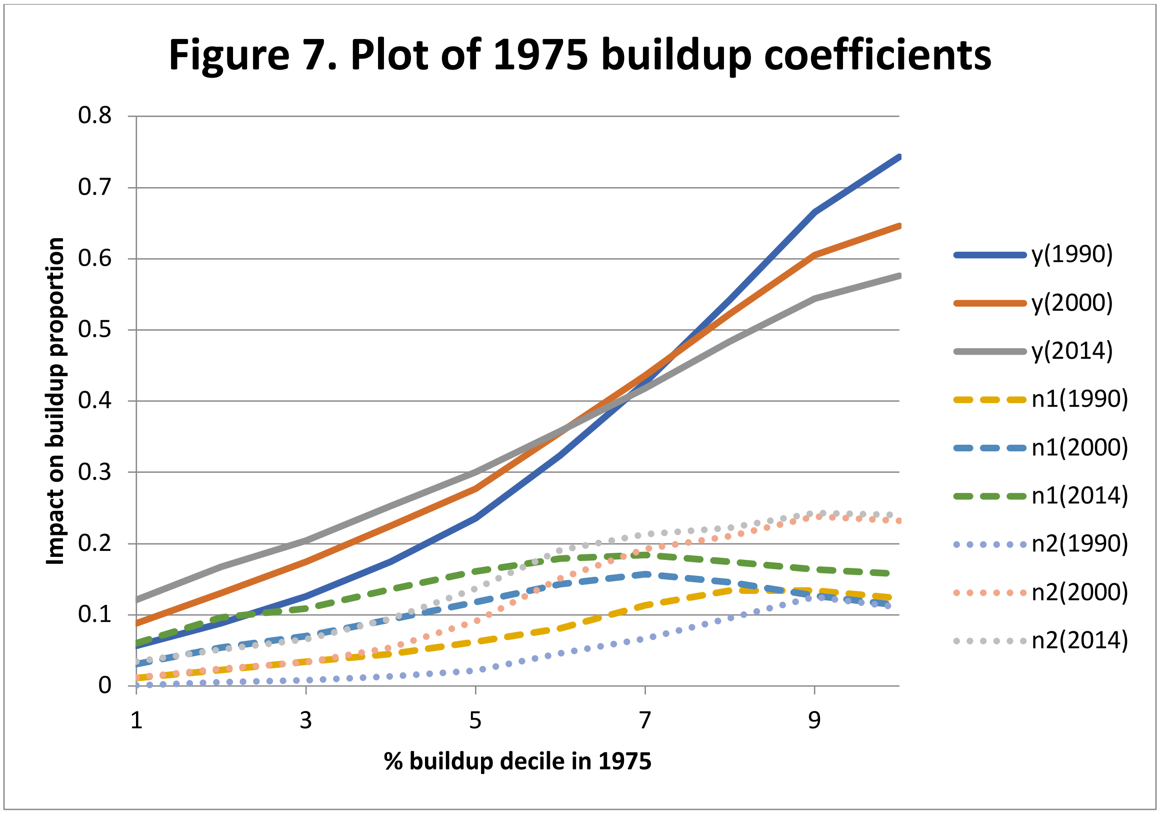

Notes: Figure 7 summarizes the coefficients from three separate regressions. The dependent variable in the three regressions is the proportion of a cell that is built up in 1990, 2000, and 2014 respectively. Each regression includes the 1975 values of buildup in the cell and in the neighboring cells n1 and n2. Each of the three 1975 buildup variables appears in the regression as a series of 10 dummies, one per buildup decile. The Figure shows the estimated decile coefficients in each of the three regressions for (1) 1975 buildup in the cell [lines y(1990), y(2000) and y(2014)]; 1975 buildup in neighboring cells n1 [lines n1(1990), n1(2000), and n1(2014)]; and 975 buildup in neighboring cells n2 [lines n2(1990), n2(2000), n2(2014)]. Each regression also includes all the road presence dummies for 1976 (see Figure 8) and the following time invariant regressors: longitude, latitude, and their product [three variables]; soil humidity and soil quality dummies [six variables]; and dummies for the presence of a water body, a land border, or the sea in the cell, the neighboring cells n1 and the neighboring cells n2, respectively [9 variables]. The number of observations in each regression is just shy of 3.8 million. R-squares are 0.799, 0.711 and 0.582, respectively. All coefficients shown here are significant at the 1% or better.

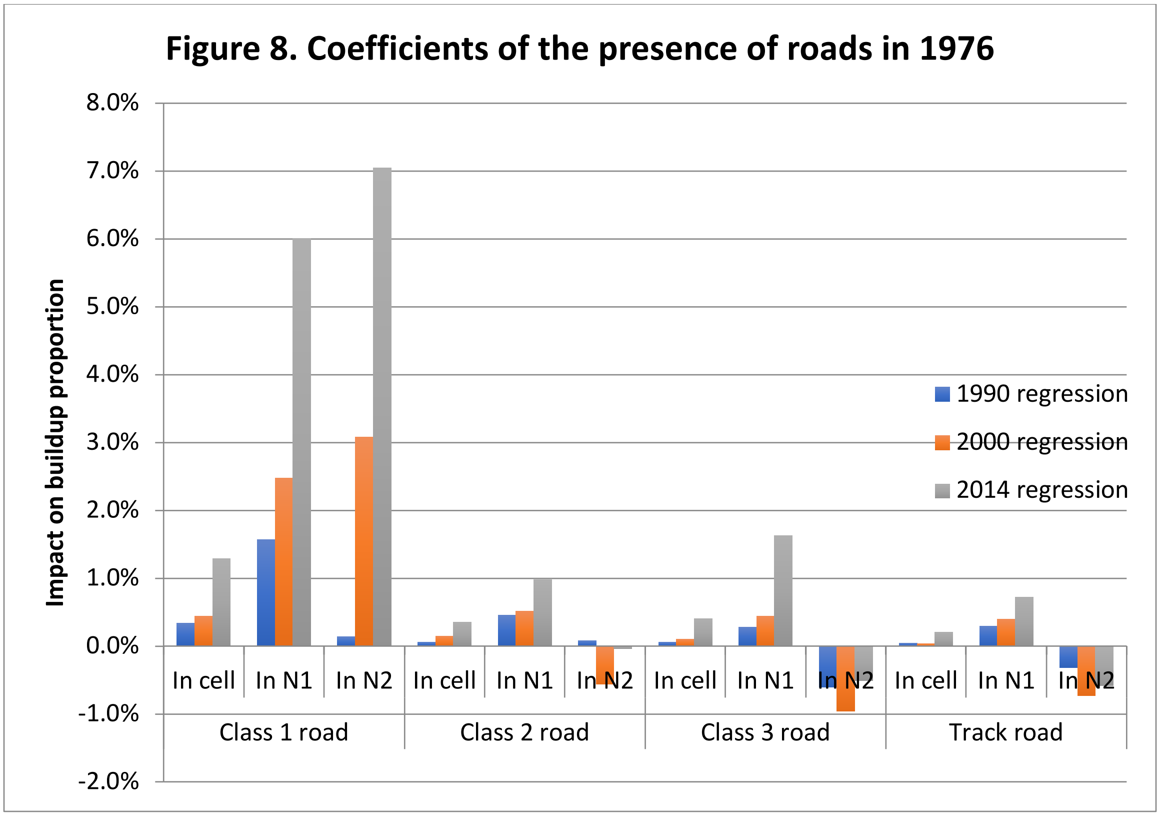

Notes: Figure 8 summarizes other coefficients from three separate regressions presented in Figure 7. The dependent variable in the three regressions is the proportion of a cell that is built up in 1990, 2000, and 2014 respectively. The Figure shows the coefficients of the presence in 1976 of a road of a certain class in the cell, the neighborhood N1 and the neighborhood N2. The road quality ordering is: class 1 (top category), class 2, class 3, and track (bottom category). Each regression includes the 1975 values of buildup in the cell and in the neighboring cells n1 and n2. Each of the three 1975 buildup variables appears in the regression as a series of 10 dummies, one per buildup decile (see Figure 7). Each regression also includes the following time invariant regressors: longitude, latitude, and their product [three variables]; soil humidity and soil quality dummies [six variables]; and dummies for the presence of a water body, a land border, or the sea in the cell, the neighboring cells n1 and the neighboring cells n2, respectively [9 variables]. The number of observations in each regression is just shy of 3.8 million. R-squares are 0.799, 0.711 and 0.582, respectively. All coefficients shown here are significant at the 1% or better.

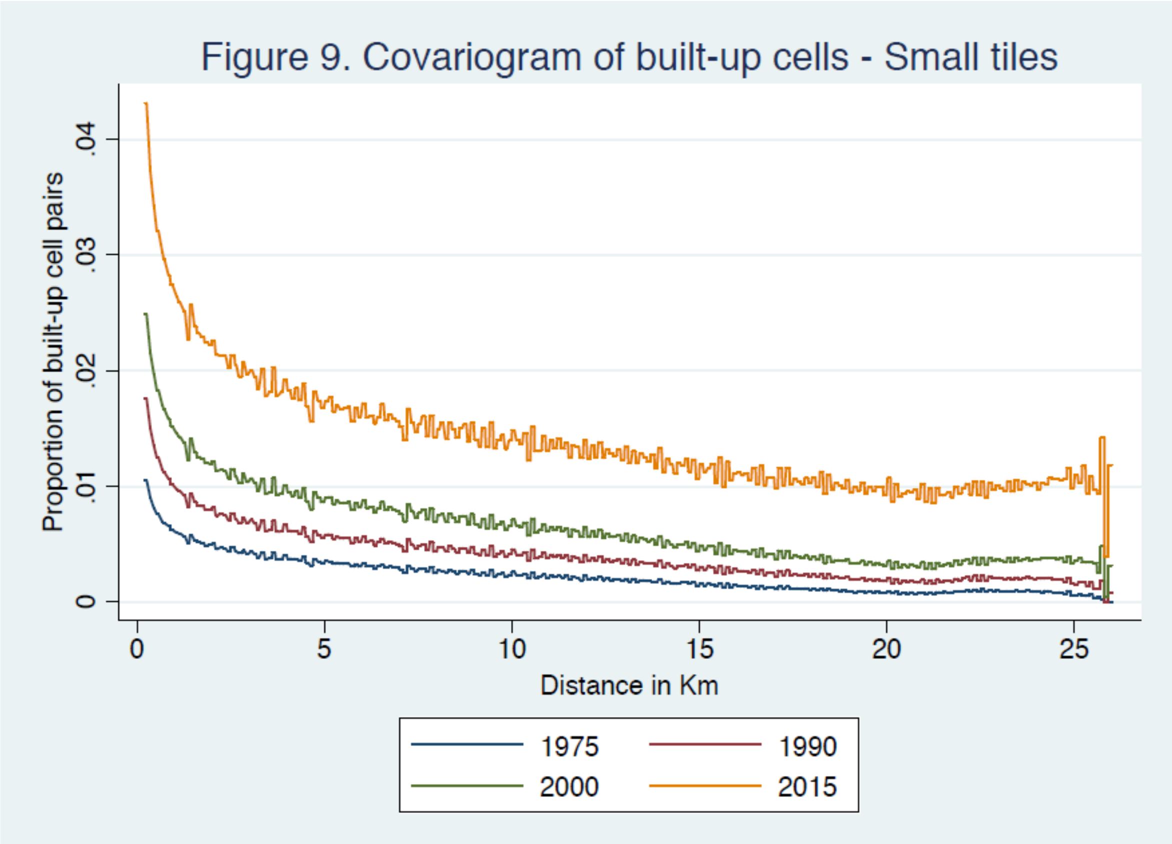
Figure 9. Covariogram of built-up cells - Small tiles
Proportion of built-up cell pairs
0
.01
.02
.03
.04
0
5
10
15
20
25
Distance in Km
1975
1990
2000
2015

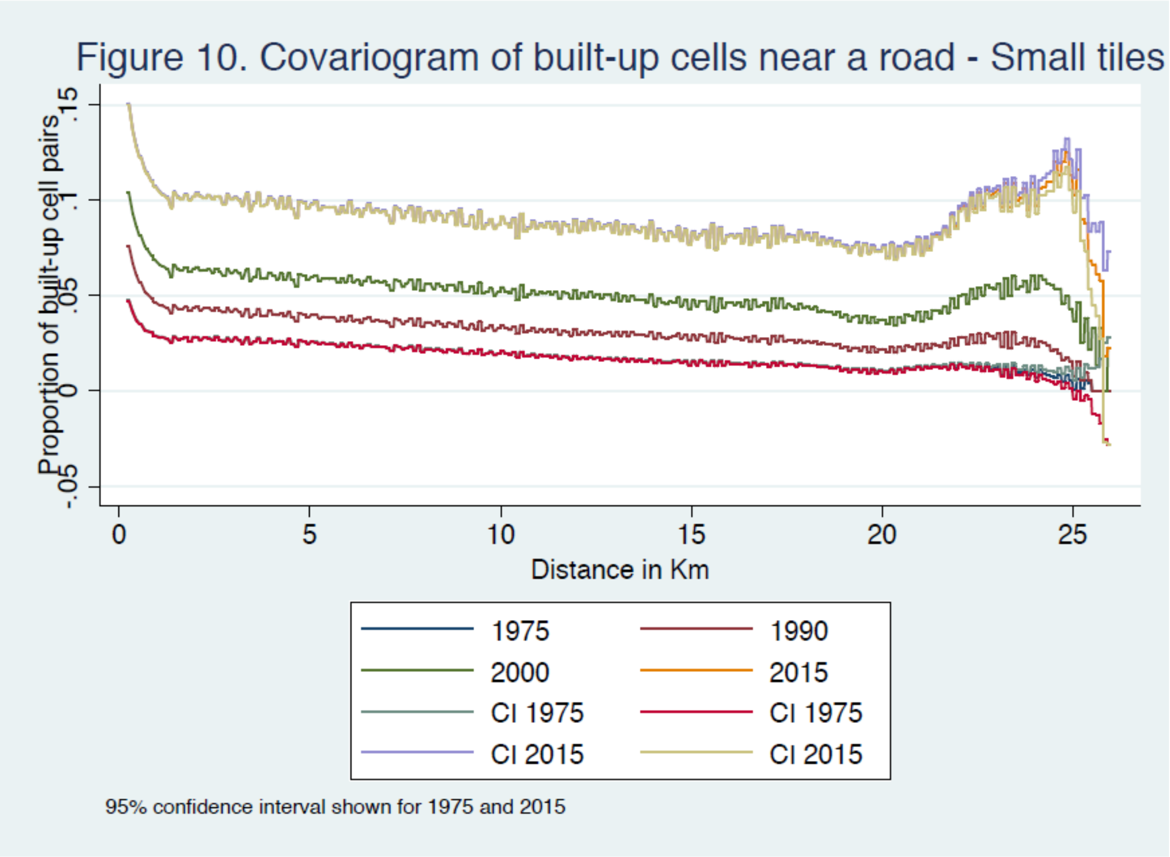

Notes: Figures 9 and 10 show, for each distance value within small tiles, the proportion of cell pairs that are both built-up. For instance, a value of 2% at distance 4Km means that pairs with two built-up cells make up 2% of all the cell pairs distant from each other by 4Km. For correlograms are displayed, one for each of the years for which we have buildup data. The two Figures are constructed in the same way, except that Figure 10 only includes cell pairs in which both cells are near a road, either in the cell itself or in a neighboring cell N1 or N2. In Figure 10 we also show the 95% confidence interval for two of the years. The bounds of the confidence interval are only visible at long distances; for other values they are too tight to be visible on the graph.

Figure 11. Covariogram of built-up cells - Big tiles

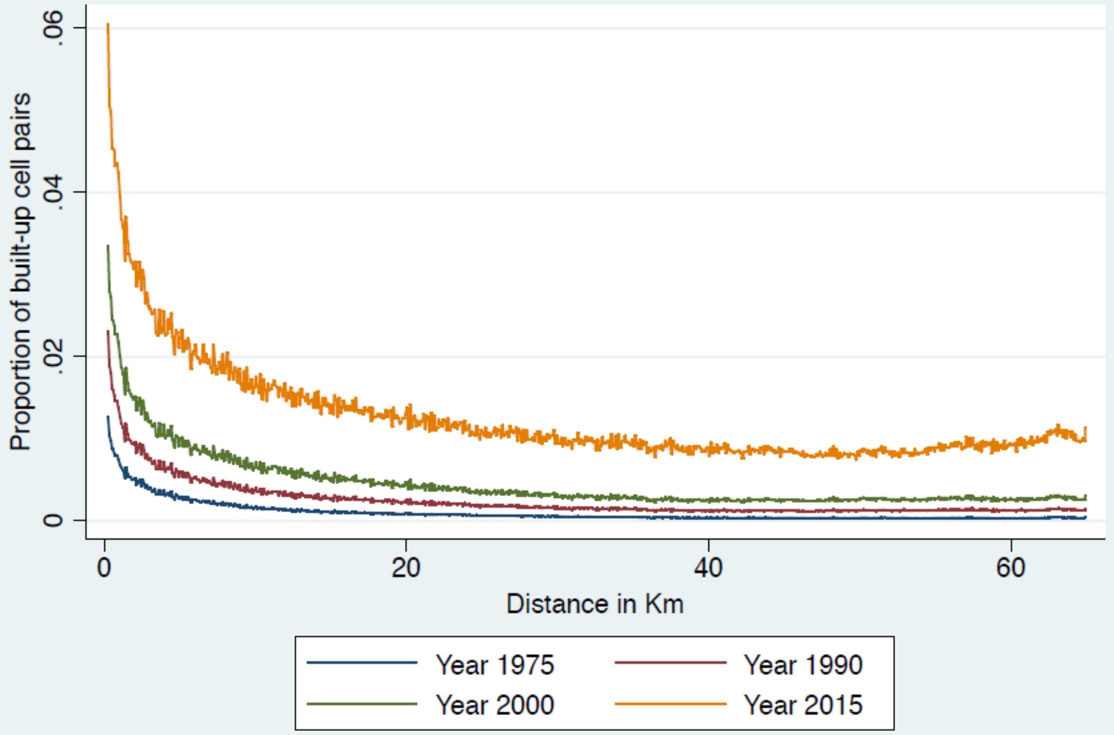

Figure 12. Covariogram of built-up cells near road - Big tiles

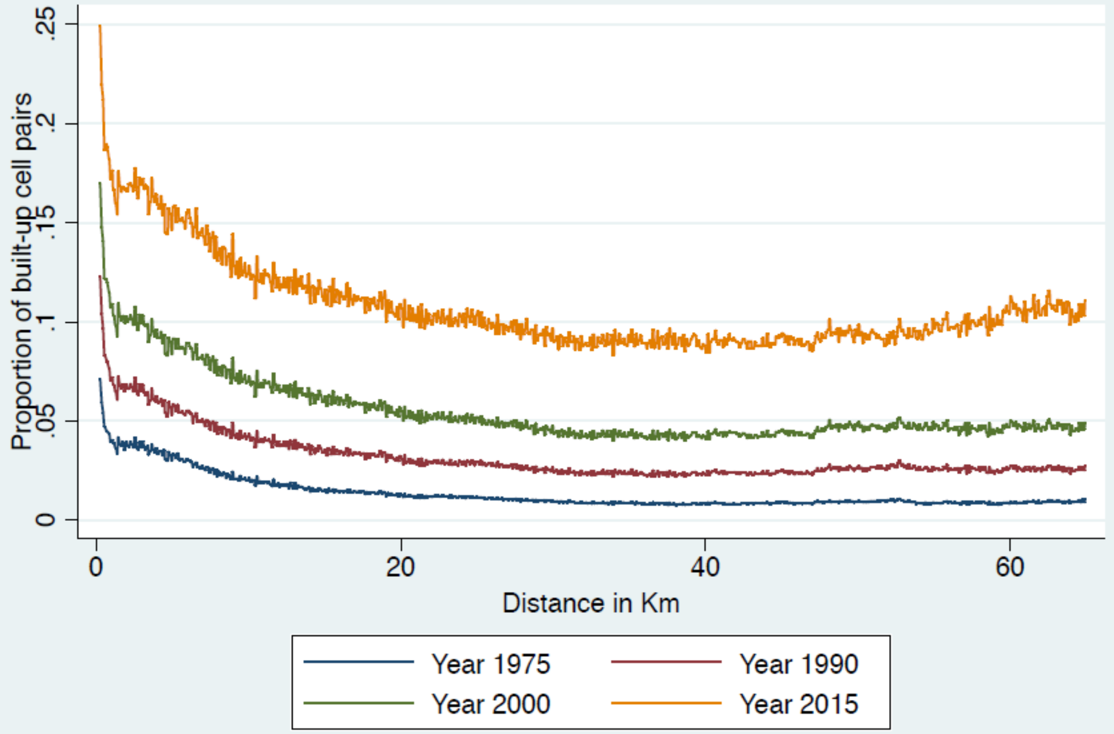

Notes: Figures 11 and 12 show, for each distance value within big tiles, the proportion of cell pairs that are both built-up. For instance, a value of 2% at distance 4Km means that pairs with two built-up cells make up 2% of all the cell pairs distant from each other by 4Km. For correlograms are displayed, one for each of the years for which we have buildup data. The two Figures are constructed in the same way, except that Figure 12 only includes cell pairs in which both cells are near a road, either in the cell itself or in a neighboring cell N1 or N2. Confidence intervals are not shown because they are too tight to be visible in the graph.

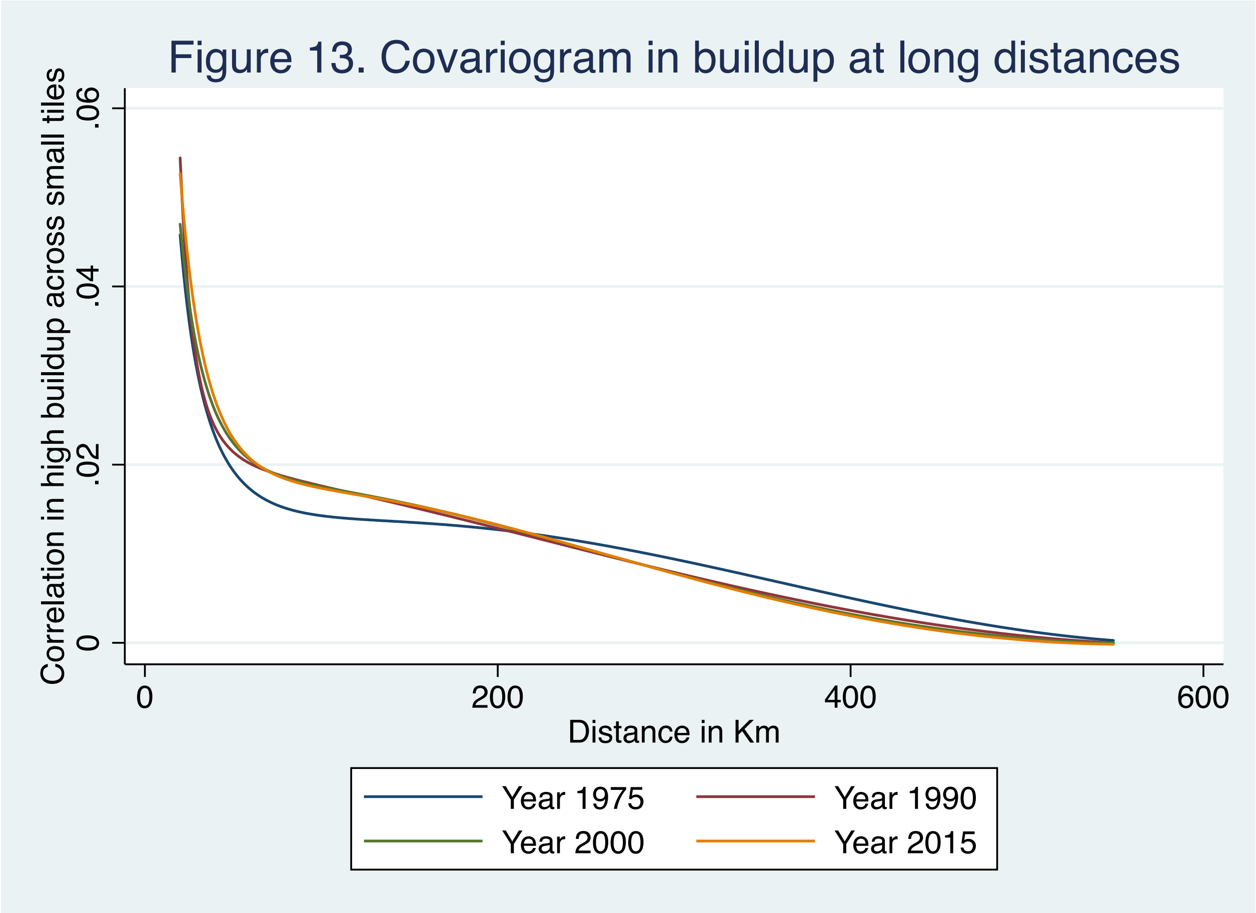

Notes: Figure 13 shows, for each distance value between small tiles, the proportion of tile pairs that are both in the top buildup decile. For instance, a value of 2% at distance 50Km means that tile pairs that are both in the top build decile make up 2% of all the cell pairs distant from each other by 50Km. For correlograms are displayed, one for each of the years for which we have buildup data. The four curves show, for each of the data year, the predictions from a fractional polynomial regression of the share of top decile pairs on distance.

Appendix A. Figures and Table accompanying the Conceptual Section

Figure A1. Low Density Settlement

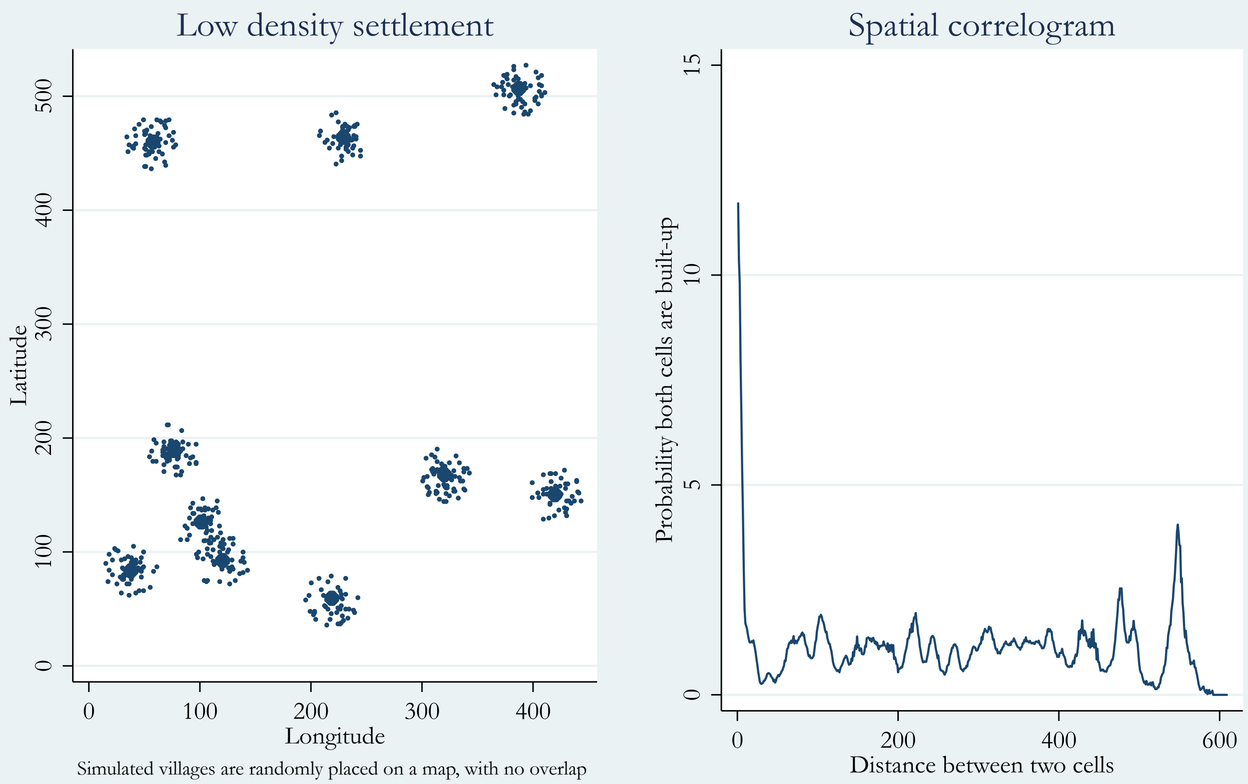

The graph on the right shows the spatial correlogram corresponding to the geographical pattern on the left.

Figure A2. High Density Settlement

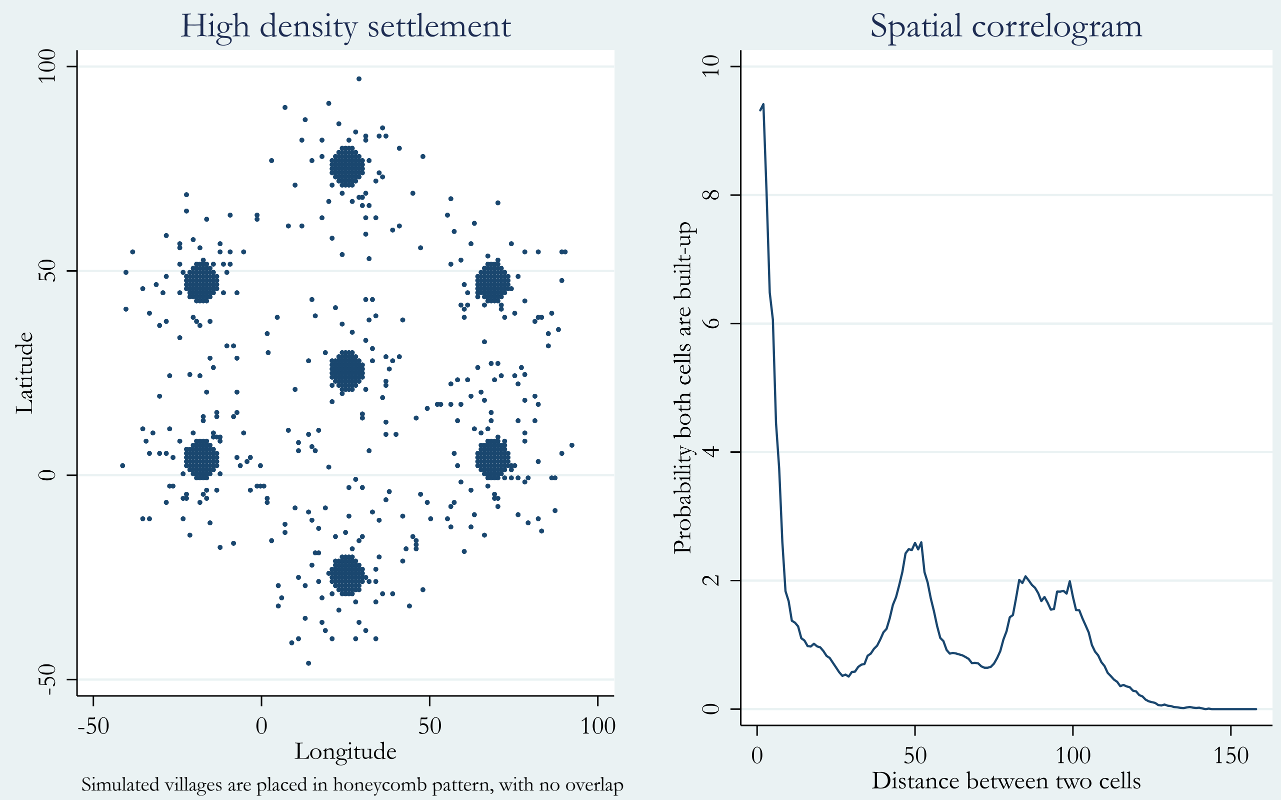

The graph on the right shows the spatial correlogram corresponding to the geographical pattern on the left.

Figure A3. Urban Growth Squeezing its Hinterland

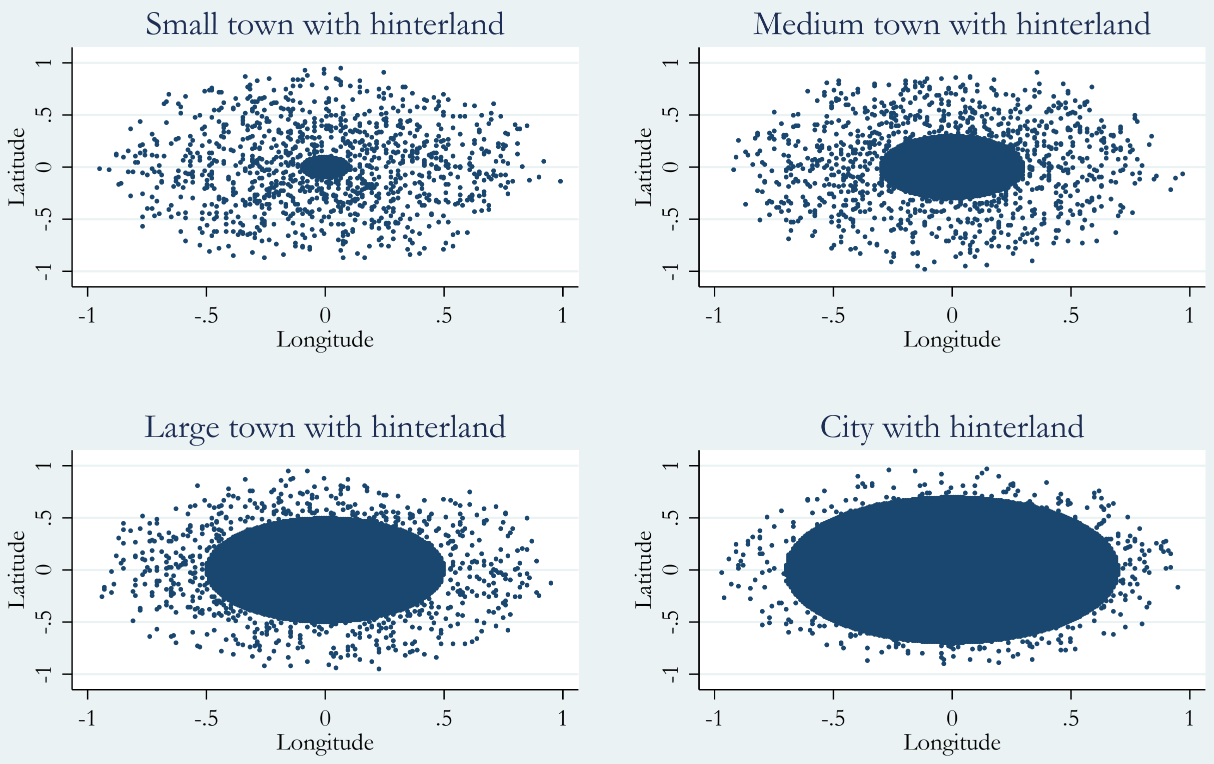

Simulated buildup density. Each dot represents a building. Fully built-up town center is fully opaque.

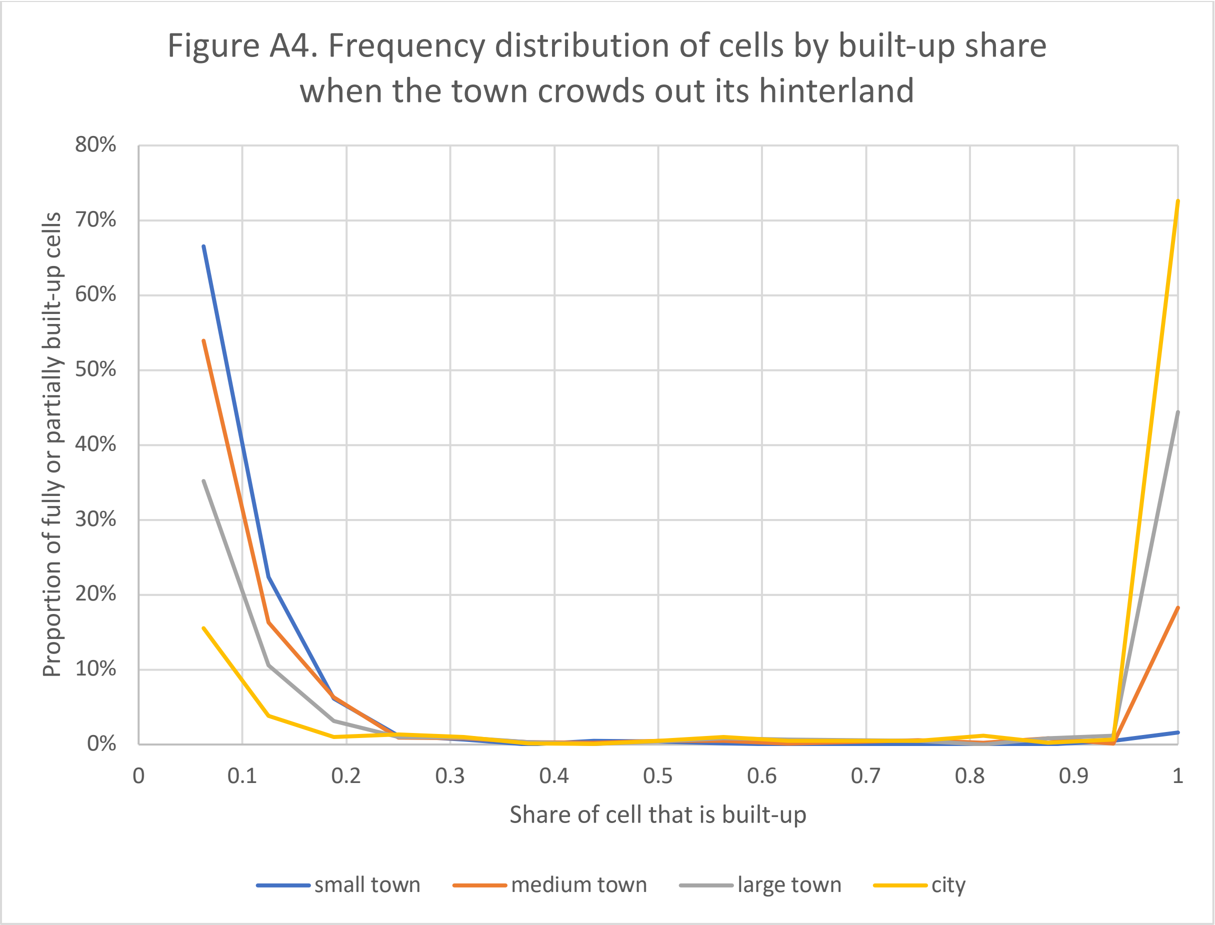
Figure A4. Frequency distribution of cells by built-up share
when the town crowds out its hinterland
80%
70%
60%
50%
40%
30%
20%
10%
0%
Proportion of fully or partially built-up cells
0
0.1
0.2
0.3
0.4
0.5
0.6
0.7
0.8
0.9
1
Share of cell that is built-up
small town
medium town
large town
city

Appendix B. Checking whether Zipf Law applies to partial buildup

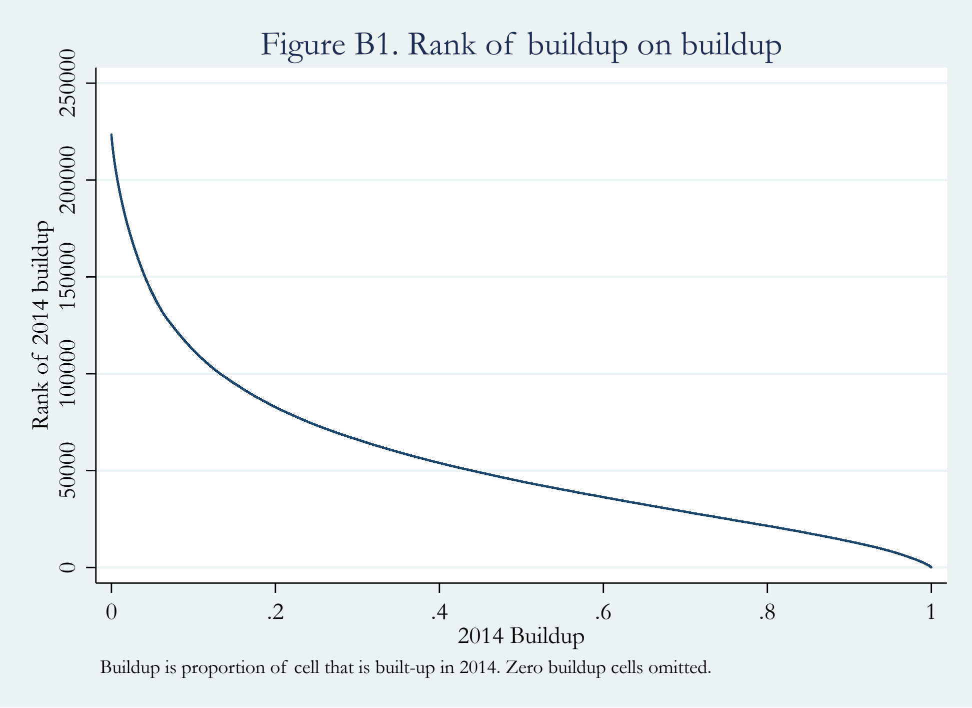

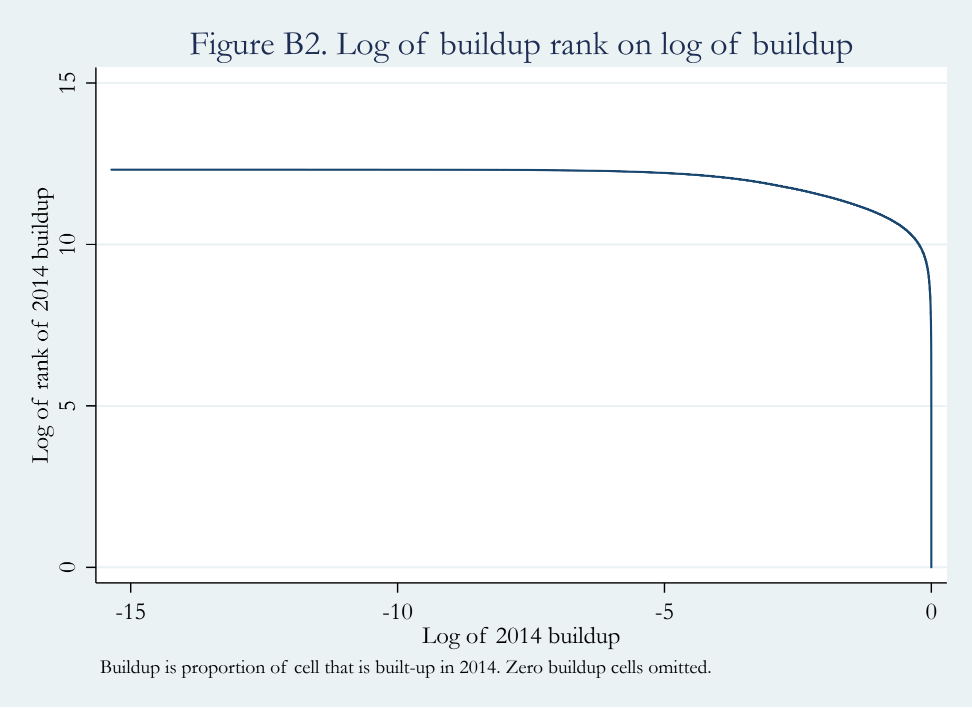

Notes: Figure A1 shows a non-parametric regression line of the rank of a cell in terms of buildup on its buildup in level. Figure A2 shows a non-parametric regression line of the log of the rank of a cell in terms of buildup on the log of its buildup. All cells with zero buildup (the majority) are omitted from these regressions. Each non-parametric regression is estimated using a fractional polynomial

Appendix C: Ghana

Ghana is a West African country located on the Gulf of Guinea, only a few degrees north of the Equator. Historically, the country was one of the prosperous countries in Africa because of its rich natural resources, particularly gold. The abundance of gold attracted Portuguese, Dutch, English, and French traders to the country from the 15th century. Beginning in the 17th century — in addition to the gold trade —European traders also participated in the Atlantic slave trade in this area. After prolonged wars between British colonists and Ghana's Ashanti kingdom, much of the territory around Ghana was brought under British colonial rules during early 1900s. In 1957, four British colonies (Gold Coast, Ashanti, the Northern Territories and British Togoland) were unified to form the independent country of Ghana.

The landscape of Ghana is characterized by low physical relief with its highest elevation about 880m above sea level. The country has five distinct agro-ecological zones (Figure A1.1). The coastal savannah ranges around its 537 km of coastline. A tropical rain forest belt, broken by heavily forested hills and many streams and rivers, extends northward from the shore, near the Ivory Coast frontier. The general terrain in the northern and northwestern part of Ghana outside the Volta Basin consists of a dissected plateau, which averages between 150 and 300 meters in elevation and, in some places, is even higher. This region is home to a vast upland savannah. The region surrounding the largest man-made lake Volta accounts for about 45 percent of Ghana's land area. This region extending from deciduous forest in the south to savannah in the north remains poor in terms of road infrastructure. The diversity of agro-ecological zones and terrain resulted in differences in agricultural practices and crops as well as human settlements.

Figure A1.1: Main Agroecological zones of Ghana

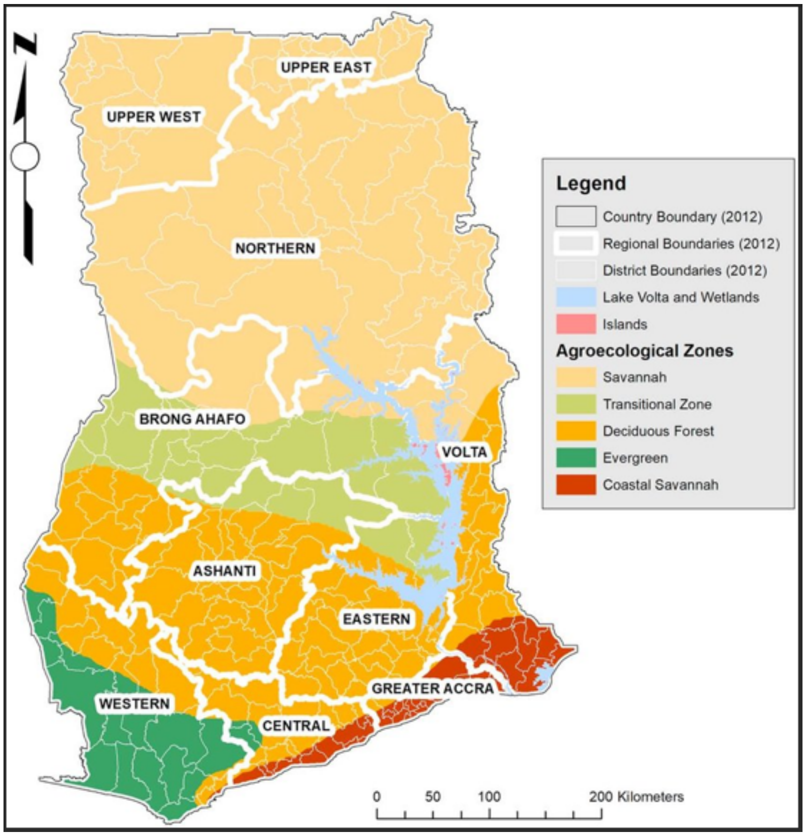

Ghana is currently classified as a lower middle-income country with a GDP per capita of $5637 at purchasing power parity in 2019 (World Bank). With a land area of 238,534 square km, Ghana is comparable to US state of Oregon in terms of land size. Ghana's population increased from 6.6 million in 1960 to 30 million in 2019. As a result of rapid population increase, population density increased from 28/sqkm in 1960 to 130/sqkm in 2019. Figure A1.3 maps the regional differences in population density in Ghana. Population density is highest in Greater Accra region, followed by central, Ashanti, and Volta regions.

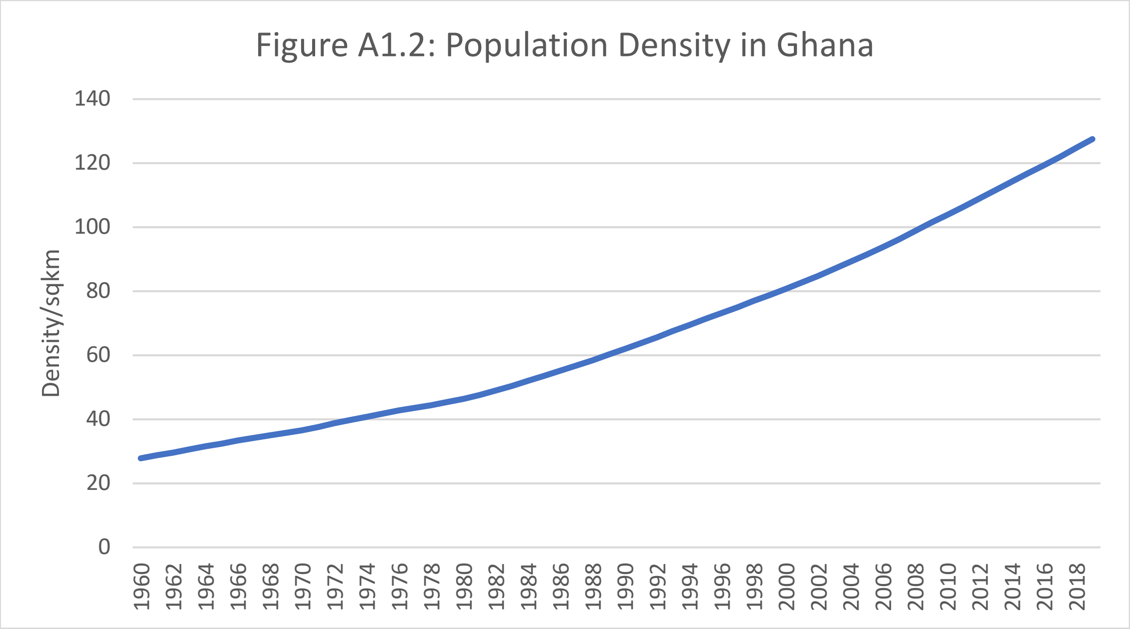

Source: WDI

Figure A1.3: Population density of Ghana by regions

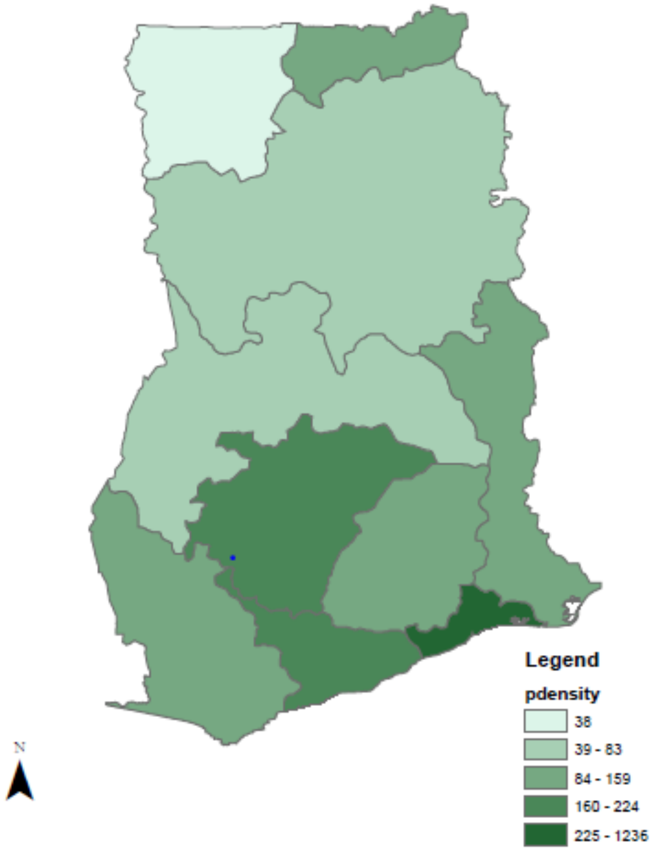

Source: Census 2010

From mid 1980s, Ghana experienced rapid increase in its urban population. In 1975, about 30% of Ghana's population was urbanized which increased to 32% in 1984. By 2019, the urban share of total population increased to 57 percent, making it as one of the most urbanized African countries. All regions of the country have experienced steady urbanization. In 2000, Ghana was a country of a few limited metropolitan areas and many small towns. Since then, all city types have dramatically increased in number, and Ghana has experienced faster urban population growth in its smaller cities than its larger ones. The number of medium (20,000–50,000 people) and large medium (50,000–100,000) sized towns has quadrupled and tripled, respectively. In 2000, there were only nine towns with population between 50,000 and 100,000; by 2010 the number had quadruped to 36. Ghana has two cities with more than a million of population: Accra and Kumasi. While Accra has grown considerably, its urban primacy has diminished: its 24.4 percent share of the total urban population in 1984 declined to 16.6 percent by 2010, representing more balanced urban growth that has moved from Accra alone to Accra plus Kumasi, port cities, and smaller cities (World Bank, 2015).

Figure A1.4: Main cities and their population

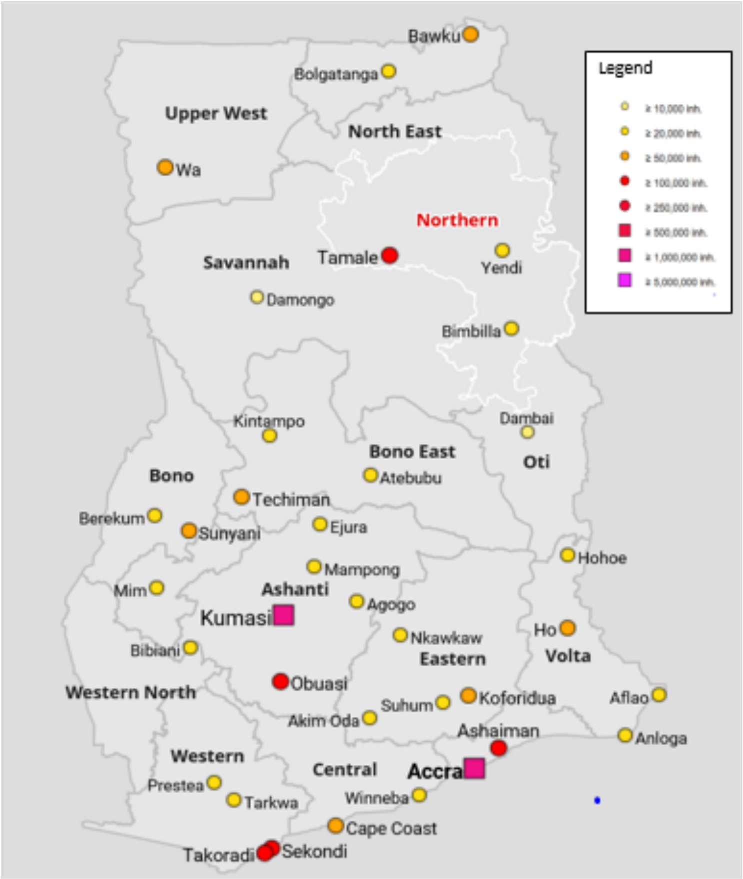

Source: https://www.citypopulation.de/en/ghana/cities/

The rapid urbanization in Ghana is also associated with structural transformation, economic growth and poverty reduction. According to 1970 census, more than three-quarters of its population was engaged in agriculture, animal husbandry and fishing which declined to 50% in 2010. By 2019, half of Ghana's workers are employed in services and another 20% in industry (Figure A1.5). Three main exports from Ghana are gold (7th largest producer), cocoa (second largest) and timber. Only recently, employment in industry has picked up as Ghana moved into more modern manufacturing. During this period of rapid urbanization, Ghana also experienced robust growth of GDP in recent years. From 1961 to 1983, GDP growth averaged 0.9 percent annually, from 1984 to 2013 it averaged 5.7 percent annually, and from 2005 to 2013 GDP growth averaged 7.8 percent (World Bank, 2015). Rapid economic growth has resulted in a reduction in poverty in both rural and urban areas, with the total poverty incidence dropping below 25 percent in 2013 from 56.5% in 1992. Rural poverty incidence still is much higher (37.9%) than in urban areas (10.6%).

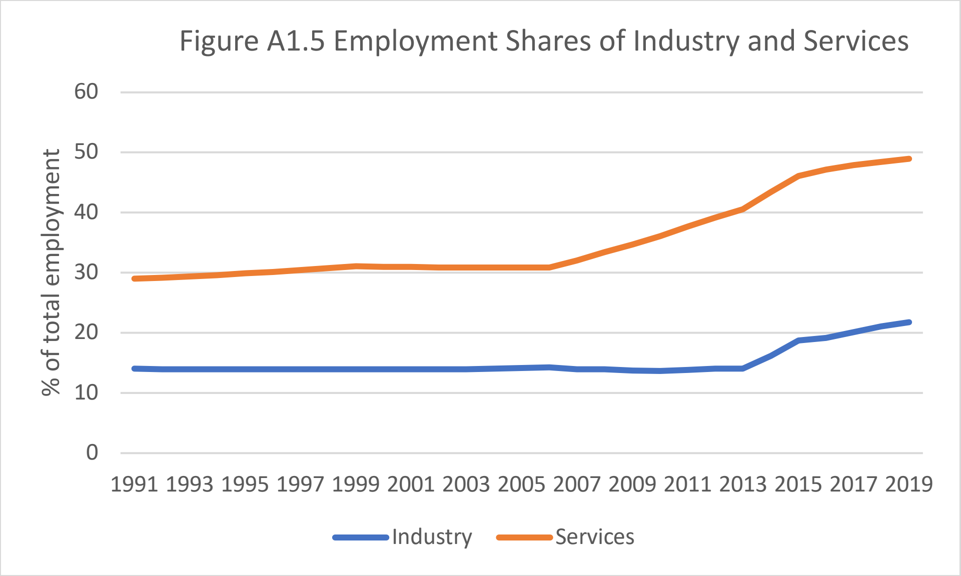

Source: WDI

References

World Bank Group. 2015. Rising through Cities in Ghana: Ghana Urbanization Review Overview Report. World Bank, Washington, DC. https://openknowledge.worldbank.org/handle/10986/22020

Appendix D: Data Sources

Global Human Settlement Layer (GHSL): These datasets that have been put together by the Joint Research Centre of European Commission and is our main data source. The GHS-BUILT data, a part of the GHSL datasets, contain a multitemporal information layer on built-up presence as derived from Landsat image collections (GLS1975, GLS1990, GLS2000, and ad-hoc Landsat 8 collection 2013/2014). The methodology for construction of these datasets is described in Pesaresi et al (2016). The dataset provides built-up information at three resolutions: 38m, 250m and 1km. At 38m resolution, the dataset identifies presence or absence of built-up in a 38X38 m^2 which is then aggregated at 250km and 1km resolutions. The details of this dataset can be found in https://ghsl.jrc.ec.europa.eu/index.php. In Figure A.2 below, we plotted the extracted data for Ghana and for the city of Kumasi.

Figure A2.1: Built-up Density in Ghana (left) and city of Kumasi (right) between 1975-2014

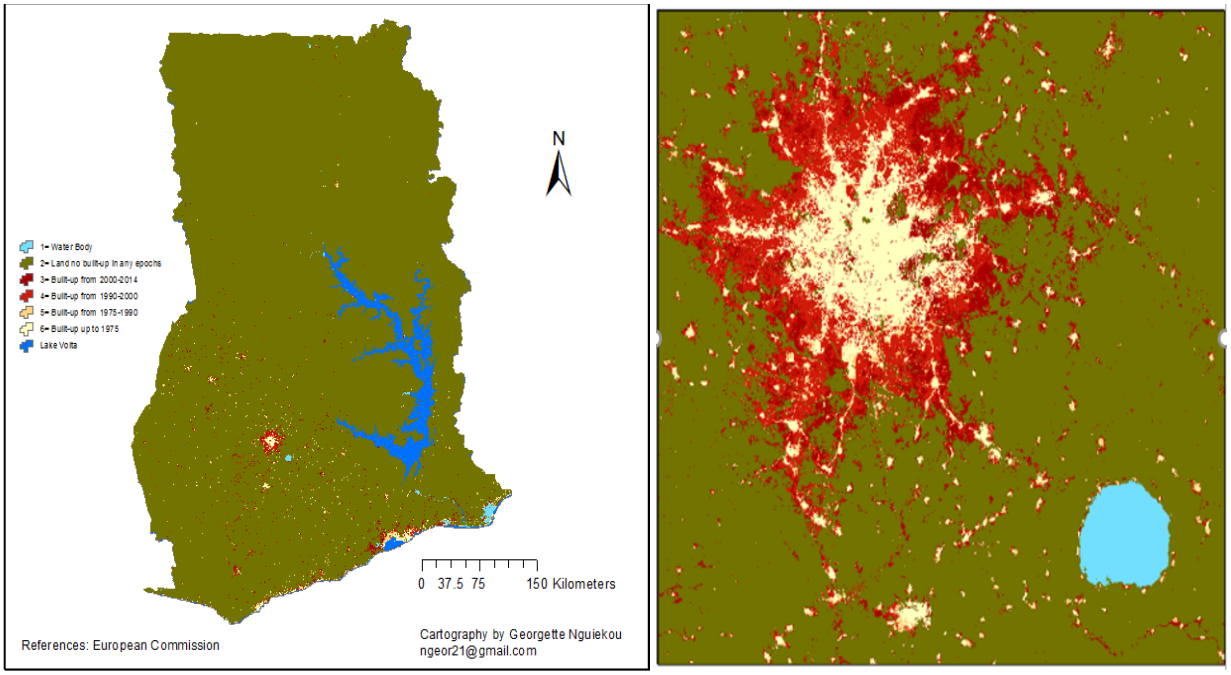

To extract built-up information for Ghana, we overlay Ghana shapefile over GHSL dataset. For each 250X250m cell, we extract the proportion built in 1975, 1990, 2000 and 2014 to form a panel dataset at cell level. These cell coordinates are then fixed to extract and merge in other data using spatial join function. The GHSL dataset is also used to find whether a cell belongs to water body (e.g. lake Volta). The data extraction is done by a program written in R.

Ghana Shapefiles: These files are obtained from the Ghana Statistical Services. The shapefiles are used to determine international it borders. We also extract information on whether a GHSL cell falls on international border or sea border from the shapefiles.

Global Agro-Ecological Zones: The data for agro-ecological zones are downloaded from the GAEZ-Global Agro-Ecological Zones data portal which was developed by the Land and Water Division of the Natural Resources Management and Environment Department of FAO. The resolution for GAEZ dataset is 10 km. We overlay 250 by 250 meters grids of GHSL dataset over GAEZ dataset to extract dominant soil types for our panel of cells. The dataset can be downloaded from

https://climate-adapt.eea.europa.eu/metadata/portals/gaez-global-agro-ecological-zones. The main soil categories are steep terrain, arctic/cold, desert/arid, irrigated soils, hydromorphic soils, dry (good, moderate, poor), moist (good, moderate, poor), semi-humid (good, moderate, poor), humid (good, moderate, poor) and water. For each cell, we define a dummy variable to indicate the dominant soil type. Figure below shows the map of soil types in Ghana.

Figure A2.2

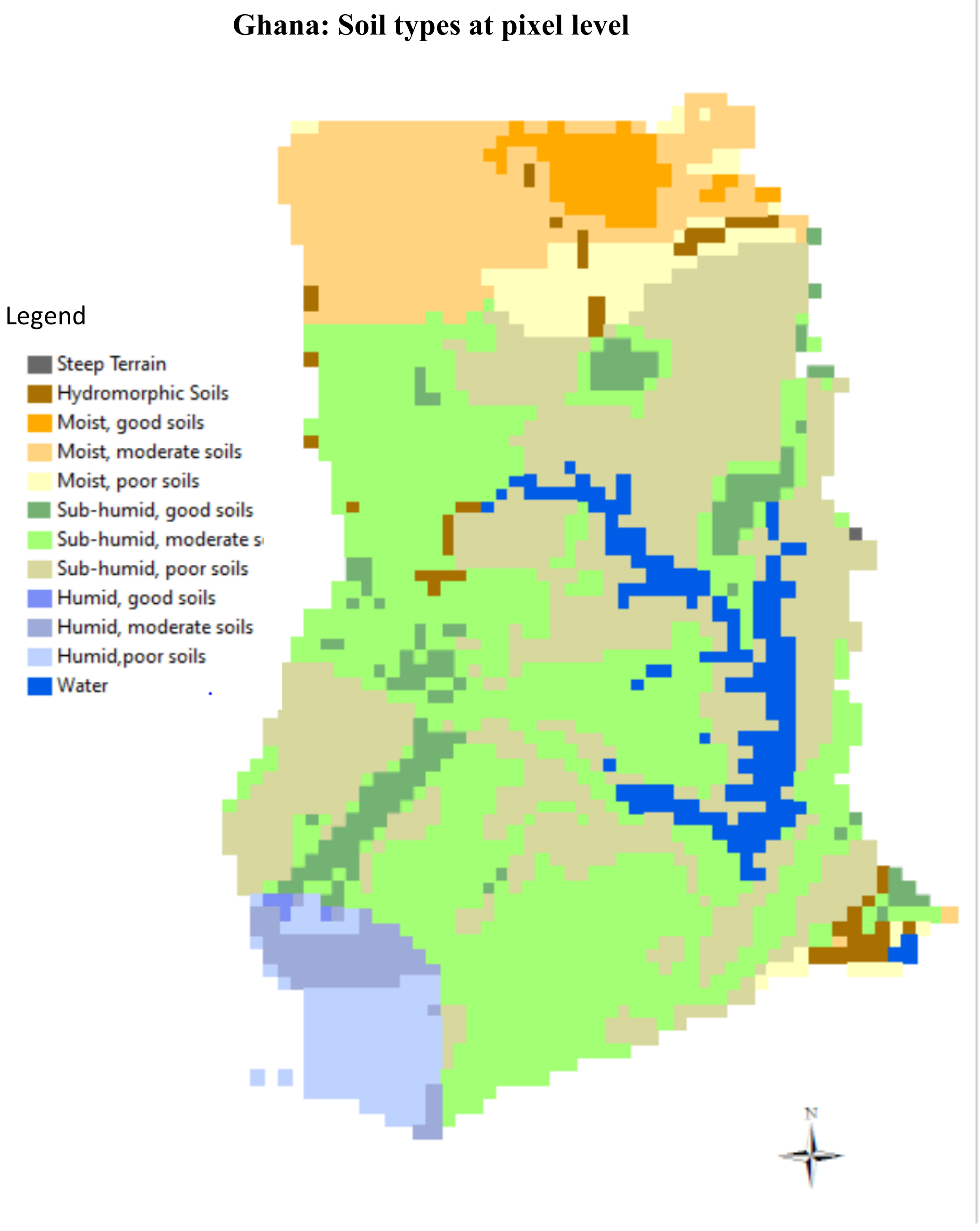

Ghana Road networks: The road network data comes from two sources. Road network for 2008 is derived from the data produced by the Africa Infrastructure Country Diagnostic (AICD) (World Bank, 2008) and our own digitization effort. For 1976 and 1986, we digitized high resolution road maps from censuses. Below are maps of road networks in 1976 and 1986. We overlay road maps on GHSL data to determine if a cell had road in it either in 1976 or 1986 or both.

Figure A2.3: Ghana Road network over time

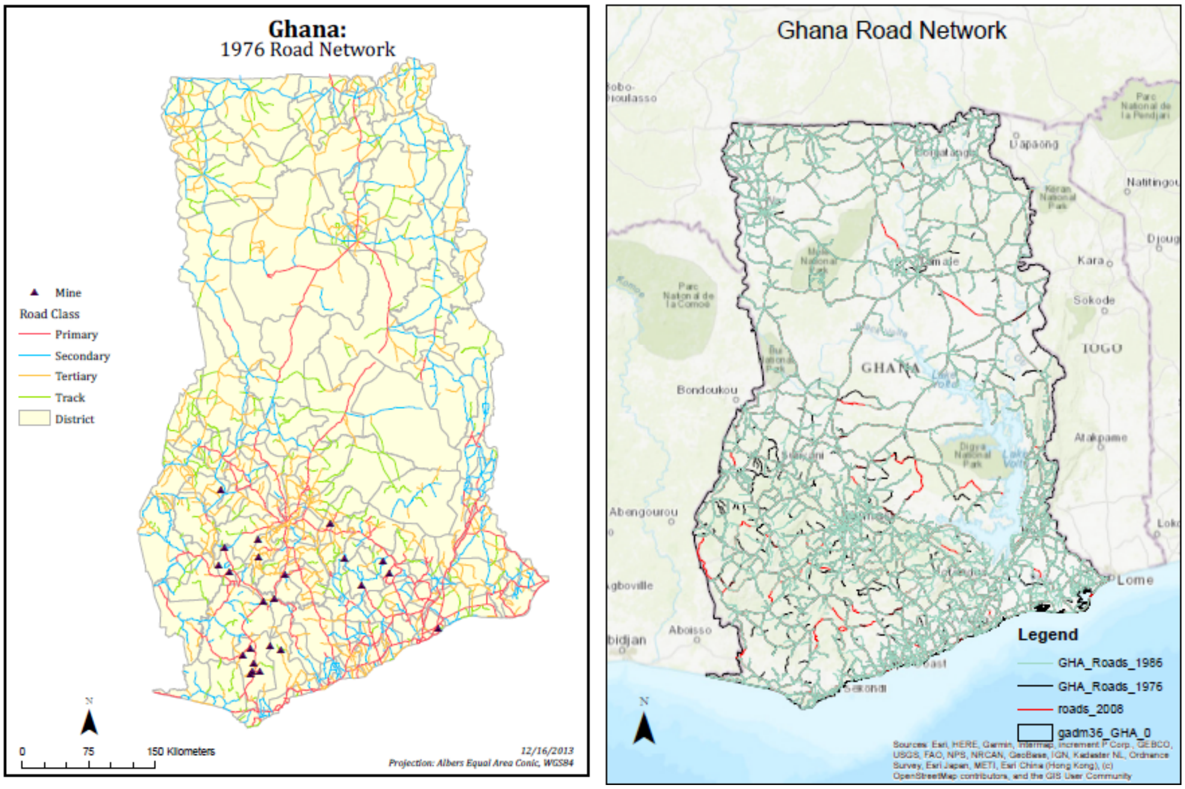

References

Gwilliam, Ken, Vivien Foster, Rodrigo Archondo-Callao, Cecilia Briceño-Garmendia, Alberto Nogales, and Kavita Sethi, *Africa Infrastructure Country Diagnostic: Roads in Sub-Saharan Africa,* World Bank, 2008.

Pesaresi M., Ehrlich D., Ferri S., Florczyk A.J., Freire S., Halkia S., Julea A.M., Kemper T., Soille P. and V. Syrris, Operating procedure for the production of the Global Human Settlement Layer from Landsat data of the epochs 1975, 1990, 2000, and 2014. Publications Office of the European Union, EUR 27741 EN, 2016. doi: 10.2788/253582.

THE FREEMAN SPOGLI INSTITUTE FOR
INTERNATIONAL STUDIES AT STANFORD UNIVERSITY

Stanford, September 30, 2020

Dear Sir/Madam,

In response to an invitation to submit from Diego Rybski, I am submitting my paper with Forhad Shilpi entitled "The Evolution of Built-up Areas in Ghana since 1975" for possible publication in Cities as Complex Systems PLOS ONE Collection. The paper is not currently under consideration by any other journal.

An earlier version of the paper was submitted in June of this year and was assigned the number PONE-D-20-17761. It received a 'reject-and-resubmit' verdict in July 2020. This resubmission is accompanied by a detailed response to the comments received from the original editor and referees. Thanks for your consideration.

Sincerely,

Marcel Fafchamps

Senior Fellow

Center for Democracy, Development, and the Rule of Law

Response to the Editor
[original text in roman font, response in italics]

PONE-D-20-17761
The Evolution of Built-up Areas in Ghana since 1975
PLOS ONE

Thank you for submitting your manuscript to PLOS ONE. After careful consideration, we have decided that your manuscript does not meet our criteria for publication and must therefore be rejected.

If however, you are able to address the reviewers' concerns through revisions to your manuscript, we may be willing to consider a resubmission of your work. The revised manuscript should be submitted as a new submission to PLOS ONE and be accompanied by a cover letter that refers to the original submission number and details how the revisions have been completed. I am sorry that we cannot be more positive on this occasion, but hope that you understand the reasons for this decision.

Thank you for allowing us to revise and resubmit our paper as a new submission. We attach below a detailed response to the changes we have brought to the paper in response to the referees' comments. We believe we have adequately addressed them all, to the extent allowed by the data. Thanks for these comments. We feel that the paper is much improved as a result.

Response to Reviewer 1

The paper is purely a description of so-called the evolution of built-up areas in Ghana. I cannot see any theoretical/conceptual/methodological/practical contribution from this paper.

Thanks for pointing this out. We have written a completely new conceptual framework section, for which we have created several new Figures that illustrate how our analysis relates to the framework. These Figures also clarify the methodological contribution made by the paper. In addition, we have moved the conceptual framework to Section 2, brought it up directly into the introduction, and linked our findings to it in the conclusion. We believe these important changes make the contribution of the paper much clearer. Thanks for nudging us in that direction.

The introduction does not tell us why we have to understand this research.

We have taken this point to heart and entirely rewritten the introduction to better explain to the reader the motivation behind our project and the reasons for the choices we made.

There is no critical review of the existing literature. Readers are left with no idea about the knowledge gap into which this paper is intended to fill. There exists no dialogue between this paper and the existing studies.

Good point, related to the discussion above. Partly this is due to the fact that much of the current literature focuses on big cities, while we focus on all human settlements, including small rural ones. But it is fair to say that we could have done a better job of linking our work to the rest of the literature. This has now been corrected. We have also added quite a few references to the current literature, helping the reader better realize how our paper fits in the present literature.

The conclusion of the paper reads like the outcome of a consultation report.

Ouch! We did not want to oversell our results. But the referee is right to point out that we can do better. The conclusion has been entirely rewritten to read more like an academic paper. For the record, this paper was never part of a consulting project. It was a research project from the start.

Response to Reviewer 2

The authors used high resolution satellite data to study evolution of human settlement in Ghana over a 40 years period. The authors have done a lot of work to analyze the expansion of human settlement in Ghana from many aspects, and have obtained several valuable and meaningful findings. However, the quality of this manuscript is not enough for publication, there are still some areas that need to be supplemented and improved. Therefore, I recommend a major revision for this manuscript. Please see suggestions below which are not mandatory.

1. I suggest that the authors re-write the abstract because it has a high repetition rate with the conclusion. Add 1-2 sentences to clarify the importance and significance of this study and let readers know why you did this research. In addition, it is necessary to briefly introduce the method of this study.

The abstract has been corrected to eliminate duplication with the conclusion, better motivate the study, and introduce the methodology. The conclusion itself has been entirely rewritten to better explain how our findings can be interpreted in the light of the newly rewritten conceptual section, which now plays more prominently in the introduction and conclusion.

2. The authors point out that the purpose of this paper is to document how the spatial distribution of buildings has evolved. The authors need to re-explain the main purpose and significance of this study.

This comment complements those of the first referee, and confirms that we did not do a good enough job motivating the study, and explaining its purpose and significance. The introduction has been fully rewritten to address these shortcomings, and to build a better link between the paper and the existing literature.

3. The major scientific contribution of the work is not clear. Although the authors point out that this paper contributes to the literature on urbanization and regional development, I think the contribution and innovation of the paper are not accurate and specific.

This weakness too is related to the comments from Referee #1. We needed to clarify from the start what our conceptual framework was -- it was discussed in section 3 but it was not brought enough to the fore in the rest of the paper. This has now been corrected. We have also moved the conceptual section to the second position, before introducing the data, and the section itself has been completely rewritten. Several Figures have been added that clarify the methodological contribution made by the paper. In addition, we have introduced the conceptual framework directly into the introduction, and linked our findings to it in the conclusion. We now do a much better job a clarifying what the anticipated and actual contributions of the paper are, and how they relate to the existing literature.

4. In the section of Introduction, the authors have shown too much research process, results or conclusions, I think it is not necessary, or the authors can make a brief overview. In my opinion,

the authors should pay more attention to introducing research background, literature review and scientific contribution in this section. Let readers know the purpose and significance of this study.

These are good suggestions. We have rewritten the introduction taking your recommendations into consideration. Thanks for the guidance.

5. In the section of Data, I think the authors should detail the sources and processing of the data in this study. It is necessary to add a section describing the methods. More details about the study area are also necessary, adding figures to show the data and study area.

Good point. We have expanded the main text to include more information about the way the human settlement data was generated, drawing from published data documentation. In addition to notes at the bottom of the Tables and Figures, an online appendix has been included that details the way the data were manipulated to generate the various graphs and tables in the paper. We also provide a bit more contextual information about Ghana in the paper, and include a short online Appendix for the reader unfamiliar with the country.

6. In the section of Empirical results, the authors both showed the results and discussed them. Therefore, I think the title of this section should be changed to “Empirical results and discussion”.

Done.

7. In this current version of the manuscript, I did not see the Appendix Figures A1 and A2.

The two omitted figures have been added. Sorry about that.

8. In the maps at the end of the paper, the maps 1e, 1d, 2e, and 2f are not marked with the order and name.

Sorry about that. This omission has been corrected.
