# Supplementary material for: The evolution of built-up areas in Ghana since 1975
Source: PLoS One. 2021 May 21;16(5):e0250847. doi: 10.1371/journal.pone.0250847 (PMC8139467; doi:10.1371/journal.pone.0250847)
Supplement: Attachment — Submitted filename: Responses_Editor_Reviewers_Revision.pdf [file pone.0250847.s004.pdf]

Response to the Editor – second revision

[original text in roman font, response in italics and blue shade]

Thank you for submitting your manuscript titled, 'The Evolution of Built-up Areas in Ghana since 1975' (Ref. No.: PONE-D-20-30587) to PLOS ONE. The manuscript has been reviewed. After careful consideration, we find that the manuscript has some merit with some interesting results, but it needs a major revision to address reviewers' comments and fully meet PLOS ONE publication criteria. You can find reviewers' comments at the bottom of this letter.

We invite you to submit a revised version of the manuscript. The changes required in the manuscript are very significant and require you to respond fully. We will send your revised manuscript for further external review. Therefore, we strongly recommend addressing concerns raised in full.

Thank you for allowing us to revise and resubmit our paper. We attach below a detailed response to the changes we have brought to the paper in response to your and referees' comments. We believe we have adequately addressed them all, to the extent allowed by the data. We attach the revised version of the paper as well as a version showing (in color) all the changes made to the text. We've also added detailed notes to Appendix Tables A1 to A4, as requested. All the other Tables and Figures already had detailed explanatory notes.

Be sure to address:

1. Restructure the manuscript, including a more convincing problem statement. One of the reviewers raised concern that the interpretation and contextualization of the work in housing policy and housing policy analysis in Africa seems weak. While the introduction reads well, it does not provide a gap in scientific knowledge that currently exists and why that gap needs to be filling?

We have restructured the paper to consolidate all conceptual issues under a new Materials and Methods section. We conducted a more thorough survey of literature and added 13 more references. The introduction was rewritten to highlight the contribution of this paper to the existing literature. With respect to housing policy, the paper cannot relate directly to housing conditions and policies since satellite data do not distinguish housing conditions: they only tell us whether a cell is built-up or not. However, with reference to Tusting et al. 2019, we mention in footnote 2 that while housing conditions in much of Africa improved considerably between 2000 and 2015, more than half of Africa's urban population still live in settlements with poor housing conditions.

2. Studies support using high-resolution imagery to study urban sprawl, in particular developing countries. Therefore, provide a convincing argument on using a coarse resolution data product that might have overestimated urban sprawl or even missed capturing a small extent of development. Repeat this study with high-resolution imagery for a few time steps and relate them with the corresponding coarse imagery products to show their accuracies.

We have clarified in the text that our paper is not trying to use high resolution imagery to study urban sprawl. Instead we focus on how built-up areas change over time for the entire country of Ghana, with a special and novel focus on partially built-up cells. The GHSL data at finer resolution (38mX38m) only identify if a cell has a building or not but not whether a cell is partially built. Partially built-up cells can only be identified at a coarser resolution. Our analysis focuses on the proportion of a 250mx250m cell that is

built-up, as reconstructed by the Joint Centre of the European Commission on the basis of AI predictions for 38mX38m cells.

We now realize that our interpretation of partially built-up cells as an indicator of urban sprawl caused confusion. The phrase 'urban sprawl' seems to be used by many as synonymous to the spatial growth of towns and cities. We were using instead it as synonymous to the growth of the partially built 'crown' of cities. This was probably a mistake. To avoid further confusion, we have since removed the phrase 'urban sprawl' from the revised version and we have further clarified the interpretation of our findings. We have also added this explanation to a footnote in the data sub-section.

3. Provide a "materials and methods" section with detailed descriptions of the study area, and steps used to accomplish the analysis. A majority of contents in results belong to the materials and methods section.

We rewrote the Materials and Methods section entirely as per the reviewers' suggestions. This section is now divided into three subsections: a conceptual framework to provide insights from existing economic theories on how buildup areas may evolve overtime; a data sub-section that describes in detail the construction of the data used in the study; and an empirical method sub-section that collects in one place various estimation details that were scattered in results section before.

4. Currently, the manuscript draft reads like a report. Restructure various sections with substantive information to flow like a research article.

We have rewritten most of introduction and conclusions to make the paper flow like an article. It never was a report, by the way.

5. Provide a descriptive caption for each figure.

This was already done for all Figures except Appendix Figures A1 to A4. We have now added detailed explanation for these Figures as well. Thanks for noting this oversight.

We attach below a detailed response to each of the referees' comments. We believe we have adequately addressed them all, to the extent allowed by the data. We also made an honest effort to respond to all of reviewer #3's comments, even though most of them are insufficiently elaborated for us to take very specific action. We believe the paper is now ready for publication.

Reviewer #1: Review: The Evolution of Built-up Areas in Ghana since 1975

The paper analyses the evolution of the built-up area in Ghana since 1975. The data and analysis make for good reading and are primarily sound. There are, however, three areas that I think are problematic (and the editor can decide how important this is for PLOS ONE).

• There is a minimal reference to similar work in Africa. I know of a paper published in "Nature" that did similar work for Africa.

We expanded our review of the literature, which led us to add 13 more references – including to papers published in Nature, Nature Sustainability, and Nature Communications.

• The paper is also not situated within the existing body of work. In addition, the paper could do more to situate the context within the housing research of Africa. Why is it important to study these aspects in Africa? The paper indeed references some of Henderson's work, but there has been some recent work on the state of housing policy research in Africa.

Our data are not specific enough to relate our analysis directly to the state of the housing situation. We now mention the importance of providing adequate housing for the urban population and we have added four new references on this topic, including a review paper.

• Linked to the above point, the interpretation and contextualisation (including the conceptual framework) of the work in housing policy and housing policy analysis in Africa seems weak. Often statements are made without much evidence and context – for example, the statement on urban sprawl in Ghana seems debatable when I look at the data. The question is, what are the 3-4 key issues of the paper – the existing overview is not good enough and haphazard to my mind.

We now realize that our interpretation of partially built-up cells as an indicator of urban sprawl caused confusion. The phrase 'urban sprawl' is sometimes used as synonymous to the spatial growth of towns and cities. We were using instead it as synonymous to the growth of the partially built 'crown' of cities. This was probably a mistake on our part. To avoid confusion, we have since removed the phrase 'urban sprawl' from the revised version and we have further clarified the interpretation of our findings. We have also added an explanatory footnote in the data sub-section. We agree that our results confirm that the growth of built-up areas in Ghana is consistent with the expansion of urban land cover: we indeed discuss in the paper the large increase in high density built-up areas over the study period, and we predict further increases in the future. However, the constancy of proportion of partially built-up areas implies that the expanding population of Ghana is congregating in urban areas instead of spreading out over the geographical space. Regarding the last point, we have clarified in the first paragraph of the paper the three key research questions that we address in the paper.

• The section on Von Thunen's as theoretical frame is not convincing. Surely times have changed – even in rural Africa. Alternatively, a more precise argument needs to be created around this issue

We rewrote parts of the paper where von Thunen's framework is mentioned. In particular, we have added more explanation about why it would important to know if cities and towns are regularly spaced because this would help to plan the geographical provision of infrastructure and services.

Reviewer #2:

This study examined and described the Evolution of Built-up Areas in Ghana since 1975. The analysis carried out are very detailed and interesting whilst the results/findings in general are promising. I believe the outputs might be important for urbanists and other spatial planners. However, there is more room for improvement in many aspects of the "manuscript". Therefore, before I can recommend it for acceptance and publication, it needs some major revisions.

May I advise that Authors pay attention to the following comments and suggestions including those provided in the manuscript (see attached pdf file).

1. I am afraid the submitted article simply does not read like a scientific paper. It reads more or less like a report of a sort. There is a distinct only provided a detailed description of the data used.

The paper has been reorganized in the format of a PLOS ONE paper. We have brought all the description and discussion of empirical methods under the "Material and Methods" Section, as per the reviewer's suggestion. This section is now divided into three subsections: a conceptual framework to provide insights from existing economic theories on how buildup areas may evolve overtime; a data sub-section that describes in detail the construction of the data used in the study; and an empirical method sub-section that collects in one place various estimation details that were scattered in results section before.

2. The first part of the introduction was well written although the justification for this study was not very clear. My main concern however, is the latter part of the introduction which reads more or less like an "executive summary" of the study's findings. Well, some of the key findings that have been presented in the results and discussion section were just repeated in the introduction and I simply do not understand why.

The second part of the introduction has been rewritten. It provides a brief description of the key findings and more explanation on how they relate to the existing literature.

3. I am not too sure why the Von Thunen's "agricultural land use" theory was chosen to study the "Evolution of Built-up Areas". Maybe authors could explain and help us understand this better. As a geographer who has a broad knowledge of the Von Thunen's theory, I do not think such a theory was appropriate for this study. I stand to be corrected though.

In response to population growth, new towns and cities may emerge. One of the three main questions that we examine is whether towns and cities are regularly spaced relative to each other. The advantage of the regular spacing of towns and other human settlements is that it simplifies regional planning by allowing some forecasting of future settlement patterns, thereby facilitating the position of infrastructure such as roads, electrical lines, industrial parks, and other land development schemes. While the original insights of von Thunen go back to the early 19th century, they were revisited and expanded by Christaller and Isaard to predict, under certain conditions (e.g. the full utilization of agricultural land), the regular spacing not only of rural settlements, but also of towns of various sizes. To be clear, we are not checking for pattern of agricultural land use. We are looking for regularity in the spacing of human settlements, either on a plane or along roads, and we do so at different points in time to detect changes in this pattern, e.g., as a result of an increase in population density and thus in the utilization level of agricultural land. We have added more explanation in the introduction to make this clear.

4. Also in the introduction, authors have indicated that the the paper contributes to the literature on urbanization and regional development citing Seto et al. 2011 and Henderson and Turner 2020 as reference literature. However, authors failed to elaborate on this. I was expecting some elaboration of this argument either in the discussion or even conclusion section. Authors should tell us what the literature on urbanization and regional development is saying; what and how is their present study contributing to it. As it is, the knowledge that is being added is not clearly spelt out.

In response to the referee, we have added more detail on the main findings presented in these papers. In addition, we have added several new references and we now better relate our findings to the existing literature.

5. Methodology wise, the article doesn't provide any structure for readers to follow. In fact, there is no section devoted to materials and methods as stipulated in the authors guideline of PLOS ONE. Because of this, many of what can be described as methodological procedures have been presented in the results and discussion section. That is unacceptable in scientific work. May I suggest to the authors that they do their best to revisit the manuscript organisation page of PLOS ONE to see how their manuscript is expected to be structures: https://journals.plos.org/plosone/s/submission-guidelines#loc-parts-of-a-submission.

This is an important point, and we have taken it to heart. As mentioned in our response to point 1, we have brought all the description and discussion of empirical methods under the "Material and Methods" Section, as per the reviewer's suggestion. This section is now divided into three subsections: a conceptual framework to provide insights from existing economic theories on how buildup areas may evolve overtime; a data sub-section that describes in detail the construction of the data used in the study; and an empirical method sub-section that collects in one place various estimation details that were scattered in results section before. We apologize for not doing this earlier.

6. Although an elaborate results and discussion has been presented, it lacks understanding. Authors should clearly tell us what their results/findings imply or better still what the findings mean. As it it now, I simply cannot make any meaning out of what has been presented. All the methodological issues presented in this section should be moved to a "materials and methods" section.

All the materials related to the methodology have been moved to the "Materials and Methods" Section (Section 2), as per PLOS ONE guidelines. We have also added more explanations about the main findings throughout the paper.

7. The conclusion is too vague and lacks any substantive or analytical arguments in support of the key findings.

We have completely rewritten the conclusion. It now briefly summarizes the main findings of the paper and discusses their policy implications.

Reviewer #3:

Hi Author

Thank you for the privilence to review this paper and below are some of my pointer why i think the paper is not ready for publication

1. The paper is poorly structured.

The paper has been restructured according to the outline provided by PLOS ONE.

2. The paper is too long

Some of the details have been shortened and excluded from the introduction and conclusion. The total length of the paper remained largely unchanged, however: the other reviewers and the editor asked us to provide more explanation and additional references.

3. There are not references in the paper (the athors were just giving an example of the paper), How did you cite your references?

We have rewritten the introduction to describe in some more detail the nature of the evidence presented in the papers we cite.

4. 250 metre resoloution for urban studies is not accurate enough

We have clarified in the text that our paper is not trying to use high resolution imagery to study urban sprawl. Instead we focus on how built-up areas change over time for the entire country of Ghana, with a special and novel focus on partially built-up cells. The GHSL data at finer resolution (38mX38m) only identify if a cell has a building or not but not whether a cell is partially built. Partially built-up cells can only be identified at a coarser resolution. Our analysis focuses on the proportion of a 250mx250m cell that is built-up, as reconstructed by the Joint Centre of the European Commission on the basis of AI predictions for 38mX38m cells.

We also realize that our interpretation of partially built-up cells as an indicator of urban sprawl may have caused confusion. The phrase 'urban sprawl' is sometimes used as synonymous to the spatial growth of towns and cities. We were using instead it as synonymous to the growth of the partially built 'crown' of cities. This was probably a mistake. To avoid confusion, we have since removed the phrase 'urban sprawl' from the revised version and we have further clarified the interpretation of our findings. Our results confirm that the growth of built-up areas in Ghana is consistent with the expansion of urban land cover: we indeed discuss in the paper the large increase in high density built-up areas over the study period, and we predict further increases in the future. However, the constancy of proportion of partially built-up areas implies that the expanding population of Ghana is congregating in urban areas instead of spreading out over the geographical space.

5. Is the author is writing in first person or third person? There is a confusion in that regards

Thanks for noting this inconsistency. It has now been corrected.

6. The reporting is not formal and its not good for academic publication.

We are not sure what specific issue the referee has in mind, but we have made many substantial improvements to the paper that, we believe, make it now suitable for publication.

7. The authors must work on the flow of the paper.

This is an important point that we have taken to heart. We have now brought all the description and discussion of empirical methods under the "Material and Methods" Section, as per the reviewer's suggestion. This section is now divided into three subsections: a conceptual framework to provide insights from existing economic theories on how buildup areas may evolve overtime; a data sub-section that describes in detail the construction of the data used in the study; and an empirical method sub-section that collects in one place various estimation details that were scattered in results section before. We have also rewritten the introduction and conclusion and made many improvements to the results section to clarify the meaning of our findings.

8. The different font types and font sizes needs to be looked into

Thanks. This has now been corrected – and will be further harmonized by the publishing editor.

9. The digrams are not clear and are not communciating with the main text.

We've added detailed notes to Appendix Tables A1 to A4, as requested. All the other Tables and Figures already had detailed explanatory notes.

10. if the lines on the paper were paper I was going to provide comments on each and every section with proper reference to the line number.

Hope this is clear.

Reviewer #4:

This a is well-written paper that contributes significantly to the literature on urbanisation and urban transformation. The methodology is sound and replicable. The findings and conclusion are logically presented.

Thank you very much for your kind support.

Comments on the pdf copy of paper

1. "This means that, based on current trends, we do not expect urban growth in Ghana to take the form of urban sprawl." But what is the timeline to this finding? moreover, there are several studies which have shown otherwise depending on the time.

2. "Urban sprawl" : I am really finding it difficult to agree with this finding because there are numerous studies including a study I have conducted myself which shows that at least Accra and Kumasi are experiencing massive urban sprawl. Please help me understand this. Clear evidence of urban sprawl in many cities i Ghana has been reported by several urbanists in Ghana.

   We realize that our interpretation of partially built-up cells as an indicator of urban sprawl may have caused confusion – indeed this point was mentioned in one way or another by three referees! We are grateful to the referees for bringing this to our attention since it would have undoubtedly confused readers as well.

   The phrase 'urban sprawl' is used by the referee as synonymous to urban growth over space. We were using instead it as synonymous to the growth of the partially built 'crown' of cities. This was probably a mistake. To avoid further confusion, we have since removed the phrase 'urban sprawl' from the revised version and we have further clarified the interpretation of our findings. Our results confirm that the growth of built-up areas in Ghana is consistent with the expansion of urban land cover: we indeed discuss in the paper the large increase in high density built-up areas over the study period, and we predict further increases in the future. However, the constancy of proportion of partially built-up areas implies that the expanding population of Ghana is congregating in urban areas instead of spreading out over the geographical space.

3. "UN 2013" introduction

This is outdated! We live in the 21st Century and so it will be good to have discussion around that. Please update this - you are talking about prediction, yet reporting something as old as 2013?

   This has been corrected. We now use the forecast from the UN DESA (2019) report which, to our knowledge, contains the most recent UN population forecasts.

4. "Growth of small cities" This is something that has already been established by the World Bank Group (2015). "Rising through Cities in Ghana"

   Thanks for pointing this out to us. This reference has been added in the introduction.

5. Volta dam:

Since we have taken out of the introduction the paragraph with the offending statement, the comment no longer applies.

6. "I am really struggling to understand why statements such as this - which are methodological issues would be presented in the results/discussion section."

Good point. We have now brought all the description and discussion of empirical methods under the "Material and Methods" Section, as per the reviewer's suggestion. This section is now divided into three subsections: a conceptual framework to provide insights from existing economic theories on how buildup areas may evolve overtime; a data sub-section that describes in detail the construction of the data used in the study; and an empirical method sub-section that collects in one place various estimation details that were scattered in results section before.
